# Antifibrinolytic Agent: Synthesis and Characterization of Cocrystal and Salts of Tranexamic Acid

**DOI:** 10.1021/acsomega.6c03598

**Published:** 2026-10-22

**Authors:** Bolaji C. Dayo Owoyemi, Adedamola E. Adehinmoye, Matthias Zeller, Gabriel L. B. de Araujo, Stephen R. Byrn, Kari Clase

**Affiliations:** † Department of Industrial and Molecular Pharmaceutics, College of Pharmacy, 311308 Purdue University, 575 Stadium Mall Drive, West Lafayette, Indiana 47907, United States; ‡ Department of Pharmacy, Faculty of Pharmaceutical Science, University of São Paulo, Av. Professor Lineu Prestes, 580—Cidade Universitária, São Paulo CEP 05508-000, SP, Brazil; § Department of Chemistry, 311308 Purdue University, 560 Oval Drive, West Lafayette, Indiana 47907, United States; ∥ Department of Agriculture and Biological Engineering, 311308 Purdue University, 225 S. University Street, West Lafayette, Indiana 47907, United States

## Abstract

Tranexamic acid (TXA) is a potent antifibrinolytic agent with poor oral bioavailability (∼35–40%) due to its high polarity and zwitterionic nature, while physicochemical factors, including its structural instability, further hinder its absorption. In this study, the crystal engineering concept is applied to enhance the physicochemical properties of TXA through targeted structural modifications, resulting in the synthesis of pharmaceutically stable cocrystal and salt forms. The application of supramolecular synthon design, p*K*_a_ rule, and structural geometry (molecular mimicry), resulted in the rational design, synthesis, and structural characterization of eight binary multicomponent forms of TXA: a *TXA-adipate salt*, monoclinic space group *P2*_1_/*n*; a *TXA-salicylate salt*, monoclinic space group *P2*_1_/*n*; a *TXA-picolinate salt*, monoclinic space group *P2*_1_/*c*; a *TXA-acetaminophen cocrystal hydrate*, monoclinic space group *P2*_1_; a *TXA-malonate salt*, orthorhombic space group *Pbca*; a TXA-*maleate salt hydrate*, monoclinic space group *P2*_1_/*c*; a *TXA-malate salt*, monoclinic space group *P2*_1_/*c*; and a *TXA-citrate salt*, triclinic space group *P*1̅ These multicomponent forms were obtained via different hydrogen bonding interactions, some of them charge-assisted (N–H···O, N^+^–H···O^–^, O–H···O, and O–H···O^–^) between NH_2_ and/or −OH groups of TXA and the carboxyl (−C­(O−)=O) functional groups of the coformers. In the cocrystals, TXA exists as zwitterions (zero charge), whereas in the salts, it carries a positive charge due to the protonation of its carboxylic (−COOH) and ammonium (−NH_3_^+^) groups. The NH_3_^+^ to COO^–^ charge-assisted H-bond provides stabilization to both the coformer COO^–^ as well as the TXA’s NH_3_^+^. Solid-state characterization using Raman and FT-Raman spectroscopy, thermal techniques (DSC), and X-ray diffraction analysis (powder and single-crystal XRD) supports and confirms the structural novelty. The superior stability profiles of the new TXA cocrystal and salts under standard conditions (40 °C ± 2 °C, 75% ± 5% RH) establish an improved structure–property profile compared to pure TXA.

## Graphical abstract

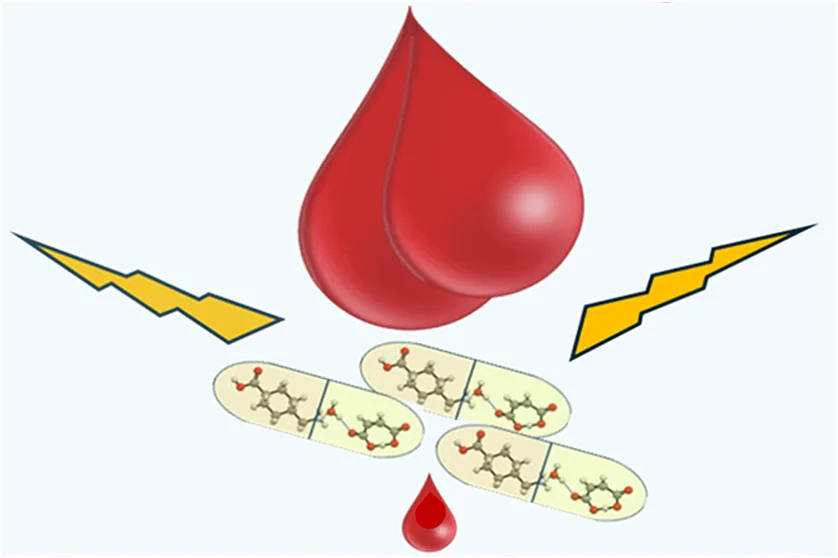

## 1 Introduction

Tranexamic acid: (trans)-4-(aminomethyl)­cyclohexane-1-carboxylic acid is a synthetic derivative of lysine and an antifibrinolytic drug approved by the U.S. Food and Drug Administration (FDA) and the World Health Organization (WHO) to treat excessive blood loss during cases of trauma, surgery, and conditions like hemophilia, menorrhagia, and postpartum hemorrhage (PPH). Despite its inclusion on the WHO Essential Medicines List and use alongside uterotonics for PPH, maternal mortality remains high, with PPH causing 27% of global maternal deaths (∼60 deaths per 100,000 births in 2023) and severe complications like anemia and organ failure.^1–4^ Tranexamic acid (TXA) works by competitively inhibiting plasminogen activation, thereby stabilizing fibrin clots to reduce bleeding. It acts rapidly, has a short half-life (2–3 h), and is primarily excreted unchanged (>95%) in urine.^5,6^ The Purdue Biotechnology Innovation and Regulatory Sciences (BIRS) program has held several sessions and symposia in Africa during our MS and certificate programs addressing TXA and the exciting therapeutic opportunities it offers for treating PPH in sub-Saharan Africa because of its stability at ambient African conditions.^7,8^ One of the most interesting strategies for dealing with the high death rate from PPH in sub-Saharan Africa is the utilization of TXA in combination with Oxytocin.^9,10^ However, there has been concern raised in publications that mixing TXA with oxytocin reduces the stability of Oxytocin, thus discouraging this strategy. One concern is that TXA impurities degrade Oxytocin.^11–13^

TXA is a BCS class I drug with high aqueous solubility (∼200 g/L), but with p*K*_a_ values of 4.3/10.6. It is present at neutral pH in its zwitterionic form. This leads to high polarity, hygroscopicity, and low lipophilicity associated with TXA’s poor gastrointestinal absorption and limited oral bioavailability (30–40%), which hinders its rapid emergency use and delays its therapeutic onset in emergencies.^14–17^ Cases of treatment failure due to low absorption and poor bioavailability of TXA are reported.^17–20^ In its crystalline form, TXA exists as a metastable orthorhombic (*P*2_1_2_1_2_1_) under ambient conditions,^21^ presents a melting point between 386 and 388 °C, and has a 3–5 year shelf life.^22^ One strategy to address the impurity and property issues of TXA is to explore alternate multicomponent solid forms including salts and cocrystals. Several multicomponent forms of TXA have been reported previously. Kadoya et al. (1966) first described the crystal structure of pure TXA.^23^ Nechipadappu et al. (2018, 2023) reported salts and cocrystals of TXA with various GRAS coformers, including maleate hydrate, picolinate, and salicylate.^15,22^ Ezekiel et al. described an ionic cocrystal of TXA with 2,4-pyridinedicarboxylic acid.^24^ Frösler et al. synthesized salts of TXA with ibuprofen.^25^ While these studies demonstrated the feasibility of modifying TXA’s solid-state properties, they were conducted under different experimental conditions, making direct comparisons of supramolecular motifs, thermal behavior, and physical stability more difficult.^22,25,26^ Crystal engineering provides a rational framework to enhance the physicochemical properties of poorly performing active pharmaceutical ingredients (APIs) through cocrystallization and salt formation, by exploiting supramolecular synthon interactions and crystal packings to modulate solid-state properties.^27–29^ The crystal engineering approach leverages noncovalent interactions, synthon design, and p*K*_a_-driven selection (Δp*K*_a_ ≤ 1 for cocrystals; Δp*K*_a_ ≥ 3 for salts) to modulate solubility, stability, and permeability criteria.^29,30^ Cocrystal and salt production continue to gain prominence in pharmaceutical development due to their often enhanced drug performance, tunable pharmacokinetics, and established safety profiles.^31–35^

In this work, we report the first systematic, multicomponent structural library of TXA with a family of carboxylic acids (adipic, malic, malonic, maleic, picolinic, salicylic, citric, and acetaminophen) using identical crystallization, characterization, and stability assessment protocols. Five of these forms, TXA–adipate (2:1 salt), TXA–picolinic (1:1 salt), TXA–malate (2:1 salt), TXA–citrate (1:1 salt), and TXA–APAP (3:1 cocrystal hydrate), are new and have not been previously reported. Although the maleate hydrate and picolinate salt of TXA have been previously reported, precluding a novelty claim, their inclusion enables quantitative comparison of supramolecular motifs, thermal behavior, and physical stability under identical conditions across all eight TXA multicomponent forms. Structural characterization was achieved by powder and single-crystal XRD analysis, confirming material novelty, qualitative phase purity and identification, and crystal structure determination/depiction. Further characterization using differential scanning calorimetry (DSC) enables the determination of phase transitions, melting points, and other thermal-related events. Finally, the stability under accelerated conditions (40 °C ± 2 °C, 75% ± 5% RH, 6 months) was assessed using PXRD to establish stability and structure–property relationships.

## 2 Experimental Section

### 2.1 Materials

Tranexamic acid (TXA) and coformers: Malic (MA), Malonic (MAL), Maleic (MLE), Picolinic (PIC), Adipic (ADP), Acetylsalicylic (ASA), Acetaminophen (APAP), and citric acid (CTR) (Figure 1) were purchased from Sigma-Aldrich at >99% purity. The materials’ identity and purity were confirmed by powder X-ray diffraction. Solvents used: methanol (MeOH), acetonitrile (ACT), and acetone (Ace) were purchased from J.T. Bakers Ltd. at an HPLC grade (>99.5%). Organic reagent: HCl 37% purchased from J.T. Bakers Ltd. All chemicals were used as received without further purification.

**Figure 1:**
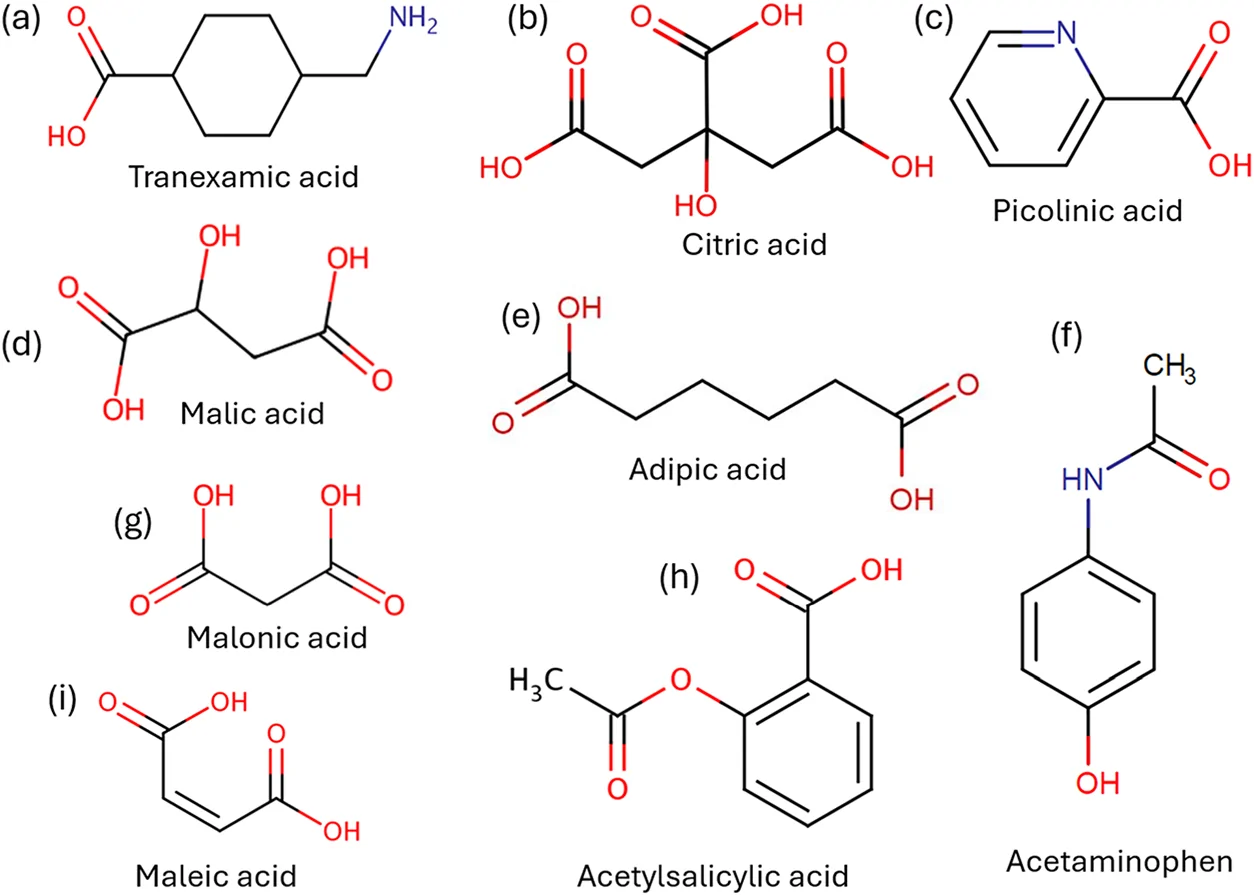
Sketched representation of the chemical structure of tranexamic acid and coformers.

### 2.2 Preparation by Mechanochemical Methods and Screening

**2**.**2**.**1**. TXA (15.721 mg, 0.10 mmol) were combined separately with 0.10 mmol of each of the coformers (Figure 1); MA (13.41 mg), MAL (10.41 mg), MLE (11.61 mg), PIC (12.31 mg), ADP (14.61 mg), ASA (18.02 mg), APAP (15.12 mg), and CTR (19.21 mg), at mass ratios (TXA:coformer) of 1:1, 1:2, and 2:3. The mixtures were milled using a mortar and pestle for 15 min (grinding, G), and to another weighed samples 3 drops of methanol were added during the milling process (solvent assist grinding, SAG). These processes were repeated for all combinations. Spectroscopy (Raman and FT-IR) and PXRD screening confirmed the formation of new phases distinct from the starting materials.

### 2.3 Preparations by Slow Solvent Evaporation Solution Methods

#### 2.3.1 TXA–MA (2:1) Salt

TXA (314.42 mg, 2.00 mmol) and MA (134.10 mg, 1.00 mmol) were weighed into a beaker (20 mL) and dissolved using methanol (10 mL). The mixture solution was heated at 55 °C and stirred for 5 min, and the beaker was covered with perforated polythene to let the solvent evaporate slowly at RT. Crystals (∼425 mg/>90%) were obtained after 3 days.

#### 2.3.2 TXA–CTR (1:1) Salt

TXA (157.21 mg, 1.00 mmol) and CTR (192.10 mg, 1.00 mmol) were weighed into a 20 mL beaker and dissolved using methanol (10 mL). The solution was lightly heated (45 °C), stirred for 6 min, and covered with perforated polythene to let the solvent evaporate at RT. Crystals (285.16 mg/>90%) were obtained after 3 days.

#### 2.3.3 TXA–APAP (3:1) Cocrystal Hydrate

TXA (157.21 mg, 1.00 mmol) and APAP (453.48 mg, 3.00 mmol) were weighed into a 20 mL beaker and dissolved using a 20 mL aqueous methanol solution (50% v/v, methanol:water). The solution was heated to 55 °C, stirred for 8 min, and covered with perforated polythene to let the solvent evaporate slowly at RT. Crystals (588.50 mg/>90%) were collected from water after 4 days and dried in air.

#### 2.3.4 TXA–PIC (1:1) Salt

TXA (157.21 mg, 1.00 mmol) and PIC (123.10 mg, 1.00 mmol) were weighed into a 20 mL beaker and dissolved using methanol (10 mL). The solution was heated to 50 °C, stirred for 10 min, and covered with perforated polythene to let the solvent evaporate at RT. Crystals (272.57 mg/>90%) were obtained after 4 days.

#### 2.3.5 TXA–MAL (1:1)

TXA (157.21 mg, 1.00 mmol) and MAL (104.10 mg, 1.00 mmol) were weighed into a 20 mL beaker and dissolved using methanol (10 mL). The solution was heated (50 °C), stirred (10 min), and covered with perforated polythene to let the solvent slowly evaporate at RT. Crystals (∼240 mg/>90%) were obtained after 3 days.

#### 2.3.6 TXA–SAL (1:1)

TXA (157.21 mg, 1.00 mmol) and ASA (180.20 mg, 1.00 mmol) were weighed into a 20 mL beaker and dissolved using methanol (10 mL) and water (5 mL). The solution was slightly heated (50 °C), stirred for 10 min, and covered with perforated polythene for slow solvent evaporation at RT. Crystals (∼276 mg/> 95%) were obtained after 3 days, filtered, and dried. ASA hydrolyzed in situ during the crystallization process, yielding crystalline TXA–SAL salt, as confirmed by both single-crystal and powder XRD.

#### 2.3.7 TXA–ADP (2:1) salt

TXA (314.42 mg, 2.00 mmol) and ADP (146.10 mg, 1.00 mmol) were added to a 20 mL beaker and dissolved using 16 mL of a 50% (v/v) methanol–water solution. The mixture was gently heated to 50 °C while stirring for 10 min, then covered with perforated polythene film, and the solvent was allowed to evaporate slowly at room temperature. After 3 days, crystals were obtained (∼450 mg, yield >90%).

#### 2.3.8 TXA–MLE (1:1) monohydrate salt

TXA (157.21 mg, 1.00 mmol) and MLE (116.10 mg, 1.00 mmol) were weighed into a 20 mL beaker and dissolved in 20 mL of a 50% (v/v) methanol–water solution. The solution was slightly heated (50 °C), stirred for 10 min, and covered with perforated polythene to let the solvent evaporate slowly at RT. Crystals (∼250 mg/>90%) were obtained after 4 days.

### 2.4 FT-Raman Spectroscopy

Raman spectra acquisition, screening, and characterization were performed using an FT-Raman Bruker RFS 100 spectrometer equipped with a liquid nitrogen-cooled germanium detector and a 1064 nm excitation source (Nd:YAG laser, Coherent Compass 1064–500N). Spectra were recorded in the wavenumber range of 4000 to 200 cm^–1^ at room temperature with 150 mW laser power, accumulating 256 scans at a spectral resolution of 4 cm^–1^.

### 2.5 Raman Microscopy

Screening and spectra acquisitions were performed using a Renishaw inVia confocal Raman microscope equipped with a 785 nm excitation laser (diode) and a deep-depletion CCD detector. Spectra were recorded in the wavenumber range of 3200 to 100 cm^–1^ at RT with 5 mW laser power at 10 s exposure time per scan. Rayleigh rejection filters enabled low-frequency measurements down to 100 cm^–1^. The spectral data were processed using WiRE 5.x software.

### 2.6 Powder X-ray Diffraction (PXRD)

X-ray diffraction analysis of the powdered samples was performed on a Multiplex X-ray diffractometer (Rigaku Corporation, Tokyo, Japan) using Cu Kα radiation (λ = 1.5406 Å) at an operating voltage of 40 kV and a current of 30 mA. Diffraction patterns were collected over a 2θ range of 4° to 50° with a step size of 0.2° and a scanning speed of 2° per minute. The data obtained were used to evaluate the sample crystallinity and purity.

### 2.7 Differential Scanning Calorimetry (DSC)

A DSC 2500 was used, and samples were heated from 25–350 °C at a heating rate of 10 °C min^–1^ in a crimped sealed aluminum pan, using nitrogen as purge gas (100 mL min^–1^).

### 2.8 Stability Studies

Stability studies were carried out under 75 ± 5% relative humidity (RH) at 40 ± 2 °C for 6 months to monitor phase transitions. The samples were weighed and placed in a desiccator containing a saturated sodium chloride (NaCl) solution to maintain RH. PXRD (Powder X-ray Diffraction) of the samples was performed every month.

### 2.9 Single Crystal X-ray Diffraction (SCXRD)

The crystallographic data for all the TXA cocrystals and salts were collected on a Bruker AXS D8 Quest diffractometer with a Photon III C14 detector using Cu Kα radiation (λ = 1.54178 Å), an I-μ-S microfocus X-ray tube, and laterally graded multilayer (Goebel) mirrors for monochromatization.

The instrument was equipped with an Oxford Cryosystems low-temperature device, and data were collected at RT, or 150 K. Data were collected using Bruker Apex4 or Apex5 and BIS software.^36^

### 2.10 Single Crystal X-ray Data Collection, Refinement, and Structure Determination

The single crystals of TXA salts/cocrystals suitable for analysis were dispersed in Fomblin oil and investigated using a polarized light microscope before being mounted to the goniometer head of the Bruker AXS D8 Quest diffractometer. Reflection data were collected using the Apex4/Apex5 software.^36^ The CSD^37^ was checked after unit cell determination to avoid redundant data collection. For compounds with novel unit cell parameters, full data were collected, data were processed, and the files were scaled and corrected for absorption using Apex4/Apex5 ^38^ and SADABS.^39^ The space groups were assigned using XPREP in the SHELXTL suite of programs.^40,41^ The structures were solved by dual methods using ShelXT^42^ and refined by full-matrix least-squares against F^2^ with all reflections using SHELXL-2019/2 via the graphical interface ShelXle.^43–45^

All H atoms attached to carbon and hydroxyl hydrogens were positioned geometrically and constrained to ride on their parent atoms. C–H bond distances were fixed at 0.95 Å for aromatic, alkene, CH_2_, and alkyne moieties, and at 1.00, 0.99, and 0.98 Å for aliphatic C–H, CH_2_, and CH_3_ moieties, respectively. Unless otherwise specified, O–H distances of alcohols were allowed to refine, but the angle was constrained, and ammonium N–H distances were constrained to 0.91 Å. Methyl (CH_3_), ammonium (NH_3_^+^), and hydroxyl H atoms were permitted to rotate (but not to tip) to optimize agreement with experimental electron density. *U*_iso_(H) values were assigned as multiples of *U*_eq_(C/N/O) with 1.5 for CH_3_ and OH, and 1.2 for C–H, CH_2_, and N–H units, respectively.

#### TXA-MA

For MA, the hydroxyl group (OH) is 1:1 disordered over the two symmetry-equivalent positions of C10 and its counterpart by inversion. Alcohol H atom positions were freely refined.

#### TXA-PARA

The structure is metrically close to orthorhombic and is twinned by this symmetry. Application of the twin matrix 1 0 0 0 −1 0 −0.048 0 −1 yielded a BASF value of 0.1772(17). Water H atom positions were refined, and O–H and H···H distances were restrained to 0.84(2) and 1.36(2) Ångström, respectively.

#### TXA-PIC

The disorder of the cyclohexyl ring of TXA is observed due to ring inversion. Minor twinning by a 180° rotation around reciprocal (1 0 0) was observed. Including twinning (as either HKLF 5 or 4) did not improve data or refinement, and the best *R* values, completeness, or the esds of atomic parameters were obtained under omission of twinning (omission of the minor domain during integration). The cation is disordered by inversion of the cyclohexyl ring. The ammonium N atom and O1 of the carboxylic acid group were omitted from the disorder. The two disordered moieties were restrained to have similar geometries (SADI and SAME commands of Shelxl). *U_ij_* components of ADPs for disordered atoms closer to each other than 2.0 Ångström were restrained to be similar. ADPs of O2 and O2B were constrained to be identical. Subject to these conditions, the occupancy ratio was refined to 0.900(3) to 0.100(3). The position of the carboxylic acid proton was freely refined.

*TXA-CTR*, *TXA-MAL*, *TXA-MLE*, and *TXA-ADP* show no disorder. Ammonium and alcohol H atom positions were freely refined. Water H atom positions in *TXA-MLE* were freely refined.

#### TXA-SAL

X-ray analysis showed that in the crystal analyzed, Acetylsalicylic acid (ASA) underwent an *in situ* chemical transformation and hydrolyzed into salicylic acid (SAL) and acetic acid due to cleavage of the ester bond and breaking of the acetyl-oxygen bond, particularly in the presence of residual moisture. Incorporated in the crystal was salicylate. Therefore, this single crystal was renamed as TXA-SAL (salicylic acid), and all the single crystal parameters were generated for TXA-SAL. Powder XRD analysis of the bulk material matches the single crystal structure of TXA-SAL, indicating complete hydrolysis of ASA to SAL. The dominant and well-ordered species in the crystal lattice is a 1:1 salt formed between TXA and the generated SAL. The acetic acid byproduct of hydrolysis is not present in the refined model and is not part of the primary crystal architecture.

SHELXTL^43^ and ORTEP^46^ software programs were employed to prepare the structure artwork (Figure 2). Table 1 shows detailed crystallographic data and structure refinement parameters for the TXA salt and cocrystal structures. The CIFs of these new structures were deposited under the codes **CCDC: 2435653** (*tranexamic acid-malate salt*), **2435259** (*tranexamic acid-acetaminophen hydrate*), **2435644** (*tranexamic acid-picolinate salt*), **2435639** (*tranexamic acid-malonate salt*), **2435624** (*tranexamic acid-citrate salt*), **2435619** (*tranexamic acid-salicylate salt*), **2435648** (*tranexamic acid-adipate salt*), and **2435659** (*tranexamic acid-maleate salt hydrate*). Copies of these files may be accessed in the CCDC via www.ccdc.cam.ac.uk/data_request/cif. Additional geometrical parameters, data collection, and refinement details are available in the Supporting Information (SI).

**Figure 2:**
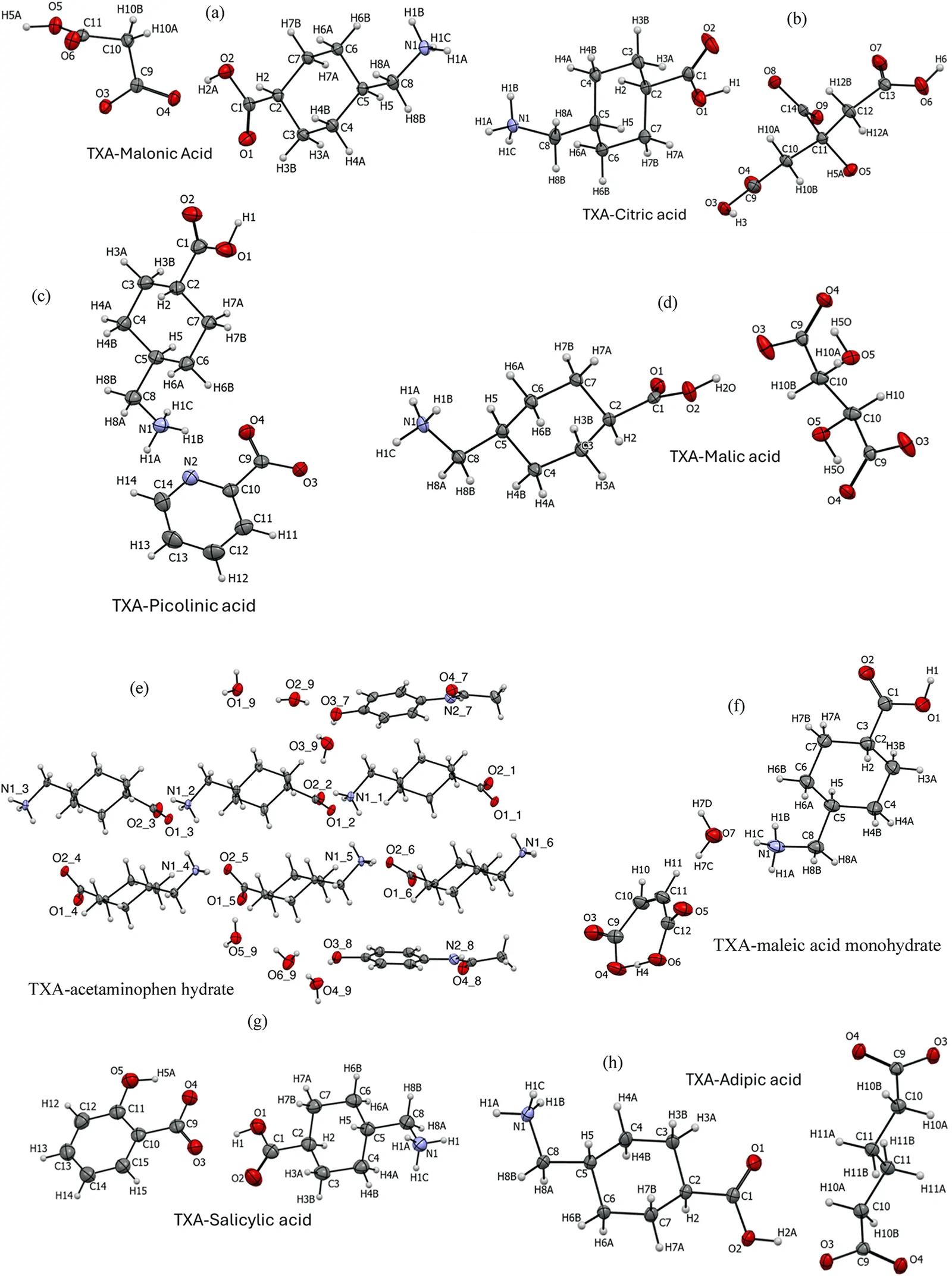
ORTEP representation of the asymmetric units of Tranexamic acid (TXA) multicomponents (50% probability displacement ellipsoids). The labels for C and H atoms in TXA-acetaminophen hydrate (e) are omitted for clarity.

**Table 1:** (A) Experimental Details, Crystallographic Data, and Structure Refinement Parameters (B) Experimental Details, Crystallographic Data, and Structure Refinement Parameters^a^

	TXA-MA	TXA-SAL	TXA-PIC	TXA-APAP hydrate	TXA-MAL
CCDC number	**2435653**	**2435619**	**2435644**	**2435259**	**2435639**
Empirical formula	C_20_H_36_N_2_O_9_	C_15_H_21_NO_5_	C_14_H_20_N_2_O_4_	C_32_H_60_N_4_O_11_	C_11_H_19_NO_6_
Moiety formula	2(C_8_H_16_NO_2_)·C_4_H_4_O_5_	C_8_H_16_NO_2_·C_7_H_5_O_3_	C_8_H_16_NO_2_·C_6_H_4_NO_2_	3(C_8_H_15_NO_2_)·C_8_H_9_NO_2_·3(H_2_O)	C_8_H_16_NO_2_·C_3_H_3_O_4_
Formula weight	448.51	295.33	280.32	676.84	261.27
Temperature [K]	150(2)	150(2)	150(2)	150(2)	150(2)
Crystal system	monoclinic	monoclinic	monoclinic	monoclinic	orthorhombic
Space group (number)	*P*2_1_/*c* (14)	*P*2_1_/*n* (14)	*P*2_1_/c (14)	*P*2_1_ (4)	*Pbca* (61)
*a* [Å]	10.3014(3)	9.0893(8)	11.0221(17)	6.3475(2)	10.2415(2)
*b* [Å]	8.6021(2)	13.9859(12)	6.5885(9)	23.4719(7)	9.5341(2)
*c* [Å]	12.8214(3)	11.9845(11)	20.098(3)	23.6876(7)	25.7552(6)
α [°]	90	90	90	90	90
β [°]	109.3912(8)	105.219(5)	95.693(6)	90.3581(15)	90
γ [°]	90	90	90	90	90
Volume [Å^3^]	1100(5)	1470.1(2)	1450(4)	3529.10(19)	2514.83(9)
*Z*	2	4	4	4	8
ρ_calc_ [g cm^–3^]	1.390	1.334	1.282	1.274	1.380
μ [mm^–1^]	0.915	0.831	0.780	0.788	0.954
*F*(000)	484	632	600	1472	1120
Crystal size [mm^3^]	0.110 × 0.130 × 0.160	0.090 × 0.130 × 0.150	0.020 × 0.050 × 0.060	0.090 × 0.200 × 0.210	0.130 × 0.180 × 0.190
Crystal color	colorless	colorless	colorless	colorless	colorless
Crystal shape	block	fragment	flake	plate	block
Radiation	Cu*K*_α_ (λ = 1.54178 Å)	Cu*K*_α_ (λ = 1.54178 Å)	Cu*K*_α_ (λ = 1.54178 Å)	Cu*K*_α_ (λ = 1.54178 Å)	Cu*K*_α_ (λ = 1.54178 Å)
2θ range [°]	12.63 to 160.21 (0.78 Å)	9.92 to 159.77 (0.78 Å)	8.06 to 160.16 (0.78 Å)	5.30 to 160.24 (0.78 Å)	6.86 to 159.88 (0.78 Å)
Index ranges	–11 ≤ *h* ≤ 13	–11 ≤ *h* ≤ 11	–14 ≤ *h* ≤ 14	–8 ≤ *h* ≤ 7	–13 ≤ *h* ≤ 12
	–10 ≤ *k* ≤ 10	–17 ≤ *k* ≤ 17	–8 ≤ *k* ≤ 8	–25 ≤ *k* ≤ 29	–11 ≤ *k* ≤ 12
	–16 ≤ *l* ≤ 15	–15 ≤ *l* ≤ 14	–25 ≤ *l* ≤ 19	–29 ≤ *l* ≤ 30	–32 ≤ *l* ≤ 28
Reflections collected	22779	17320	12981	45797	52728
Independent reflections	2307	3196	3135	14244	2730
	*R*_int_ = 0.0416	*R*_int_ = 0.0865	*R*_int_ = 0.0599	*R*_int_ = 0.0551	*R*_int_ = 0.0293
	*R*_sigma_ = 0.0323	*R*_sigma_ = 0.0842	*R*_sigma_ = 0.0576	*R*_sigma_ = 0.0560	*R*_sigma_ = 0.0143
Completeness to θ = 67.679°	99.0%	100.0%	99.7%	99.9%	100.0%
Data/Restraints/Parameters	2307/0/153	3196/0/195	3135/318/256	14244/19/894	2730/0/184
Absorption correction *T*_min_/*T*_max_ (method)	0.6642/0.7543(multiscan)	0.5662/0.7543 (multiscan)	0.6084/0.7543 (multiscan)	0.6093/0.7543 (multiscan)	0.6866/0.7543 (multiscan)
Goodness-of-fit on *F* ^2^	1.222	1.060	1.041	1.051	1.048
Final *R* indexes [*I* ≥ 2σ(*I*)]	*R*_1_ = 0.0473	*R*_1_ = 0.0549	*R*_1_ = 0.0604	*R*_1_ = 0.0473	*R*_1_ = 0.0301
	w*R*_2_ = 0.1087	w*R*_2_ = 0.1441	w*R*_2_ = 0.1648	w*R*_2_ = 0.1259	w*R*_2_ = 0.0803
Final *R* indexes [all data]	*R*_1_ = 0.0494	*R*_1_ = 0.0716	*R*_1_ = 0.0764	*R*_1_ = 0.0485	*R*_1_ = 0.0307
	w*R*_2_ = 0.1092	w*R*_2_ = 0.1585	w*R*_2_ = 0.1743	w*R*_2_ = 0.1268	w*R*_2_ = 0.0807
Largest peak/hole [e Å^–3^]	0.32/–0.20	0.28/–0.21	0.30/–0.28	0.38/–0.23	0.31/–0.19

	TXA-MLE-H_2_O	TXA-CTR	TXA-ADP
CCDC number	**2435659**	**2435624**	**2435648**
Empirical formula	C_12_H_21_NO_7_	C_14_H_23_NO_9_	C_22_H_40_N_2_O_8_
Moiety formula	C_8_H_16_NO_2_·C_4_H_3_O_4_·H_2_O	C_6_H_7_O_7_·C_8_H_16_NO_2_	2(C_8_H_16_NO_2_)·C_6_H_8_O_4_
Formula weight	291.30	349.33	460.56
Temperature [K]	150(2)	150(2)	150(2)
Crystal system	monoclinic	triclinic	monoclinic
Space group (number)	*P*2_1_/*c* (14)	*P*1̅ (2)	*P*2_1_/*n* (14)
*a* [Å]	20.220(2)	6.1653(2)	11.2743(12)
*b* [Å]	6.9001(6)	11.1121(3)	5.6403(7)
*c* [Å]	10.4494(7)	12.4264(4)	19.231(2)
α [°]	90	69.7695(9)	90
β [°]	93.759(9)	85.8749(10)	100.842(8)
γ [°]	90	80.3449(10)	90
Volume [Å^3^]	1454.8(2)	787.40(4)	1201.1(2)
*Z*	4	2	2
ρ_calc_ [g cm^–3^]	1.330	1.473	1.273
μ [mm^–1^]	0.933	1.062	0.796
*F*(000)	624	372	500
Crystal size [mm^3^]	0.110 × 0.220 × 0.270	0.140 × 0.230 × 0.260	0.030 × 0.140 × 0.190
Crystal color	colorless	colorless	colorless
Crystal shape	plate	fragment	plate
Radiation	Cu*K*_α_ (λ = 1.54178 Å)	Cu*K*_α_ (λ = 1.54178 Å)	Cu*K*_α_ (λ = 1.54178 Å)
2θ range [°]	4.38 to 160.16 (0.78 Å)	7.58 to 160.32 (0.78 Å)	8.46 to 159.85 (0.78 Å)
Index ranges	–25 ≤ *h* ≤ 23	–7 ≤ *h* ≤ 7	–14 ≤ *h* ≤ 13
	–8 ≤ *k* ≤ 8	–14 ≤ *k* ≤ 14	–7 ≤ *k* ≤ 6
	–12 ≤ *l* ≤ 13	–15 ≤ *l* ≤ 15	–20 ≤ *l* ≤ 24
Reflections collected	20854	12844	10133
Independent reflections	3132	3344	2564
	*R*_int_ = 0.0638	*R*_int_ = 0.0449	*R*_int_ = 0.0366
	*R*_sigma_ = 0.0513	*R*_sigma_ = 0.0421	*R*_sigma_ = 0.0328
Completeness to θ = 67.679°	99.8%	99.8%	99.9%
Data/Restraints/Parameters	3132/0/195	3344/0/238	2564/0/157
Absorption correction *T*_min_/*T*_max_ (method)	0.6662/0.7543 (multiscan)	0.6362/0.7543 (multiscan)	0.6324/0.7543 (multiscan)
Goodness-of-fit on *F* ^2^	1.041	1.073	1.072
Final *R* indexes [*I* ≥ 2σ(*I*)]	*R*_1_ = 0.0339	*R*_1_ = 0.0328	*R*_1_ = 0.0349
	w*R*_2_ = 0.0868	w*R*_2_ = 0.0877	w*R*_2_ = 0.0950
Final *R* indexes [all data]	*R*_1_ = 0.0593	*R*_1_ = 0.0399	*R*_1_ = 0.0392
	w*R*_2_ = 0.0904	w*R*_2_ = 0.0894	w*R*_2_ = 0.0973
Largest peak/hole [e Å^–3^]	0.23/–0.18	0.35/–0.25	0.29/–0.21

a Computer programs: APEX4, APEX5, and SAINT (Bruker, 2022, 2023); SHELXT (Sheldrick, 2015a); SHELXL2019/2 (Sheldrick, 2015b); ShelXle (Hübschle et al.).^36,38,40–45^

## 3 Results and Discussion

### 3.1 Supramolecular Screening

The tendency of TXA to form multicomponent binary systems with (di)­carboxylic acids through noncovalent and ionic interactions was investigated by spectroscopic methods. Synthon design for the TXA multicomponent systems was based on interactions between the carboxylate group (−COO^–^) and ammonium group (−NH_3_^+^) of TXA and the −COOH of the coformers. The selection of coformers for TXA was driven by structural mimicry, using Mercury’s molecular complementarity tool^47^ to assess their potential for cocrystal or salt formation. Likewise, the possibility of cocrystal or salt formation from the crystallization experiments was estimated using the p*K*_a_ rule, where Δp*K*_a_ = p*K*_a_(base) – p*K*_a_(acid). According to this rule, a Δp*K*_a_ ≥ 3 favors salt formation with proton transfer, while a Δp*K*_a_ ≤ 1 favors cocrystal formation stabilized by a medium to strong hydrogen bond. A Δp*K*_a_ value between 1 and 3 may result in either a salt or a cocrystal. In the solid state, TXA exists as a zwitterion, with a p*K*_a_ value of 10.4 for the ammonium cation (NH_3_^+^),^48^ and 4.3 for the conjugate acid of the carboxylate anion (COO^–^). For cocrystal formation, TXA acts as a base, and the Δp*K*_a_ calculation requires the p*K*_a_ of the latter, which is 4.3, and needs to be used. The coformers (mostly carboxylic acids) exhibit pKa1 values between 1.0 and 4.20 and pKa2 values above 4.70. The propensity of salt/cocrystal formation with TXA acting as a base (i.e., the carboxylate anion acting as a proton acceptor, p*K*_a(COOH)_ = 4.3) was calculated and is summarized in Table 2. Compared to salts, only a few cocrystals of TXA have been reported. Nechipadappu et al. reported TXA with phenolic coformers such as catechol, resorcinol, hydroquinone, pyrogallol, and phloroglucinol, and with gallic and crotonic acids in a separate article.^15,22^ MacGillivray et al. reported an ionic cocrystal of TXA with 2,4-pyridinedicarboxylic acid.^24^ Herein, the Δp*K*_a_ rule served as a predictive guideline rather than a deterministic tool, recognizing its inherent limitations in borderline cases (Δp*K*_a_ 1–3) where crystal packing forces can override solution-phase predictions. Also, the absence of pH adjustment was a deliberate choice to evaluate the intrinsic propensity of TXA and coformers to form multicomponent solids under simple, reproducible conditions that mimic standard pharmaceutical cocrystal screening workflows.

**Table 2:** Coformers p*K*_a_ and Δp*K*_a_ Values and Predicted Adduct Formed^a^

Acid (Coformer) p*K*_a_1, (p*K*_a_2)	Δ(p*K*_a_1	Δ(p*K*_a_2	Predicted	Observed
Malic (MA) 3.40, (5.11)	+0.90	–0.81	Cocrystal	2:1 Salt
Malonic (MAL) 2.83, (5.69)	+1.47	–1.39	1:1 Salt or Cocrystal	1:1 Salt
Picolinic (PIC) 1.08, (5.20)	+3.22	–0.9	1:1 Salt	1:1 Salt
Maleic (MLE) 1.92, (6.23)	+2.38	–1.93	1:1 Salt or Cocrystal	1:1 Salt hydrate
Adipic (ADP) 4.14, (5.41)	–0.11	–1.11	Cocrystal	2:1 Salt
Acetylsalicylic (ASA) 3.49	+0.81	_	Cocrystal	1:1 Salt
Acetaminophen (APAP) 9.38	–5.08	_	Cocrystal	3:1 Cocrystal hydrate
Citric acid (CTR) 3.13, (4.76)	+1.17	–0.46	1:1 Salt or Cocrystal	1:1 Salt

a Δp*K*_a_ = p*K*_a_(conjugate acid of base)–p*K*_a_(acid). p*K*_a_(TXA) (conjugate acid of COO^–^): 4.3.

The coformers are carboxylic acids, which cannot be further protonated, and can only act as proton donors (acids), with the carboxylate anion of the TXA zwitterions acting as the base. The p*K*_a_ differences between the coformers’ COOH group and the conjugate acid of the TXA COO- carboxylates are small, and for most pairs the expected outcome would be cocrystal formation, possibly with partial proton transfer. However, some p*K*_a_ differences are sufficiently large that proton transfer onto TXA might occur. Predictions are that stronger acids (malonic, picolinic, maleic, citric) might lead to salt formation (proton transfer to the carboxylate anion of TXA). For all of them, the coformers with two acidic protons are expected to retain the second proton. For the weaker acid coformers (malic, adipic, acetylsalicylic, and especially acetaminophen), the most likely outcome was predicted to be cocrystal formation. Interestingly, this is not what has been observed (see crystal structure determinations and discussions below). Only the weakest acid in the series, acetaminophen, led to the formation of a cocrystal. For all other coformers, salt formation with complete protonation of TXA was observed. For two of the diprotic acids, even double deprotonation and formation of a 2:1 salt was observed (malic and adipic acids). A possible rationalization of this observation is that, beyond the intrinsic p*K*_a_ stabilization, the crystal packing environment and the formation of strong, charge-assisted N^+^–H···^–^O hydrogen bonds in the solid state contribute to stabilization of full and even double proton transfer (salt formation).

The crystalline architecture of TXA with carboxylic acid coformers is primarily directed by the thermodynamic drive to form a robust ionic heterosynthon. This preference is driven not only by a significant Δp*K*_a_ but, critically, by the superior lattice energy gained from strong, charge-assisted hydrogen bonds (∼100–150 kJ/mol). In the starting neutral zwitterionic TXA molecule, proton transfer from the carboxylic acid −COOH to the amino functionality ···NH_2_– has already occurred. The anion and cation are, however, spatially separated within the molecule, being on opposite sides of the molecule and not able to directly interact with each other. Intramolecularly, they are not able to form a charge-stabilized −NH_3_^+^···^–^OOC– pair. In the solid state, molecules can interact with neighboring molecules in an intermolecular fashion. In the structure of TXA itself, ammonium and carboxylate groups from adjacent molecules in the lattice form charge assisted hydrogen bonds in which each −NH_3_^+^ cation is bonded to three O atoms of carboxylate anions, and each COO^–^ anion in turn binds to three ammonium cations. One of the carboxylate O atoms is H-bonded to two −NH_3_^+^ groups, the other O atom to one −NH_3_^+^, leading to infinite 1D chains parallel to the *b*-axis of TXA’s orthorhombic lattice. The double ammonium H-bound O atom has a substantially longer C–O bond length than the single-bond one (1.267 vs 1.247), suggesting that one of the O atoms carries the bulk of the negative charge, while the other is closer to a double bonded keto C=O oxygen atom. This indicates that two of the −NH_3_^+^···^–^OOC bonds are fully charge-assisted −NH_3_^+^···^–^O–C interactions (those to the charge carrying O atom), while the other is closer to an NH_3_^+^···O=C interaction. It thus appears that the spatial separation of the −NH_3_^+^ and ^–^OOC functionalities in the TXA molecule limits how effectively charge-assisted −NH_3_^+^···^–^OOC bonds can be formed in the solid state.

In the formulations with the coformers, many more arrangements of cations and anions are possible. Ammonium and carboxylate functionalities are in separate molecular entities, which allows them to interact in a much more efficient way in the solid state. This leads to a large number of hydrogen bonding interactions, with each H-bond donor bonded to more acceptor atoms and vice versa, and to a more efficient formation of charge-assisted −NH_3_^+^···^–^OOC bonds. In the 2:1 TXA-ADP salt, as an example, each ammonium ion is bonded to four oxygen atoms (from two ADP carboxylates and one TXA carboxylic acid group), the TXA COOH forms a strong H-bond to an ADP COO-, and each ADP COO- is bonded to two NH_3_^+^ and one COOH. The H-bonding network thus formed is much more intricate than in the TXA crystal structure, and a 3D hydrogen-bonded network is formed instead of a 1D-chain. H-bonding interactions, and especially charge-assisted interactions, play a much more dominant role in the structures of the coformers than in the structure of TXA alone. Similar observations are made for the other salts and cocrystals involving TXA, which will be discussed in more detail in the descriptions for each structure.

The presence of other acceptor functionalities in some of the coformers provides additional opportunities for energetic stabilization. While weaker homosynthons like −COOH···HOOC– (∼30–60 kJ/mol) and other noncovalent interactions (O–H···O, C–H···O, C–H···π) provide individually a much smaller stabilization, they are nonetheless an essential part of the stabilization. They generally tend to support the charge-assisted interactions and provide additional stabilization without weakening the dominant charge-assisted interaction. They thus provide additional stabilization that is not possible in the absence of the coformers and allow for the formation of intricate 3D H-bonding networks not possible for the zwitterionic structure of TXA itself.

Vibrational spectroscopy provides direct evidence of intermolecular interactions in TXA-coformer systems by probing functional group vibrations. The definitive spectral changes, specifically, the replacement of the coformer carboxylic acid C=O stretch by that of TXA, and of the TXA carboxylate symmetric/asymmetric stretches by those of the coformer, allow for a clear distinction between ionic −NH_3_^+^···^–^OOC– interactions in salts with H atom transfer, and in cocrystals where TXA retained its carboxylate H atom. Figure 3a shows Raman spectra and functional group positions for TXA taken from 200–1700 cm^–1^, with shaded regions 930–1200 cm^–1^ and 1450–1700 cm^–1^ representing areas for evidence of intermolecular interactions. The Raman spectra for TXA-ADP, TXA-CTR, and TXA-MLE, shown in Figure 3b–d, highlight differences in specific spectral ranges that confirm the presence of intermolecular interactions. The characteristic intense doublet peaks of TXA at 789 cm^–1^ and 1031 cm^–1^, assigned to ring deformation and C–N stretching, respectively, shift slightly and have lower intensity (because of dilution of TXA by the coformer). In Figure 3b (TXA-ADP), the differences at 1441 cm^–1^ and the broad peaks at 1538 cm^–1^ and 1644 cm^–1^ are evidence of intermolecular interaction via ionic COO^–^ stretching. Similar evidence of intermolecular interactions is observed in the Raman spectra of TXA-CTR and TXA-MLE in Figure 3c,d. Herein, major peak shifts and intensity variations between 600–1700 cm^–1^, including C=O and C–N (def) at 706 cm^–1^ and 1031 cm^–1^, respectively, and C–N stretching at 1450 cm^–1^ are monitored to evidence intermolecular interactions. Additionally, TXA multicomponent forms (Figure 3b–d) exhibit further ionic interaction signatures, notably protonated COO^–^ (*asym*) stretching at 1536 cm^–1^ and 1468 cm^–1^, and protonated COO^–^ (*sym*) stretching at 1468 cm^–1^. All the TXA/coformer combinations show evidence of intermolecular interactions. See SI for spectroscopy screening.

**Figure 3:**
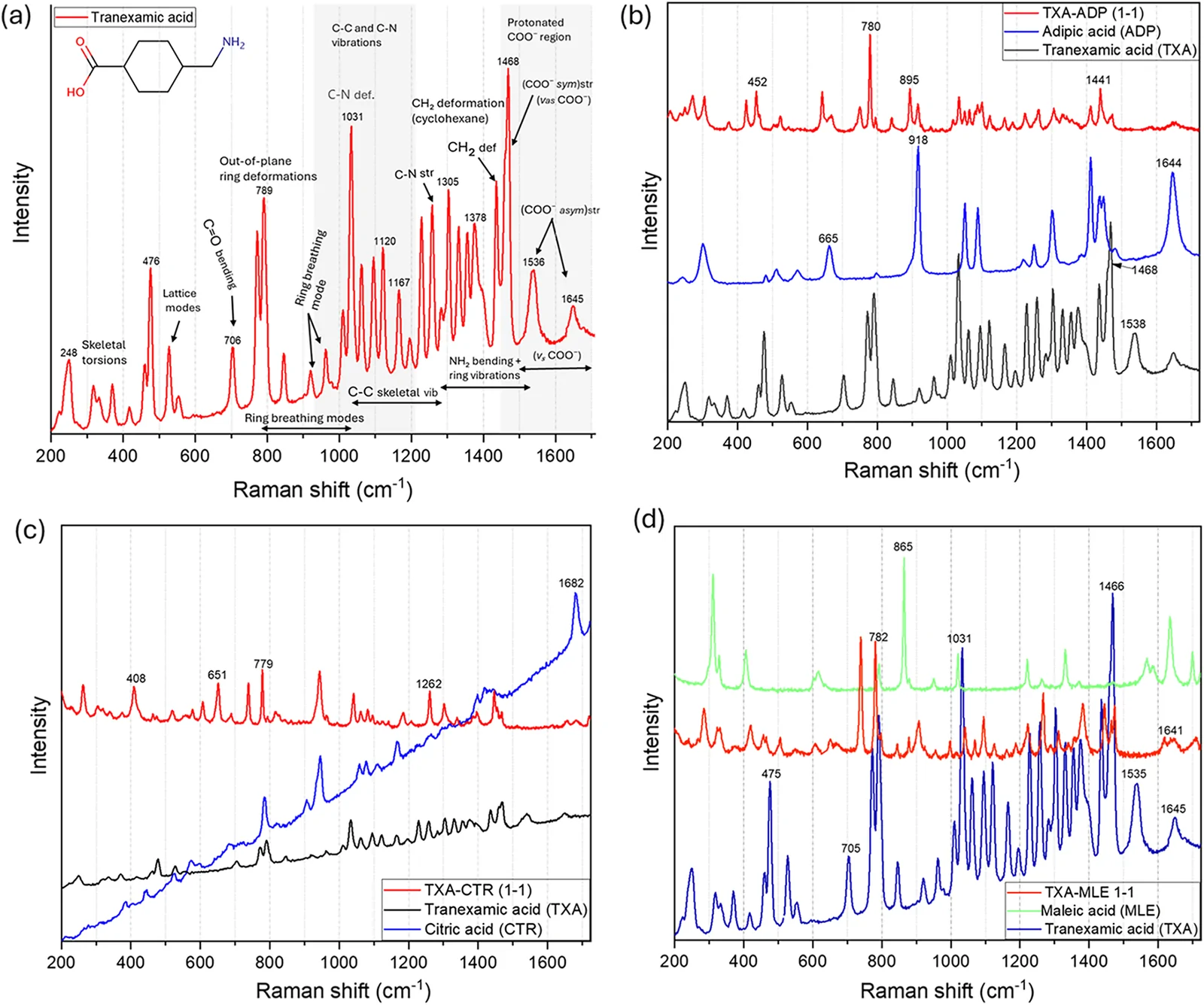
(a) Raman spectra of Tranexamic acid (TXA) showing functional group positions from 200–1720 cm^–1^, the spectra comparison using the low-wavenumber (<500 cm^–1^) and fingerprint region (500–1600 cm^–1^); spectra comparison for (b) TXA-ADP, (c) for TXA-CTR, and (d) TXA-MLE.

Using FT-Raman spectroscopy, the vibrational modes and functional group assignments of TXA are presented in Figure 4a. The high-frequency region (2800–3300 cm^–1^, insets in Figure 4) provides critical evidence for H-bonding interactions involving N–H and O–H donors. In all TXA salt forms (e.g., TXA–CTR, TXA–ADP, TXA–MLE), a broad peak (about 3000 cm^–1^) is observed, assigned to overlapping N^+^–H stretching vibrations from the protonated ammonium group (NH_3_^+^) of TXA and O–H stretching of the coformers. This broadening is characteristic of strong, charge-assisted hydrogen bonds (N^+^–H···^–^O and O–H···^–^O) that lower and widen the stretching frequencies. Specifically, the N^+^–H stretching bands in TXA salts appear as a broad envelope between 2800 and 3100 cm^–1^, which is consistent with literature reports for ammonium carboxylate salts.^26,49^ In contrast, the cocrystal hydrate TXA–APAP displays broader bands due to H-bonding of APAP, H_2_O, and TXA, consistent with the absence of strong ionic interactions.

**Figure 4:**
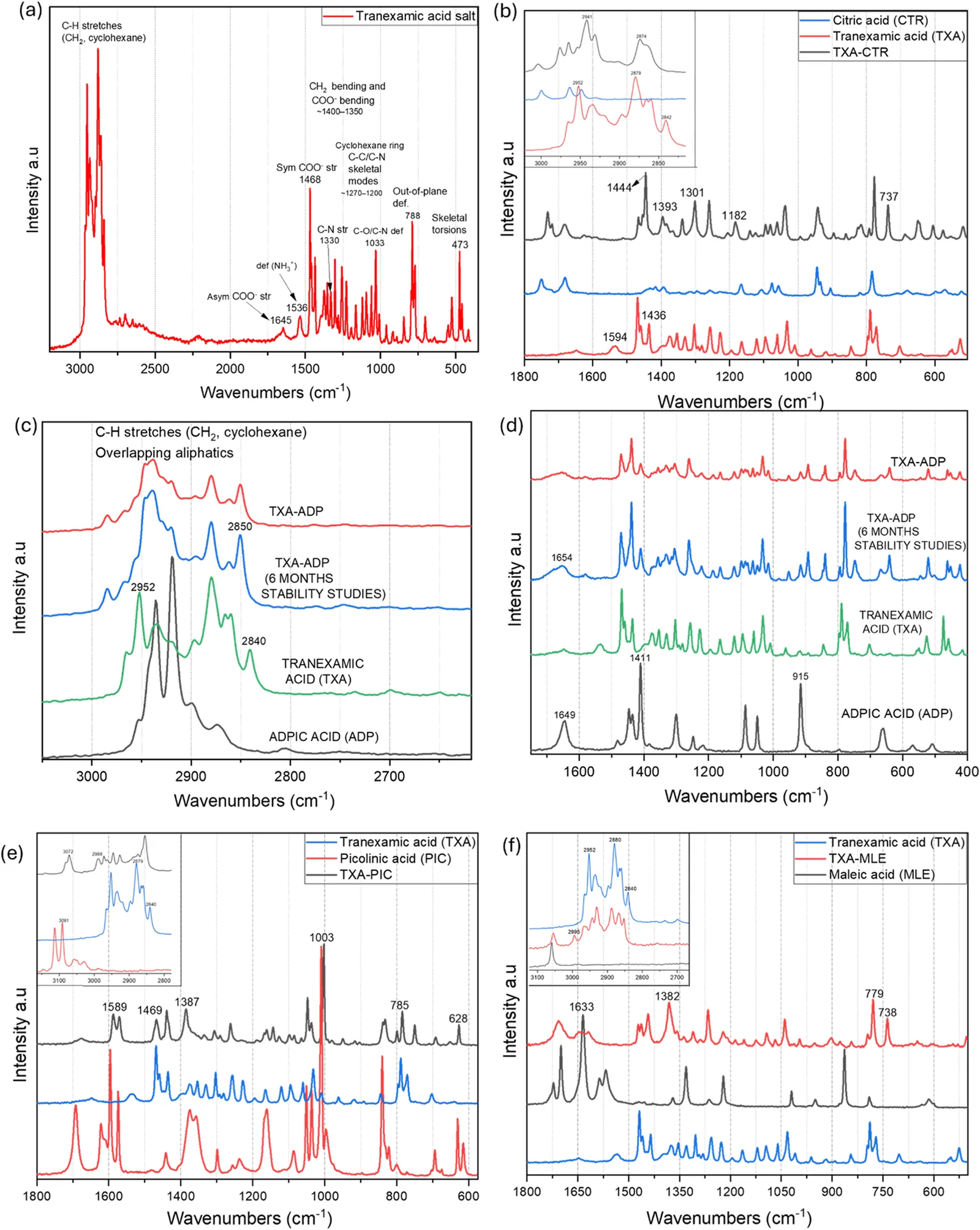
(a) FT-Raman spectra of tranexamic acid (TXA) in the range 300–3300 cm^–1^, highlighting functional group vibrations. Spectral comparisons in the high-wavenumber (>1500 cm^–1^) and fingerprint region (500–1600 cm^–1^) for: (b) TXA-CTR, (c, d) TXA-ADP, (e) TXA-PIC, and (f) TXA-MLE.

The COO^–^ (*asym*) stretching between 1550–1660 cm^–1^, COO^–^ (*asym*) stretching at 1468 cm^–1^, and NH_3_^+^ (def./bend) at ∼1540 cm^–1^ indicate protonation and evidence of ionic intermolecular interactions. Therefore, the COO^–^ (*bend*) and NH_3_^+^ (*def*) are observed for TXA-CTR at 1393 cm^–1^ and 1594 cm^–1^, respectively (Figure 4b). The cyclohexane ring vibration between 3200–2700 cm^–1^ (Figure 4c) and the broader NH_3_^+^ (*def*) peak at 1540 cm^–1^ for TXA-ADP (Figure 4d) indicate intermolecular interactions. The NH_3_^+^ (def) at 1589 cm^–1^, COO^–^ (*bend*) at 1387 cm^–1^, and C–N­(def) at 1003 cm^–1^ for TXA-PIC (Figure 4e) are evidence for structural modification, while the formation of TXA-MLE (Figure 4f) is supported by a peak assigned to C=O (*str*) at 1708 cm^–1^, COO^–^ (str) at 1646 cm^–1^, NH_2_ (*bend*) at 1621 cm^–1^, doublet peak assigned to COO^–^ *sym* stretching at 1468 cm^–1^, and presence of new peaks at 1311 cm^–1^, 737 cm^–1^ and 503 cm^–1^. The observed vibrational shifts are in good agreement with DFT-calculated spectra and with experimental data for related amino acid salts (e.g., glycine salts).^26^ See SI for complete vibrational spectroscopic assignments and screening between TXA and coformers.

### 3.2 Single Crystal XRD Analysis and Structural Descriptions

ORTEP representations of the asymmetric units for all the novel TXA multicomponent forms are presented in Figure 2, with corresponding crystallographic data and refinement details provided in Table 1A,B. Additional information on the crystallographic parameters and refinement details are available in the SI section. The Δp*K*_a_ prediction in **Figure 3**.**1** (Table 2) underestimates the likelihood of salt formation (proton transfer from the carboxylic acid COOH of the coformer to COO^–^ of TXA) in the reactions between TXA and carboxylic acids. Here, TXA is not much of an acid (p*K*_a_ of its ammonium cation is 10.4) and will not contribute to the protonation of the COO- of the acid coformer. Instead, the COO^–^ of TXA is protonated and functions as a neutral but strongly H-bonded component in the TXA multicomponent forms, while the carboxylic acid COOH of the coformer is (in most compounds) deprotonated and functions as the anionic component during salt formation. The stabilization of carboxylate bonds (NH_3_^+^···^–^OOC) by ammonium via charge-assisted N^+^–H···^–^O hydrogen bonding is the strongest interaction in the TXA multicomponent forms, with charge-transfer energies estimated at 40–60 kJ/mol per interaction. Hydrogen bonds involving N^+^–H···O^–^, O–H···O^–^, N–H···O, and O–H···O interactions are observed for the TXA multicomponents except TXA-APAP. The presence of weak C–H···O and C–H···π interactions also stabilize the structures.

#### 3.2.1 TXA-MLE Monohydrate (1:1)

This salt hydrate crystallizes in the monoclinic space group *P2*_1_/*c* with one TXA cation, one MLE anion, and one water molecule in the asymmetric unit (Figure 2f). As discussed above, complete proton transfer from the carboxylic acid coformer (MLE) to the carboxylate group of TXA was confirmed, resulting in a TXA^+^ cation (ammonium, NH_3_^+^) and an MLE^–^ anion (carboxylate, COO^–^). The ammonium group of TXA forms a strong, charge-assisted bifurcated N^+^–H···O^–^ H-bond with the carboxylate group of MLE via a bifurcated N^+^–H···O^–^ H-bond (N1–H1A···O4, 2.8564(11) Å). This interaction is part of a robust, multidonor hydrogen bond network that stabilizes the salt structure. The same ammonium group also donates a separate N^+^–H···O hydrogen bond to the water molecule (N1–H1C···O7, 2.7726(15) Å), as presented in Figure 5b,c. The bifurcated N^+^–H···O^–^ interaction exhibits asymmetric d­(H···O) distances: N1–H1A···O4/1.96 Å and N1–H1B···O5/1.87 Å, characteristic of a three-centered H-bond. The anionic COO^–^ group is further stabilized by homosynthon dimer formation via strong O–H···O^–^ hydrogen bonds involving the water molecule and TXA’s hydroxyl group (O1–H1···O2, 2.6495(11) Å; O4–H4···O6, 2.4190(11) Å), generating a *R*_4_^4^(22) ring motif as shown in Figure 5a. This motif stabilizes crystal packing and plays a key role in defining the supramolecular architecture. Figure 5b–c shows the water molecules bridging components via H-bonds, including with a MLE molecule and via O–H**···**O^–^ bonds (O7**–**H7*C* **···**O5, 2.777(11) Å), and TXA molecules via N^+^–H···O bond (N1–H1C···O7, 2.7726(15) Å) and C–H···O interactions such as C8–H8A···O7, 3.5617(15) Å. The structure is further stabilized by various weak interactions, including intramolecular O–H···O hydrogen bonds in MLE^–^ (O4–H4···O6, 2.4190(11) Å) and C–H···O interactions such as C10–H10···O4 (2.560 Å) and C11–H11···O6 (2.510 Å). MLE^–^ anions and water molecules form a *R*_4_^4^(18) ring system (Figure 5b–c). The 2D layer, sustained by N^+^–H···O^–^, O–H···O^–^ and O–H···O interactions, are interconnected into a 3D supramolecular structure via additional weak C–H···O interactions; C2–H2···O1 (3.3343 Å), C8–H8A···O7 (3.5617 Å), C10–H10···O4 (3.4290 Å), and C11–H11···O6 (3.3088 Å). Figure 5d,e shows the crystal packings and interaction chains within the structure, while the hydrogen-bond geometry of TXA-MLE is shown in Table 3. Nechipadappu et al. also reported the TXA-MLE hydrate structure with a few property differences.^22^

**Figure 5:**
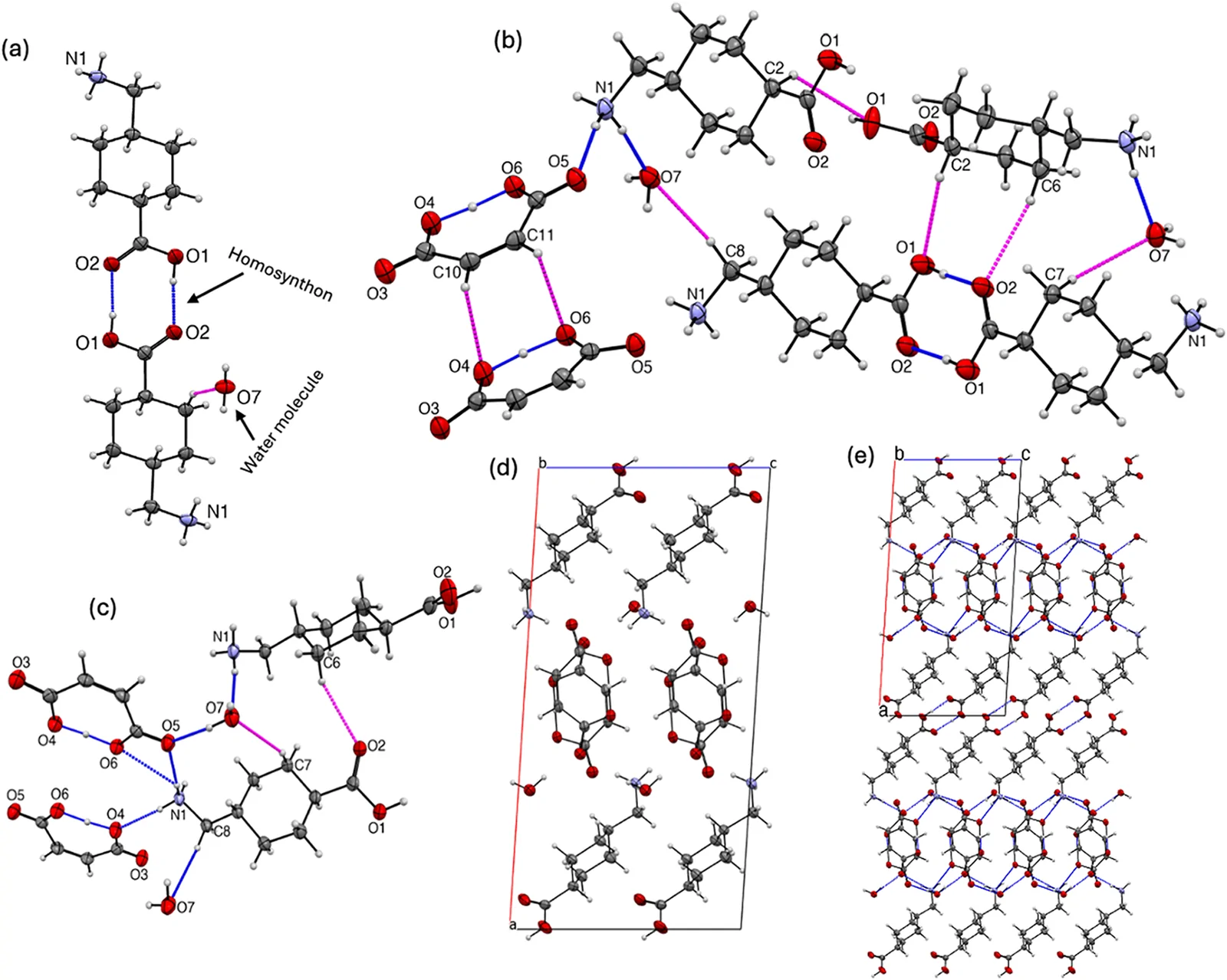
Hydrogen bonding and packing in TXA–MLE monohydrate: (a) TXA homosynthon dimer; (b, c) Strong N^+^–H···O^–^ and O–H···O^–^ hydrogen bonding (blue line) and C–H···O contact (magenta); (d) crystalline packing; and (e) infinite chain formation. C/H labels omitted for clarity. The probability of ellipsoids is at a 50% level for all the atoms.

**Table 3:** Hydrogen-Bond Geometry (Å) for the TXA-MLE Monohydrate Salt

D–H···A [Å]	d(D–H) [Å]	d(H···A) [Å]	d(D···A) [Å]	<(DHA) [°]	Symmetry transformations
N1–H1A···O4	0.91	1.96	2.8564(11)	168.8	1 – *X*, –0.5 + *Y*, 0.5 – *Z*
N1–H1B···O5	0.91	1.95	2.8389(13)	163.5	+*X*, 1.5 – *Y*, 0.5 + *Z*
N1–H1C···O7	0.91	1.87	2.7726(15)	173.1	
O1–H1···O2	0.88(2)	1.77(2)	2.6495(11)	173(2)	–*X*, 1 – *Y*, 2 – *Z*
O4–H4···O6	1.18(2)	1.24(2)	2.4190(11)	175(2)	
O7–H7C···O5	0.870(19)	1.944(19)	2.7777(11)	160.0(19)	
O7–H7D···O3	0.92(2)	1.84(2)	2.7550(13)	172.0(16)	1 – *X*, 2 – *Y*, 1 – *Z*
C2–H2···O1	1.00	2.66	3.3343(14)	125.2	–*X*, 0.5 + *Y*, 1.5 – *Z*
C8–H8A···O7	0.99	2.59	3.5617(15)	167.9	+*X*, –1 + *Y*, +*Z*
C10–H10···O4	0.95	2.56	3.4290(13)	152.9	+*X*, 1.5 – *Y*, 0.5 + *Z*

#### 3.2.2 TXA-MA (2:1) Salt

The crystal was solved in the monoclinic crystal system with the space group *P2*_*1*_/*c*, with two TXA cations and one malate dianion (MA^2–^) in its asymmetric unit (Figure 2d), reflecting a 2:1 stoichiometry. MA, being a diprotic acid, underwent complete double deprotonation. The hydroxyl group of the malate dianion is disordered in the solid state (the malate anion is located on a crystallographic inversion center). The disorder does not disrupt or affect the H-bonding interactions within the overall structure and does not override evidence for salt formation as structure parameters align with proton transfer from the malic acid COOH to the TXA COO^–^ (see Table 1). The protonated ammonium groups (−NH_3_^+^) of the two symmetry equivalent TXA cations form strong, charge-assisted hydrogen bonds with the carboxylate groups of the malate dianion (NH_3_^+^···^–^OOC), confirming salt formation. Table 4 provides direct evidence for the ionic character, showing short, charge-assisted N^+^–H···O^–^ bonds from both TXA cations to the malate dianion. For instance, the interactions include N1–H1A···O4 (2.8211(19) Å, 173.7°) and N1–H1B···O3 (2.835(2) Å, 153.0°). Figure 6a,b shows the extensive hydrogen bond network where the malate dianion, with four available oxygen acceptors, is integrated by multiple N^+^ −H···^–^O bonds from the two TXA cations; N1–H1C···O1 (3.0205(17) Å, 131.0°) and N1–H1C···O4 (2.9094(18) Å, 137.3°), forming a complex, multiacceptor motif that affirms the salt character. This creates a dense network of X–H···O bonds toward the anionic O atoms of the malate dianion, providing significant lattice stabilization. As illustrated in Figure 6a–c, the structure is stabilized by an extensive H-bond network, including ionic N^+^–H···O^–^ bonds, alongside the O–H···O bond (O5–H5O···O1, 2.743(3) Å), generating a 2D layer featuring a *R*_4_^4^(32) ring motif. The crystalline packing and chain layers shown in Figure 6e**-**g stack into an extended 3D framework driven by N^+^–H···O^–^, O–H···O, and weak C–H···O interactions, such as C8–H8B···O5, 3.163(3) Å. Table 4 shows the H-bond geometry, while other crystallographic parameters (refinement details) are available in the SI.

**Figure 6:**
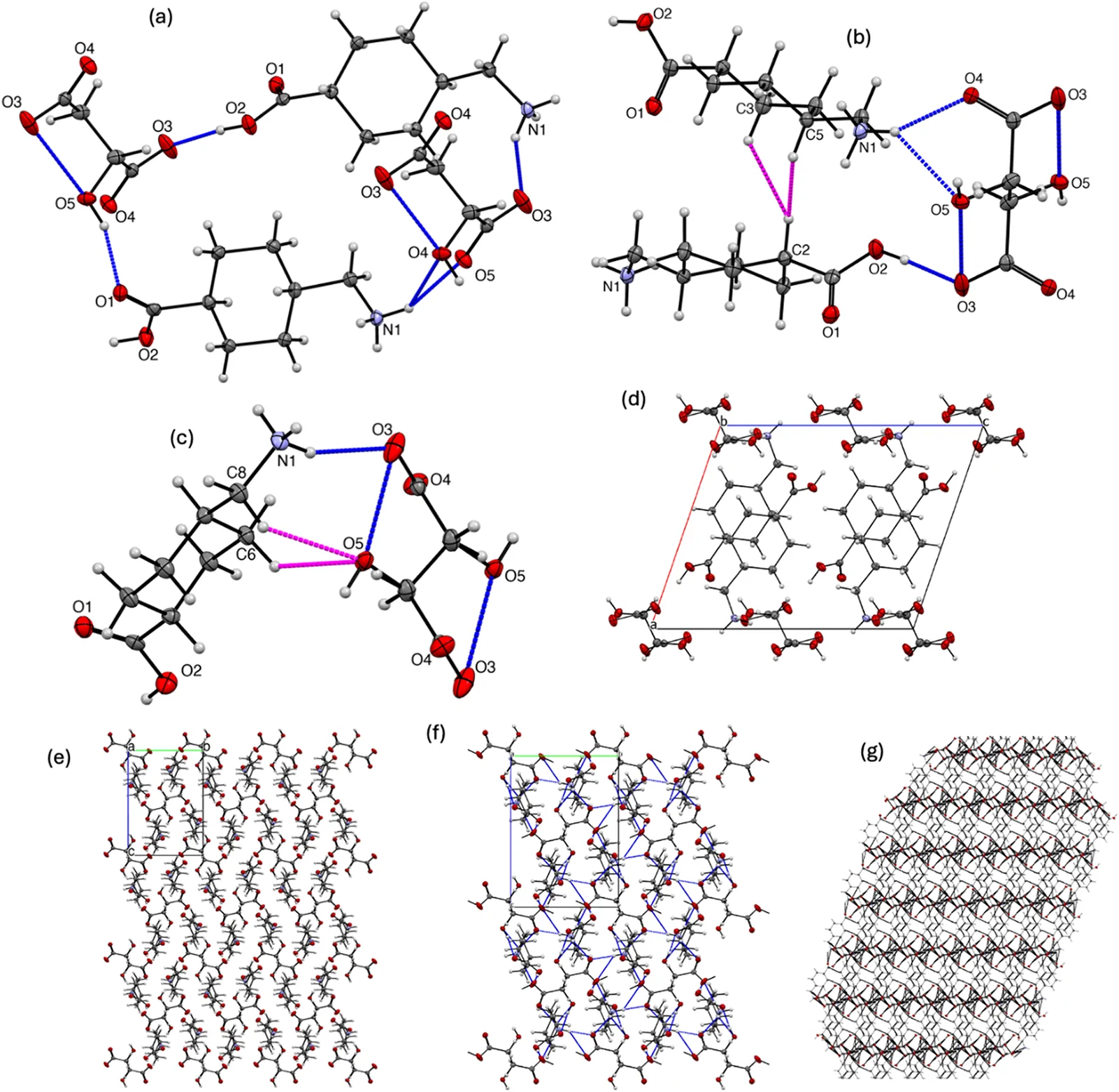
TXA-MA hydrogen bonding and packing: (a–c) Strong N ^+^ −H···O^–^ and O–H···O^–^ hydrogen bonds in (blue) and weak C–H···O bonds (magenta); (d–g) crystalline packing and infinite chain. Ellipsoids at 50% probability; labels on C and H atoms are omitted for clarity.

**Table 4:** Hydrogen-Bond Geometry (Å) for the TXA-MA Salt

D–H···A	d(D–H) [Å]	d(H···A) [Å]	d(D···A) [Å]	∠(DHA) [°]	Symmetry transformations
O2–H2O···O3	0.90(3)	1.72(3)	2.6094(17)	174(3)	
N1–H1A···O4	0.91	1.91	2.8211(19)	173.7	1 – *X*, −*Y*
N1–H1B···O3	0.91	1.99	2.835(2)	153.0	1 + *X*, +*Y*, +*Z*
N1–H1C···O1	0.91	2.34	3.0205(17)	131.0	1 + *X*, +*Y*, +*Z*
N1–H1C···O4	0.91	2.17	2.9094(18)	137.3	1 + *X*, 0.5 – *Y*
N1–H1C···O5	0.91	2.41	3.074(3)	130.2	1 + *X*, 0.5 – *Y*
C8–H8B···O5	0.99	2.45	3.163(3)	128.5	1 – *X*, 1 – *Y*, 1 – *Z*
O5–H5O···O1	0.89(5)	1.89(5)	2.743(3)	161(4)	+*X*, 0.5 – *Y*, 0.5 + Z

#### 3.2.3 TXA-ADP (2:1) Salt

A monoclinic crystal system with the space group *P2*_1_/*n*. The crystal structure confirms salt formation with two TXA cations and one adipate dianion (ADP^2–^) in the asymmetric unit (Figure 2h). The unit cell packing comprises eight TXA cations and four adipate dianions (Figure 7a). As observed for TXA-MA, the protonated ammonium groups of TXA (NH_3_^+^) act as key donors to the carboxylate groups of the adipate dianion (ADP^2–^) via N^+^–H···O^–^ bonds. Likewise, the characteristic bifurcated interaction from a single ammonium group to a carboxylate is observed, for example: N1–H1A···O3, 2.7794(12) Å and N1–H1···O4, 3.0916(13) Å, as illustrated in Figure 7b. The anionic adipate carboxylates are integrated into a robust, multidonor hydrogen bond network, primarily accepting strong, charge-assisted H-bonds from the protonated amine groups (NH_3_^+^) of the TXA cations via ionic N^+^–H···O^–^ H-bonds (N1–H1B···O4, 2.7553(13) Å). The extended network of ionic N^+^–H···O^–^ bonds between TXA and ADP dominates the structure to form *R*_4_^4^(22) ring system presented in Figure 7b. The cyclic arrays formed by these ionic hydrogen bonds (heterosynthon) N^+^–H···O^–^ create an extended chain network linking molecules into 2D layers, which were further stacked into a 3D network via weak C–H···O interactions. Figure 7e–g shows the crystal packings and interaction chains within the structure, and Table 5 presents the hydrogen-bond geometry. Other special structural details are available in the SI section.

**Figure 7:**
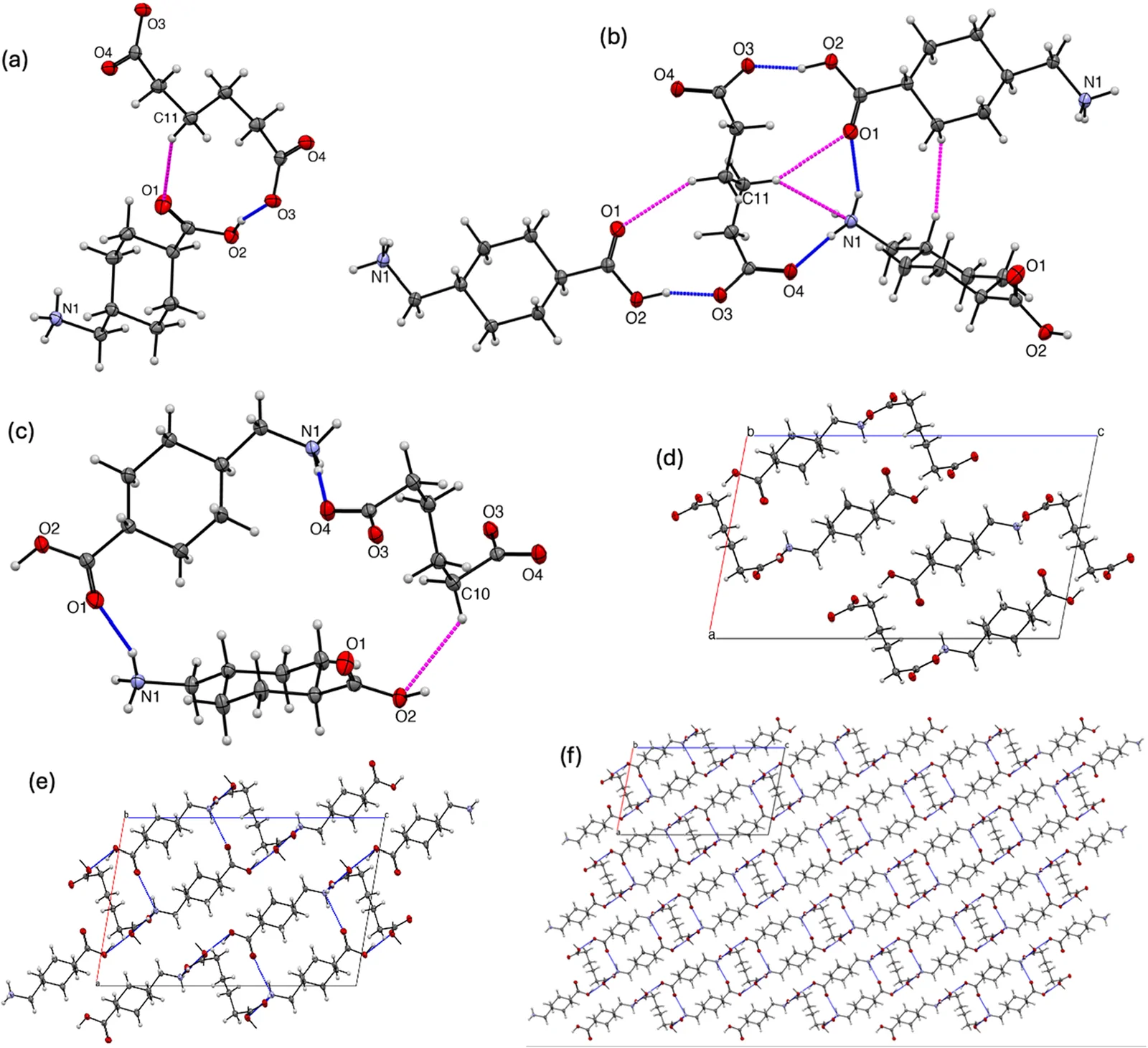
TXA–ADP hydrogen bonding and packing: (a) Strong H bond in N^+^–H···O^–^ (blue) and weak C–H···O (magenta); (b, c) bifurcated interaction via strong N^+^–H···O^–^ and O–H···O (blue), and weak C–H···O interactions (magenta); (d–f) crystalline packing and infinite chain. Ellipsoids at 50% probability; labels on C and H atoms are omitted for clarity.

**Table 5:** Hydrogen-Bond Geometry (Å) for the TXA-ADP Salt

D–H···A [Å]	d(D–H) [Å]	d(H···A) [Å]	d(D···A) [Å]	<(DHA) [°]	Symmetry transformations
O2–H2A···O3	0.946(16)	1.620(16)	2.5416(10)	163.4(15)	
N1–H1A···O3	0.926(16)	1.856(16)	2.7794(12)	175.2(14)	0.5 + *X*, 0.5 – *Y*, 0.5 + *Z*
N1–H1A···O4	0.926(16)	2.517(16)	3.0916(13)	120.5(12)	0.5 + *X*, 1.5 – *Y*, 0.5 + *Z*
N1–H1B···O4	0.911(17)	1.848(17)	2.7553(13)	173.1(15)	0.5 + *X*, 1.5 – *Y*, –0.5 + *Z*
N1–H1C···O1	0.914(16)	2.047(16)	2.9181(13)	159.0(13)	0.5 – *X*, –0.5 + *Y*, 0.5 – *Z*

#### 3.2.4 TXA-SAL (1:1) Salt

*The crystal* structure was solved in the monoclinic crystal system with the space group *P2*_1_/*n*. The asymmetric unit (Figure 2g) contains one TXA cation and one SAL anion (Figure 8d,e). The structure results from an *in situ* chemical transformation, where the intended acetylsalicylic acid (ASA) coformer underwent hydrolysis, cleaving the acetyl group to form salicylic acid (SAL) and acetic acid. Complete proton transfer from the carboxylic acid of SAL to the COO^–^ of TXA is confirmed, resulting in a TXA^+^ cation (ammonium, NH_3_^+^) and a salicylate anion (SAL^–^), evidenced by the C–O bond lengths of the salicylate carboxylate group (1.256(2) and 1.262(2) Å), which are characteristic of a delocalized COO^–^ anion, in contrast to a neutral −COOH. The protonated ammonium group of NH_3_^+^ forms a primary, charge-assisted ionic N^+^–H···O^–^ bond with a carboxylate oxygen of a symmetry-related SAL^–^ anion: N1–H1A···O3 (2.634(2) Å, 178.5°). This interaction is part of a bifurcated donation from the TXA (NH_3_^+^) to the deprotonated SAL carboxylate via N1–H1A···O3 (2.634(2) Å) and with a second contact at N1–H1A···O4 (3.007(2) Å), which contributes to the formation of a supramolecular homodimer *R*_4_^4^(22) ring, as shown in Figure 8a. A defining characteristic of the salicylate anion is the presence of an intramolecular O–H···O^–^ hydrogen bond (O5–H5A···O4, 2.539(2) Å), which chelates the anion and locks its conformation. The crystal packing is further stabilized by additional hydrogen bonds, including a neutral O1–H1···O3 (2.634(2) Å) interaction from the TXA carboxyl group and several N^+^–H···O^–^ H-bonds from the TXA ^+^ ammonium group, such as N1–H1B···O4 (2.764(2) Å) and N1–H1C···O2 (2.962(2) Å), as illustrated in Figure 8a–c. The extended network of these primary ionic N^+^–H···O^–^ and supporting O–H···O H-bonds creates 2D hydrogen-bonded layers. These layers stack along the *b*-axis via van der Waals interactions and weak C–H···O contacts, such as C15–H15···O2 (2.962 Å), to generate a layered 3D framework. The structure is further consolidated by π–π stacking (approximately 3.8 Å) between the aromatic rings of adjacent SAL^–^ anions. The hydrogen-bond geometry is detailed in Table 6, and the overall crystal packing and interaction networks are shown in Figure 8f,g.

**Figure 8:**
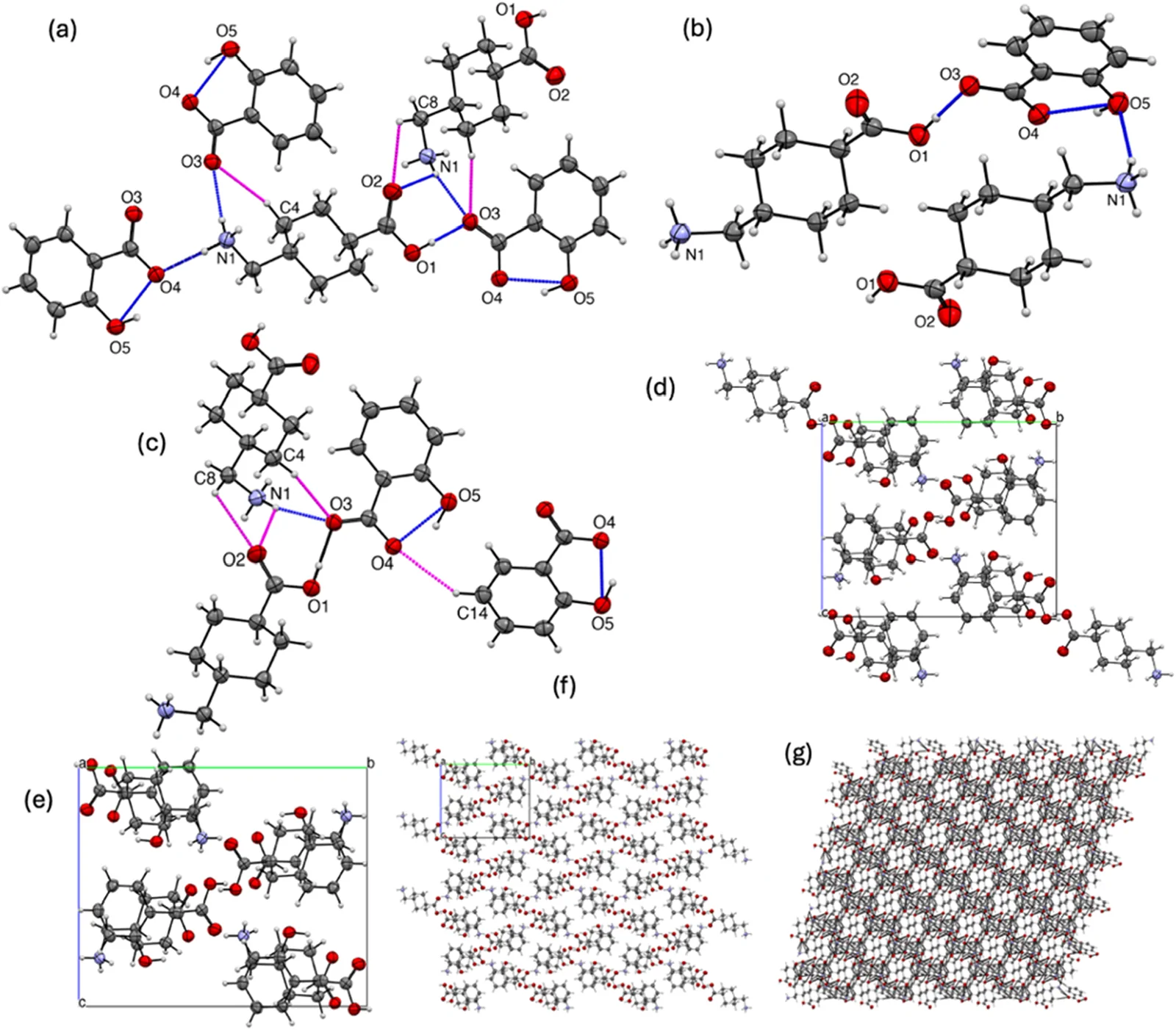
TXA–SAL hydrogen bonding and molecular packings: (a) Strong N–H···O (blue) and weak C–H···O interactions (magenta); (b, c) bifurcated interaction via strong N–H···O, O–H···O, and N–H···O (blue lines); (d–g) packing and infinite chain. Ellipsoids at 50% probability; labels on C and H atoms are omitted for clarity.

**Table 6:** Hydrogen-Bond Geometry (Å) for the TXA-SAL Salt

D–H···A [Å]	d(D–H) [Å]	d(H···A) [Å]	d(D···A) [Å]	<(DHA) [°]	Symmetry transformations
O1–H1···O3	0.90	1.73	2.634(2)	178.5	
O5–H5A···O4	0.94	1.71	2.539(2)	144.4	
N1–H1A···O1	0.91	2.56	3.007(2)	110.7	0.5 + *X*, 0.5 – *Y*, 0.5 + *Z*
N1–H1A···O5	0.91	2.28	3.155(2)	161.2	1 – *X*, 1 – *Y*, 1 – *Z*
N1–H1B···O4	0.91	1.87	2.764(2)	169.0	0.5 + *X*, 1.5 – *Y*, –0.5 + *Z*
N1–H1C···O2	0.91	2.42	2.962(2)	118.4	0.5 – *X*, –0.5 + *Y*, 0.5 – *Z*
N1–H1C···O3	0.91	2.08	2.898(2)	148.5	0.5 – *X*, –0.5 + *Y*, 0.5 – *Z*

#### 3.2.5 TXA-CTR (1:1) Salt

*The crystal* structure was solved in the triclinic crystal system with the space group *P*1̅ with one TXA molecule and one CTR molecule in its asymmetric unit (Figure 2b). The unit cell packing contains four TXA and four CTR molecules (Figure 9d and **e**). Complete proton transfer from citric acid (CTR) to COO^–^ of TXA is confirmed, resulting in a TXA^+^ cation (ammonium, NH_3_^+^) and a CTR^–^ anion (^−^OOC). The protonated ammonium group of TXA^+^ acts as a primary hydrogen bond donor to the carboxylate groups of CTR^–^ via strong, charge-assisted ionic N^+^–H···O^–^ bonds that establish key interactions that include N1–H1A···O9 (2.8186(12) Å, 166.4°) and N1–H1B···O5 (2.8270(12) Å, 167.1°), as presented in Figure 9a,b. The packing is further stabilized by a bifurcated interaction from the TXA^+^ ammonium group, which donates to both a carboxylate and a hydroxyl group of CTR^–^: N1–H1C···O3 (3.0645(13) Å) and N1–H1C···O9 (2.9033(13) Å) (Figure 9a). The anionic CTR^–^ is integrated into an extensive, multidonor hydrogen-bond network. This includes homodimeric interactions between carbonyl (C=O) and hydroxyl (−OH) groups of CTR^–^ molecules mediated by O–H···O bonds, such as O5–H5A···O4 (2.8954(12) Å), and the formation of diverse ring motifs like *R*_2_^2^(12) and *R*_4_^2^(8) ring systems, as illustrated in Figure 9b. Chains of N^+^ −H···O^–^ and O–H···O interactions, such as O1–H1···O8 (2.6248(11) Å, 168.8°), form corrugated 2D hydrogen-bonded sheets. An intramolecular hydrogen bond within the CTR^–^ anion, O3–H3···O8, (2.5988(11) Å), enhances its conformational rigidity and contributes to lattice cohesion. This 2D architecture is stacked into a 3D framework via van der Waals interactions and weak C–H···O contacts, such as C12–H12B···O7 (3.4383(15) Å). The robust packing and interaction chains are shown in Figure 9e. The hydrogen-bond geometry for the TXA-CTR salt is presented in Table 7, while other crystallographic refinement parameters are available in the SI section.

**Figure 9:**
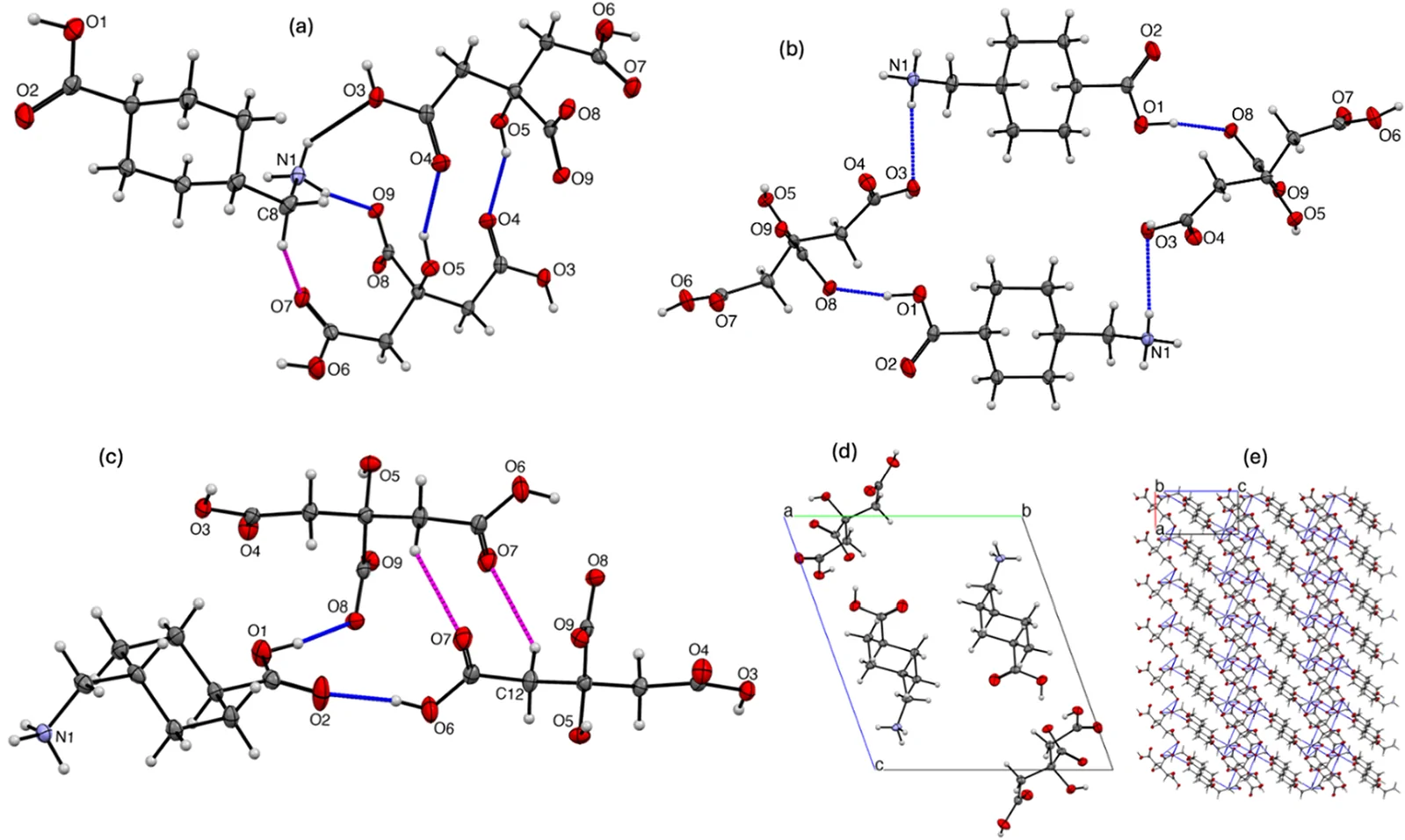
TXA–CTR hydrogen bonding and molecular packings: (a, b) N–H···O, O–H···O interactions (blue), and C–H···O (magenta); (c) O–H···O, and C–H···O (magenta); (d, e) packing and infinite chain. Ellipsoids at 50% probability; labels on C and H atoms are omitted for clarity.

**Table 7:** Hydrogen-Bond Geometry (Å) for the TXA-CTR Salt

D–H···A [Å]	d(D–H) [Å]	d(H···A) [Å]	d(D···A) [Å]	<(DHA) [°]	Symmetry transformations
O1–H1···O8	0.94(2)	1.70(2)	2.6248(11)	168.8(18)	
O3–H3···O8	0.879(18)	1.740(19)	2.5988(11)	164.8(16)	–1 + *X*, +*Y*, +*Z*
O3–H3···O9	0.879(18)	2.542(17)	2.9753(11)	111.2(13)	–1 + *X*, +*Y*, +*Z*
O5–H5A···O4	0.830(18)	2.295(17)	2.8954(12)	129.6(14)	1 – *X*, 2 – *Y*, 2 – *Z*
O5–H5A···O9	0.830(18)	2.076(17)	2.5890(11)	119.7(14)	
O6–H6···O2	0.86(2)	1.84(2)	2.6972(13)	172(2)	2 – *X*, 1 – *Y*, 2 – *Z*
N1–H1A···O9	0.947(17)	1.889(17)	2.8186(12)	166.4(15)	–1 + *X*, +*Y*, –1 + *Z*
N1–H1B···O5	0.922(17)	1.921(17)	2.8270(12)	167.1(15)	+*X*, +*Y*, –1 + *Z*
N1–H1C···O3	0.860(18)	2.411(17)	3.0645(13)	133.2(14)	–*X*, 2 – *Y*, 1 – *Z*
N1–H1C···O9	0.860(18)	2.236(17)	2.9033(13)	134.3(15)	1 – *X*, 2 – *Y*, 1 – *Z*
C8–H8A···O7	0.99	2.56	3.3000(14)	131.2	–1 + *X*, +*Y*, –1 + *Z*
C12–H12B···O7	0.99	2.56	3.4383(15)	147.9	2 – *X*, 1 – *Y*, 2 – *Z*

#### 3.2.6 TXA-APAP (3:1) Cocrystal Hydrate

The crystal structure was solved in the monoclinic crystal system with the space group *P2*_*1*_. The asymmetric unit comprises six molecules of TXA, two molecules of acetaminophen (APAP), and six water molecules (Figure 2e). The unit cell packing contains 12 TXA, four APAP, and 12 water molecules (Figure 10c). The presence of O–H···O hydrogen bonds involving water (e.g., O1_9, O2_9, O3_9, etc.) confirms the presence of a stoichiometric hydrate, as the H_2_O molecules are integral to the crystal lattice and their positions are well-defined. As shown in Figure 10a-b, the phenolic (−OH) of APAP forms strong hydrogen bonds with two water molecules via O–H···O: O3_7–H3A_7···O3_9 (2.596(4) Å) and O2_9–H2A_9···O3_7 (2.810(4) Å), and water–water interactions O–H···O: O2_9–H2B_9···O1_9 (2.835(4) Å) were observed. The structure is a cocrystal hydrate and not a salt, as there is no evidence of proton transfer from APAP to the carboxylate of TXA. This is confirmed by the presence of TXA in its zwitterionic form, with the nearly identical C–O bond lengths in the TXA carboxylate groups (e.g., O1_1–C1_1 = 1.262 Å and O2_1–C1_1 = 1.257 Å) being consistent with a −COO^–^ group, not a neutral −COOH group. The ammonium (NH_3_^+^) of the TXA molecule interacts with the carbonyl group (C=O) of another TXA molecule via half charge-assisted N–H^+^···O=C hydrogen bonds, for example: N1_1–H1A_1···O2_2 (2.736(4) Å) and N1_2–H1A_2···O2_3 (2.702(4) Å). This describes a head-to-tail TXA chain motif stabilized by strong H-bonds between the functional groups of adjacent TXA zwitterions. The crystal packing of this cocrystal hydrate is further stabilized by different H_2_O-mediated O–H···O interactions; O3_7–H3A_7···O3_9 (2.596(4) Å) and O1_9–H1A_9···O4_7 (2.685(4) Å), which bridge the structure into an extended 2D layer. The 2D layer is reinforced by weak C–H···O and C–H···π, contacts to form a 3D supramolecular architecture (Figure 10b). The crystalline packing and 3D network of N–H···O, O–H···O, C–H···π, and C–H···O interactions in the structure are presented in Figure 10c,d, with the H-bond geometry in Table 8. See SI for the complete H-bond table and other crystallographic refinement parameters.

**Figure 10:**
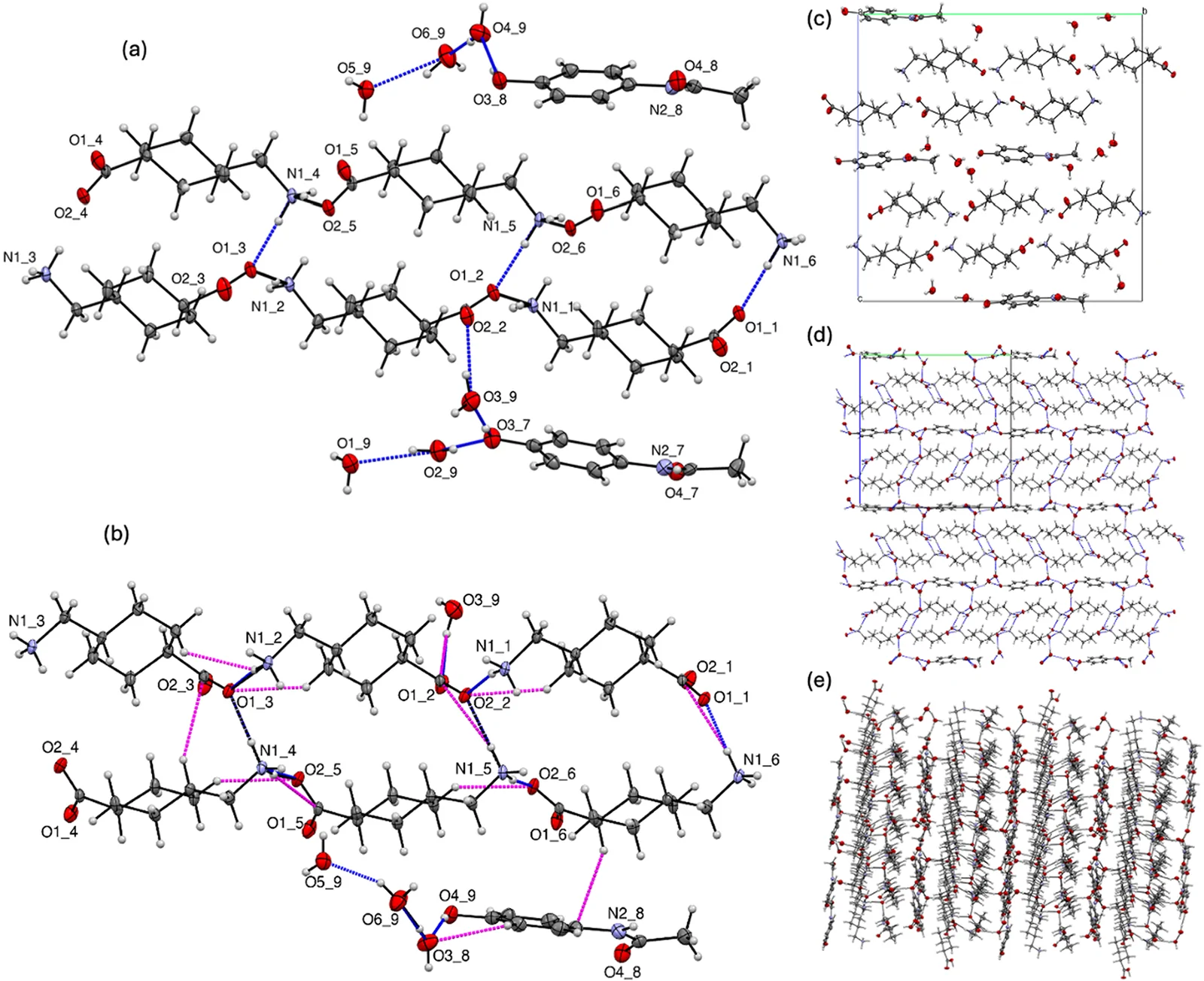
TXA–APAP hydrate hydrogen bonding and molecular packings: (a) N–H···O and O–H···O interactions (blue); (b) weak C–H···O contacts (magenta); (c–e) packing and infinite chain. Ellipsoids at 50% probability; labels on C and H atoms in the structures are omitted for clarity.

**Table 8:** Hydrogen Bond Geometry for TXA-APAP

D–H···A [Å]	d(D–H) [Å]	d(H···A) [Å]	d(D···A) [Å]	<(DHA) [°]	Symmetry transformations
N1_1–H1A_1···O2_2	0.91	1.83	2.736(4)	170.3	–1 + *X*, +*Y*, +*Z*
N1_1–H1B_1···O2_6	0.91	1.86	2.758(4)	170.4	–1 + *X*, +*Y*, +*Z*
N1_2–H1A_2···O2_3	0.91	1.79	2.702(4)	177.6	–1 + *X*, +*Y*, +*Z*
N1_3–H1A_3···O2_1	0.91	1.85	2.755(4)	171.4	–1 + *X*, –1 + *Y*, +*Z*
N2_7–H2_7···O1_9	0.88	2.24	2.973(5)	140.4	1 – *X*, 0.5 + *Y*, −*Z*
C11_7–H11_7···O4_7	0.95	2.31	2.872(5)	117.0	
N2_8–H2_8···O5_9	0.88	2.16	2.964(5)	152.4	1 – *X*, 0.5 + *Y*, 1 – *Z*
C16_8–H16A_8···O5_9	0.98	2.42	3.299(6)	148.5	1 – *X*, 0.5 + *Y*, 1 – *Z*
O2_9–H2B_9···O1_9	0.86(3)	2.03(3)	2.835(4)	154(5)	
O2_9–H2A_9···O3_7	0.88(3)	1.94(3)	2.810(4)	170(6)	
O3_8–H3A_8···O4_9	0.84	1.76	2.565(4)	158.6	
O1_9–H1A_9···O4_7	0.85(3)	1.87(3)	2.685(4)	161(5)	–*X*, –0.5 + *Y*, −*Z*
O3_7–H3A_7···O3_9	0.84	1.76	2.596(4)	172.0	
O3_9–H3A_9···O2_9	0.85(3)	1.86(3)	2.699(5)	168(6)	1 + *X*, +*Y*, +*Z*
O4_9–H4B_9···O1_4	0.84(3)	1.94(3)	2.762(4)	166(6)	2 – *X*, 0.5 + Y, 1 – *Z*
O5_9–H5A_9···O4_8	0.84(3)	1.84(3)	2.678(4)	176(5)	2 – *X*, –0.5 + *Y*, 1 – *Z*

#### 3.2.7 TXA-MAL (1:1) Salt

The crystal structure was solved in the orthorhombic space group *Pbca*. The asymmetric unit contains one molecule each of TXA and MAL (Figure 2a), and the unit cell packing includes eight formula units (Figure 11a). The TXA-MAL salt is formed via proton transfer from one carboxylic acid group of MAL to the carboxylate group of TXA. The components exist as a TXA ammonium cation (^+^H_3_N–C_6_H_10_–COOH) and a malonate monoanion (^−^OOC–CH_2_–COOH) and interact via NH_3_^+^···^–^OOC to confirm salt formation. The structure is stabilized by a network of strong, charge-assisted hydrogen bonds: NH_3_^+^···^–^OOC, between TXA ammonium and malonate carboxylate to establish a salt framework. As observed for previous TXA-carboxylate acid coformer systems, the carboxylate group of TXA is protonated by the carboxylic acid groups of MAL. The −NH_3_^+^ group of TXA acts as a triple donor, trifurcated system, forming ionic N^+^–H···^–^O bonds with the carboxylate oxygen atoms of the malonate monoanion: N1–H1A···O3 (2.8319(11) Å, 127.2°), N1–H1C···O1, (2.8633(11) Å, 155.4°), and N1–H1B···O6 (2.8035(11) Å, 165.7°). The MAL monoanion forms a strong, charge-assisted intramolecular O–H···^–^O hydrogen bond (O5–H5A···O3, 2.5249(10) Å), which is a common feature that stabilizes the anion, and self-assembles into extended chains that increase conformational rigidity (Figure 11a–c). The hierarchical network of these ionic N^+^–H···^–^O and strong O–H···^–^O hydrogen bonds forms undulating two-dimensional layers. These layers stack into a three-dimensional framework through additional weak C–H···O contacts (C10–H10A···O3, 2.80 Å; C5–H5···O1, 2.85 Å; C8–H8A···O5, 3.02 Å; C6–H6A···O1, 2.89 Å) and van der Waals interactions of the cyclohexane rings (Figure 11c). The extensive network of N^+^–H···^–^O and O–H···^–^O H-bonds stabilizes the crystal lattice. The crystalline packing and 3D network of interactions are presented in Figure 11e, with hydrogen-bond geometry detailed in Table 9.

**Figure 11:**
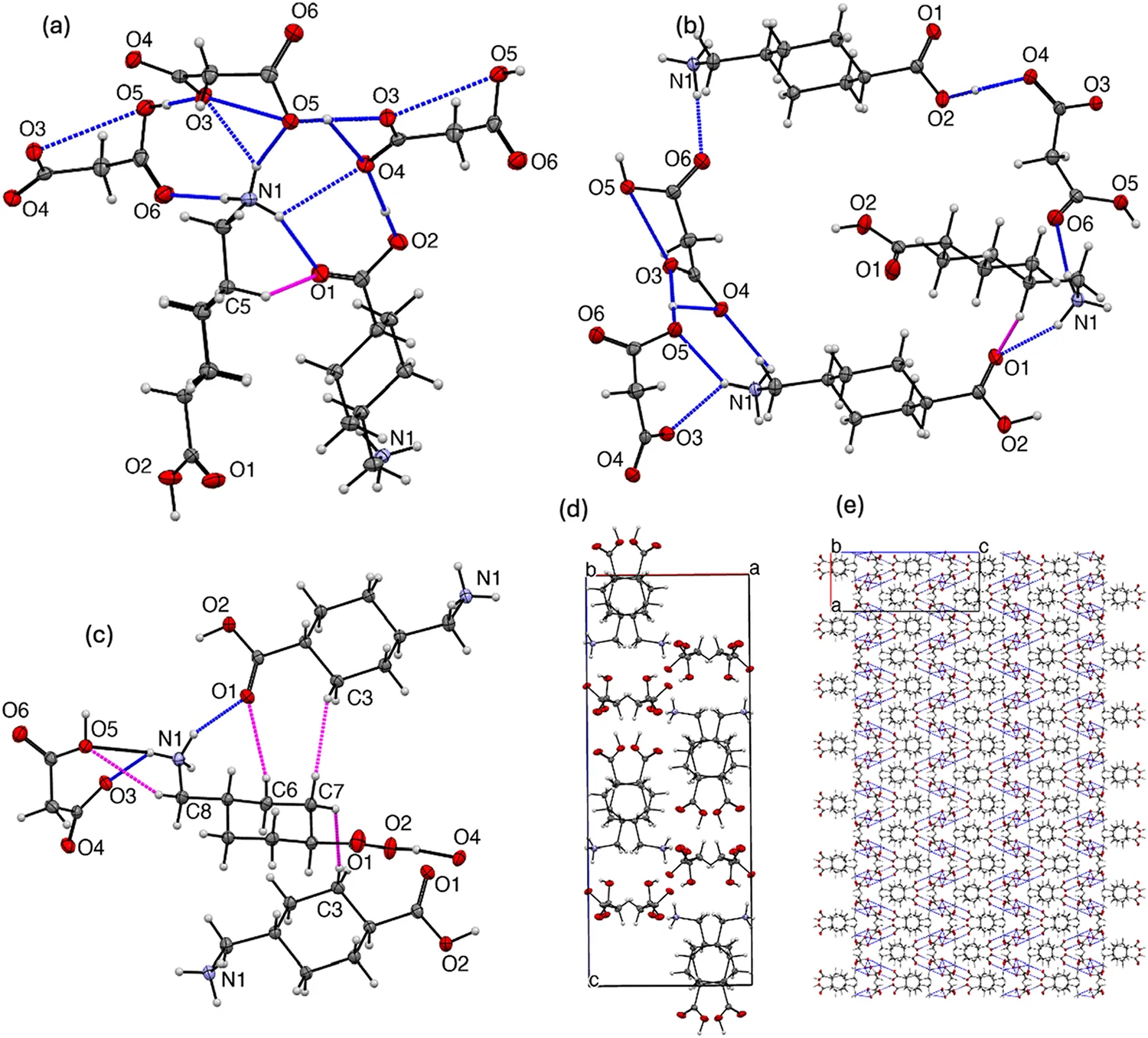
TXA–MAL hydrogen bonding and molecular packings: (a) N–H···O and O–H···O interactions (blue); (b) weak C–H···O contacts (magenta); (c–e) packing and infinite chain. Ellipsoids at 50% probability; labels on C and H atoms in the structures are omitted for clarity.

**Table 9:** Hydrogen Bond Geometry of TXA-MAL

D–H···A [Å]	d(D–H) [Å]	d(H···A) [Å]	d(D···A) [Å]	<(DHA) [°]	Symmetry transformations
O2–H2A···O4	0.968(19)	1.642(19)	2.6063(10)	173.3(18)	
O5–H5A···O3	0.97(2)	1.56(2)	2.5249(10)	176.4(19)	1 – *X*, –0.5 + *Y*, 1.5 – *Z*
N1–H1A···O3	0.897(16)	2.198(15)	2.8319(11)	127.2(12)	1.5 – *X*, 1 – *Y*, –0.5 + *Z*
N1–H1A···O5	0.897(16)	2.316(16)	3.0314(11)	136.6(13)	1.5 – *X*, 1 – *Y*, –0.5 + *Z*
N1–H1B···O6	0.935(16)	1.888(17)	2.8035(11)	165.7(14)	0.5 + *X*, 0.5 – *Y*, 1 – *Z*
N1–H1C···O1	0.919(16)	2.002(16)	2.8633(11)	155.4(13)	0.5 + *X*, 1.5 – *Y*, 1 – *Z*
N1–H1C···O4	0.919(16)	2.551(15)	3.0946(11)	118.3(11)	0.5 + *X*, 1.5 – *Y*, 1 – *Z*

#### 3.2.8 TXA-PIC (1:1) Salt

The crystal structure was solved in the monoclinic crystal system with the space group *P2*_*1*_/*c*, containing one TXA molecule and one PIC molecule in its asymmetric unit (Figure 2c). The unit cell packing contains four molecules each of TXA and PIC (Figure 12d). TXA’s ammonium remains protonated (NH_3_^+^), and its carboxylate (COO^–^) was protonated by H ^+^ transfer of the picolinic acid coformer. *TXA-PIC salt* is confirmed by the proton transfer from PIC to the carboxylate group of TXA, generating a TXA^+^ cation (ammonium) and a picolinate^–^ anion that shows delocalized C–O distances of 1.245(3) and 1.254(3) Å. As illustrated in Figure 12a–c, the ammonium (−NH_3_^+^) of TXA acts as a H-bond donor to both the carboxylate (−COO^–^) and pyridyl nitrogen (N) of PIC via two distinct interactions: first, ionic N^+^–H···O^–^ bonds (N1–H1A···O3, 2.859(3) Å; N1–H1C···O4, 2.794(3) Å), and second, charge-assisted N^+^–H···N interaction (N1–H1B···N2, 2.982(3) Å). The bifurcated coordination of −NH_3_^+^ spanning both carboxylate O atoms (O3, O4) and the pyridine N (N2) creates a triple-acceptor motif that stabilizes the salt framework (Figure 12b). The picolinate anion is present as the counterion; hence, formation of an O–H···O^–^ interaction between TXA (−COOH) and PIC (−COO^–^): O2–H2···O1 (2.743 Å) is observed with additional stability to the crystal packing. Notably, the N^+^–H···O^–^ bonds (<2.9 Å), a characteristic of TXA-multicomponent salts, dominate the structure with ionic character, while the bent N^+^–H···N interaction observed only in TXA-PIC introduces supplementary stability to the structure. The multifaceted H-bond networks (N^+^–H···O^–^ and N^+^–H···N), shown in Figure 12a,b, drive the formation of 2D sheets via the strong ionic/charge-assisted synthons, which further stack into 3D through weak C–H···O contacts; C11–H11···O1 (3.475(3) Å) and C2B–H2B···O2B (3.15(4) Å). However, the O–H···O^–^/C=O···H–C pair forms a *R*_4_^4^(32) heterosynthon (8-membered ring motif) observed in the structure. The crystalline packing and connected chains in the TXA-PIC structure (Figure 12e–g) are stabilized by a hierarchy of interactions and contacts that reinforce the 3D framework, while the anisotropic displacement of the −NH_3_^+^ group further stabilizes the structure. The H-bond geometry of TXA-PIC is summarized in Table 10, with full crystallographic details in the SI.

**Figure 12:**
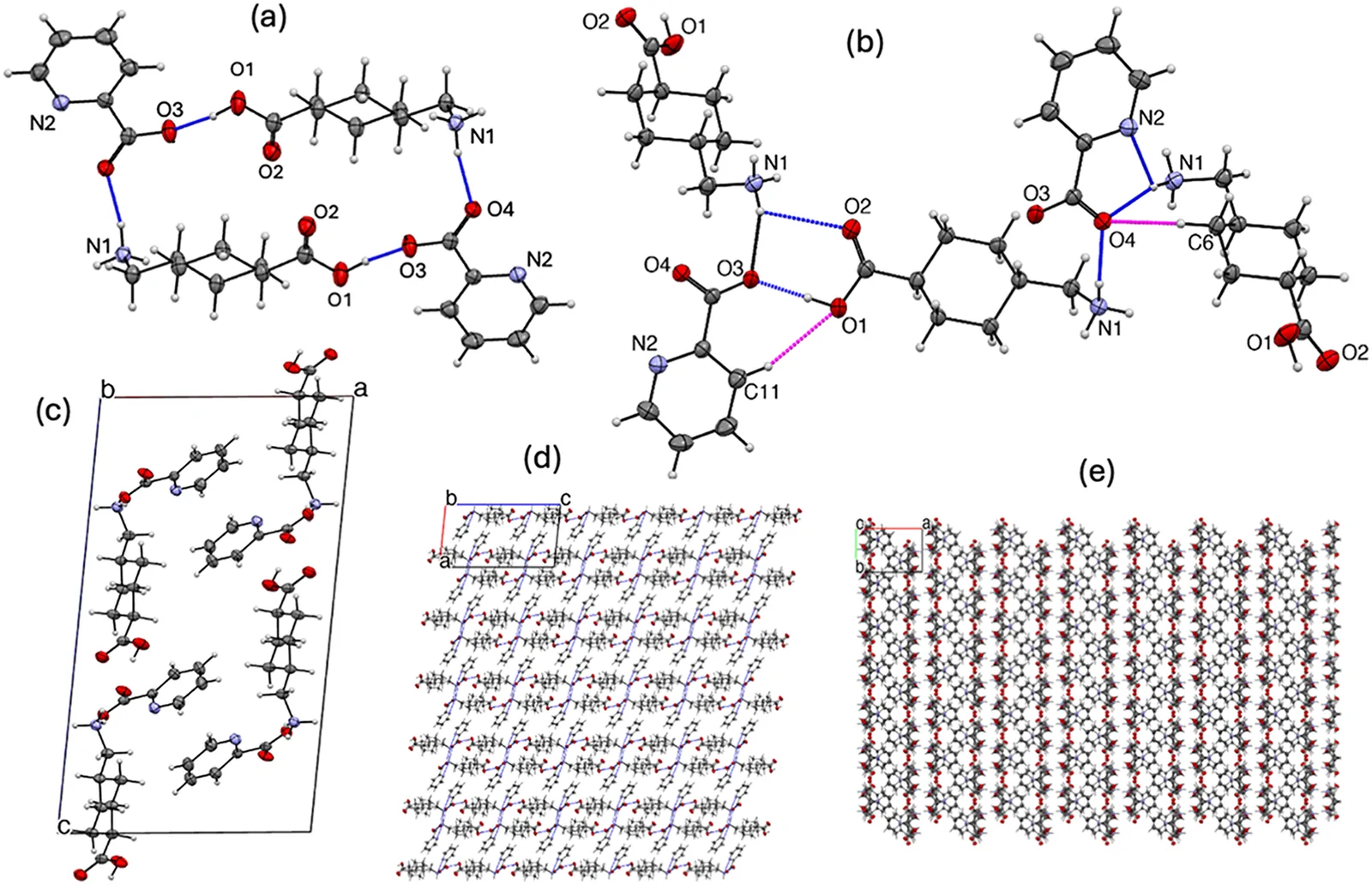
TXA–PIC hydrogen bonding and molecular packings: (a–c) bifurcated coordination via N–H···O and O–H···O interactions (blue) and weak C–H···O contacts (magenta); (c–e) packing and infinite chain. Ellipsoids at 50% probability; labels on C and H atoms in the structures are omitted for clarity.

**Table 10:** Hydrogen Bond Geometry for TXA-PIC

D–H···A	d(D–H) [Å]	d(H···A) [Å]	d(D···A) [Å]	∠(DHA) [°]	Symmetry transformations
N1–H1A···O3	0.91	1.99	2.859(3)	158.2	+*X*, –1 + *Y*, +*Z*
N1–H1B···O4	0.91	2.00	2.870(3)	158.7	
N1–H1B···N2	0.91	2.37	2.982(3)	124.9	
N1–H1C···O4	0.91	1.92	2.794(3)	160.2	–*X*, –0.5 + *Y*, 0.5 – *Z*
O1–H1···O3	0.98	1.76	2.743	I76	
C2B–H2B···O2B	1.00	2.39	3.15(4)	132.2	–*X*, 1 – *Y*, 1 – *Z*
C11–H11···O1	0.95	2.60	3.475(3)	152.9	+*X*, 1.5 – *Y*, –0.5 + *Z*

### 3.3 Powder XRD Analysis

Powder X-ray diffraction (PXRD) was used to confirm the phase purity and crystallinity of all new TXA multicomponent forms and to distinguish them from the starting materials (TXA and the respective coformers). Diffraction patterns were collected over the range 5–40° 2θ (Cu Kα radiation). Pure TXA is identified as an orthorhombic form with space group (*P2*_*1*_ *2*_*1*_ *2*_*1*_) crystal,^21^ showing intense diffraction peaks at 10.50, a doublet (at 17.48–17.86), 19.38, and its characteristic peak at 21.24 (2θ, °) when using Cu Kα radiation. The diffraction peaks of the multicomponent forms are neither a simple superposition nor a trivial shift of the peaks of pure TXA or the coformers, which demonstrates the formation of new crystalline phases. Figure 13a–d compares the plotted experimental and calculated diffraction patterns (single-crystal data) for TXA multicomponent forms and the starting materials (TXA/coformers), covering a 5–40° 2θ range. The plots include diffraction patterns obtained from multicomponent cocrystal/salt samples after 6 months of stability study, determined under accelerated conditions at 75% ± 5% relative humidity/40 °C ± 2 °C. In Figures 13a–d and 14a–d, the TXA peaks at 14.38, 14.94, 18.08, 18.46, and 25.52 (2θ, °), distinctive from the peaks of TXA multicomponent forms. The diffraction pattern of TXA–ADP (2:1 salt) in Figure 13a, show peaks at 8.52, 9.46, 13.06, 14.94, a doublet at 15.98–16.36, another at 17.86–18.3, along with peaks at 19.98, 22.42, 23.56, and 29.98 (2θ, °), which are distinctive peaks and differs from the characteristic intense peaks of TXA and ADP (at 12.78, 21.4, 31.12, 25.72, and 35.68° (2θ, °). The experimental diffraction peak for TXA–ADP (2:1 salt) aligns well with the calculated diffraction pattern obtained for its single-crystal analysis (Figure 13a). TXA–SAL (1:1 salt) in Figure 13b presents distinctive peaks at 9.90, 10.86, 12.60, 16.50, 23.28 (2θ, °), which differ from the experimental diffraction pattern of starting materials TXA and ASA at 7.72 and 15.54 (2θ, °). The experimental patterns of TXA–CTR (1:1 salt) present major differences at 7.62, 8.58, 13.16, 14.74, 17.14 (2θ, °) compared to those for TXA and CTR (peaks at 14.24, 16.62, a doublet at 17.92–18.22, along with other peaks at 19.72, 23.98, 25.04, 26.34, 28.92, 31.42, and 36.22 (2θ, °) as shown in Figure 13c. The experimental and calculated patterns for TXA–CTR (1:1 salt) align well and differ from those for pure TXA and CTR. Figure 13d shows the diffraction patterns of TXA–APAP·3H_2_O, which presents a unique peak at 7.48, 8.36, 10.60, 13.54, 15.88 (2θ, °) that is not present in either parent compound TXA and APAP (12.26, 13.82, 15.62, 16.78, 18.28, 19.01, 20.52, 23.66, 24.56, 26.72, 32.62, and 37.08 2θ, °). The comparison of the experimental and calculated patterns for TXA-APAP shows good agreement of peaks.

**Figure 13:**
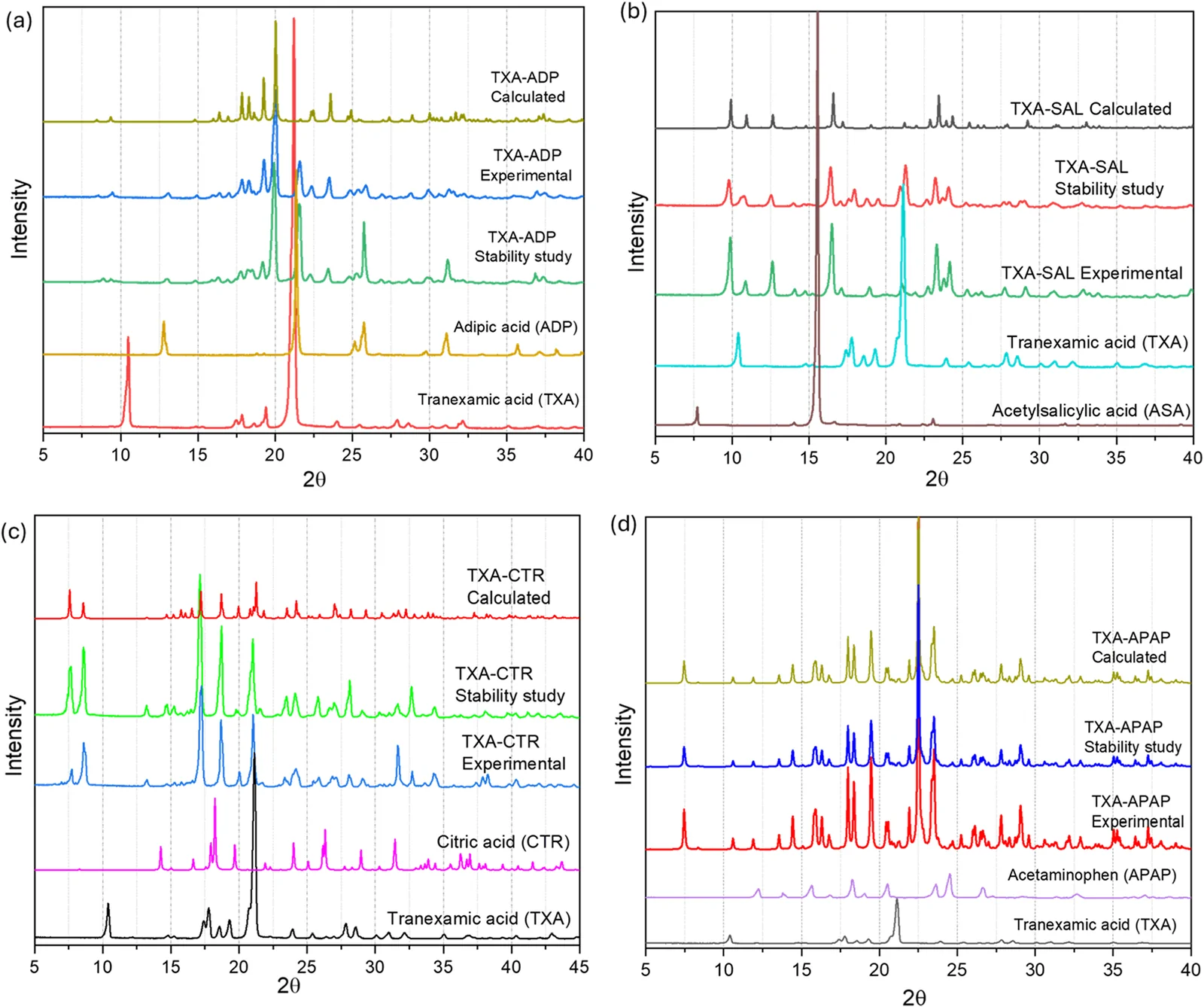
Plots of diffraction patterns for (a) TXA–ADP, (b) TXA–SAL, (c) TXA–CTR, and (d) TXA–APAP samples. Each plot compares the diffractograms of the experimental, calculated, and starting materials, and a sample from the stability study (at 75% RH/40 °C).

**Figure 14:**
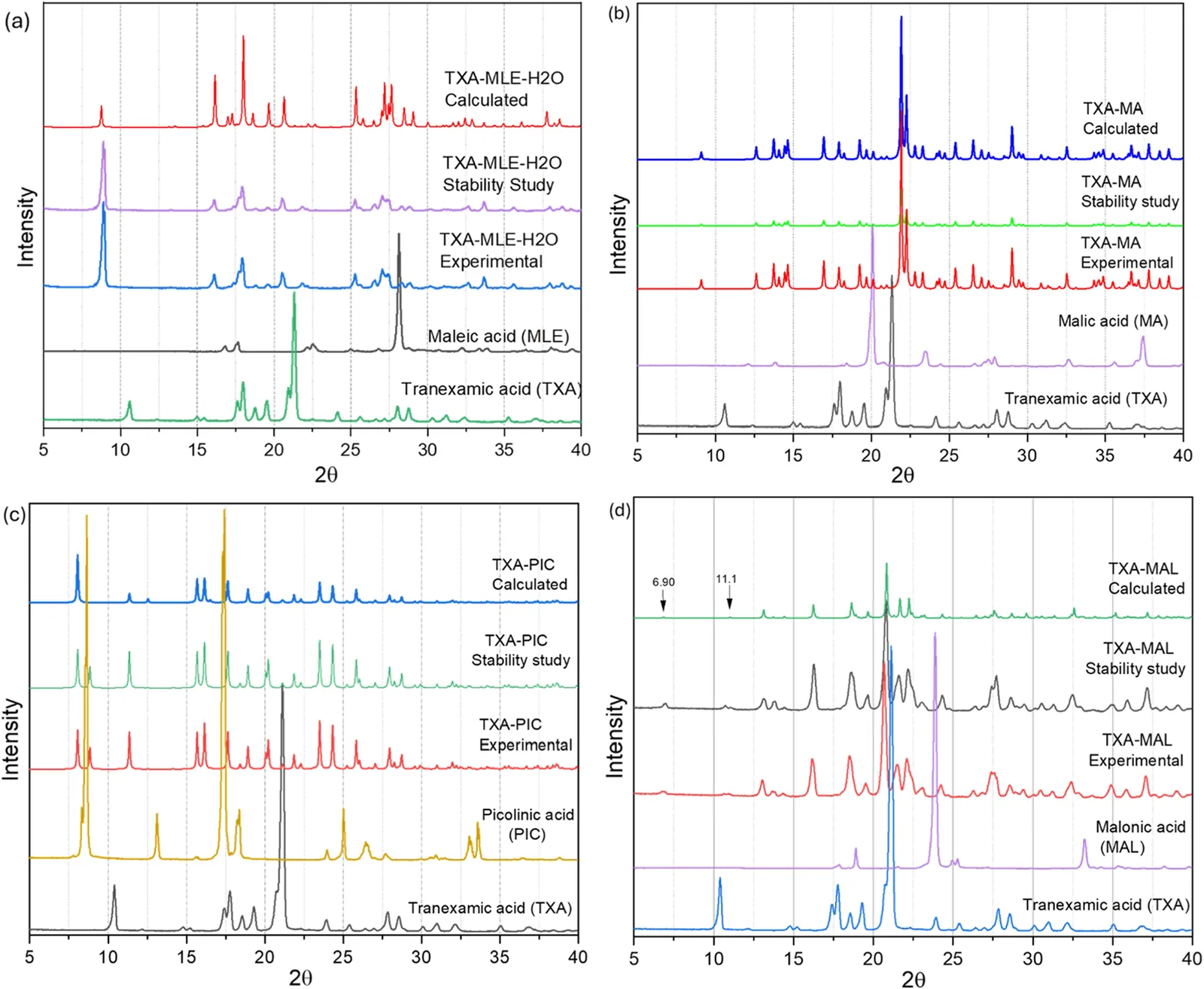
Plots of diffraction patterns for (a) TXA–MLE monohydrate, (b) TXA–MA, (c) TXA–PIC, and (d) TXA–MAL samples. Each plot compares the diffractograms of the experimental, calculated, and starting materials, and a sample from the stability study (at 75% RH/40 °C).

As shown in Figure 14a, the experimental pattern of TXA-MLE monohydrate (peaks at 8.94, 16.06, 17.88, 20.54, 21.80° 2θ) differs completely from pure TXA and MLE (at 16.7, 17.66, 22.5, 26.84, 28.1, 32.24, 33.34, 33.86, 38.02, 39.42, and 40.54 2θ, °), and matches the single-crystal calculated pattern. This TXA-MLE monohydrate pattern is consistent with the previously reported monohydrate,^22^ and the inclusion of this hydrate enhances the understanding and reference of TXA’s structural diversity in the solid state and justifies its inclusion in the subsequent report on in vitro property investigations. In Figure 14b, the experimental pattern of TXA-MA exhibits distinctive peaks at 9.08, 12.62, 14.06, 16.96, and a series of unique doublet peaks at 14.44–14.62, 21.92–22.24, 17.9–18.26 (2θ, °), which differ from TXA and MA (at 20.01, 23.44, 26.6, 35.54, 36.68, and 37.42 2θ, °). The experimental pattern of TXA-MA matches the calculated pattern, confirming the phase purity of the salt. Figure 14c **presents** the experimental pattern of TXA–PIC showing distinctive peaks at 6.04, 8.82, 11.36, doublet (at 15.7–16.18), 17.62, 20.24, 24.3, 24.18, 25.8, 28.01, and 28.76 (2θ, °), with all different from pure TXA and PIC (8.64, 13.16, 17.44, 18.38, 25.04, and 26.5, 2θ, °). The experimental and calculated pattern for TXA-PIC shows excellent agreement, confirming a distinct crystalline phase. Finally, Figure 14d shows the experimental pattern of TXA-MAL displays a set of well-defined peaks at 6.90, 10.90, 11.12, 13.11, 16.22, 18.62, 19.55, 20.65, 21.64, 22.22, 24.26, and 27.51 (2θ, °) that have shifted or are absent in pure TXA and MAL (at 17.82, 18.90, 23.95, and 33.26, 2θ, °). The excellent match of experimental and calculated patterns confirms a highly crystalline phase.

### 3.4 Stability Study

Pure TXA is reported to remain stable under short-term storage but absorbs moisture over time at high humidity levels (>75% RH), potentially leading to degradation. To assess the physical stability of the TXA multicomponent forms under accelerated conditions, each form was stored at 40 °C ±  2 °C and 75 %  ±  5 % RH for six months. Comparison of the initial (*t* = 0) and final (month 6) patterns (Figures 13a–d and 14a–d) reveals that all forms retain their distinctive diffraction peaks, confirming that no bulk phase transformation or degradation occurred. PXRD patterns after six months show that TXA-APAP, TXA-MLE·H_2_O, and TXA-PIC present negligible differences in their PXRD patterns. TXA-MAL exhibited slight peak shifts at 13.72, 20.80, and 27.48 (2θ, °), and TXA-ADP showed altered peaks at 9.44, 18.34, 25.74, and 31.22 (2θ, °) alongside peak broadening and reduced crystallinity. Also, a few changes were observed for TXA-ASA at 17.94, 19.52, and 20.91 (2θ, °). TXA-CTR showed peak shifts at 7.64, 23.48, 25.82, and 38.02 (2θ, °), and TXA-MA presented low-intensity peaks with diminished crystallinity. To provide a quantitative assessment, three metrics was calculated for each form using (i) Pearson correlation coefficient (*r*) between the full pattern at *t* = 0 and month 6 (range 5–40° 2θ), (ii) average absolute peak shift (Δ2θ) for the three most intense nonoverlapping peaks, and (iii) a crystallinity index (ratio of integrated peak area to total scattered intensity, normalized to *t* = 0). The results show that TXA–APAP, TXA–MLE·H_2_O, and TXA–PIC exhibit the highest correlations (*r* > 0.96) with negligible peak shifts (Δ2θ < 0.06°), indicating excellent stability. TXA–CTR, TXA–MAL, and TXA–ADP show moderate drift (*r* = 0.89–0.94, Δ2θ = 0.09–0.15°), while TXA–MA and TXA–SAL exhibit slightly lower correlation (*r* = 0.87–0.88, Δ2θ = 0.16–0.18°). The crystallinity indices remain above 0.83 for all TXA forms, confirming that the observed changes are primarily due to preferred orientation, particle size variation, or minor lattice strain from moisture cycling, rather than chemical instability or phase transformation. For comparison, pure TXA stored under the same conditions showed few changes in its PXRD pattern over six months, confirming its hygroscopic tendency. The minor variations observed for the TXA cocrystal/salts are within acceptable limits for pharmaceutical materials and are consistent with reports on other pharmaceutical cocrystals (e.g., Nangia; Bhatia & Devi; Samie & Alavian).^50–52^ Thus, all engineered TXA forms demonstrate satisfactory physical stability under ICH accelerated storage conditions.

### 3.5 Thermal Analysis

DSC analysis (Figure 15a–i) was performed to determine the melting temperatures and thermal stabilities of the new TXA salts and TXA-APAP cocrystal. As shown in Figure 15a, the DSC curve of pure TXA exhibits a single endothermic peak at 304.7 °C, corresponding to its melting point (MP). The melting points of the pure coformers are as follows: ADP (152.20 °C), APAP (171.17 °C), MA (130.0 °C), PIC (136–139 °C), ASA (135–137 °C), MLE (130–135 °C), CTR (153 °C), and MAL (92.35 °C). The DSC thermogram of TXA-ADP (Figure 15b) displays two endothermic events: a low-temperature **peak at 117 °C** attributed to surface solvent loss and a melting endotherm at 199.2 °C, which lies between the melting points of pure TXA and ADP. The small endotherm at 117 °C in TXA-ADP corresponds to desolvation (loss of trace surface solvent or loosely bound solvent molecules), not melting or degradation, as supported by SCXRD. The DSC thermogram of TXA-MA (Figure 15c) shows a broad melting endotherm with a peak at 208.3 °C (Tonset: 164.20 °C and Toffset: 256.50 °C), intermediate to the MPs of TXA and MA. The broad melting endotherm at 208.3 °C is accompanied by a continuous baseline shift below 50 °C, indicating simultaneous melting and decomposition; SCXRD confirms no solvent inclusion. The DSC thermograms of TXA-MAL (Figure 15d) and TXA-PIC (Figure 15e) exhibit melting endotherms at 190.95 and 191.03 °C, respectively, both intermediate between the melting points of their pure components. The baseline endothermic events observed in the low-temperature region (50–120 °C) in both salts are attributed to loss of trace surface solvent or loosely bound solvent molecules, consistent with the hygroscopic nature of TXA. SCXRD confirmed the absence of solvent inclusion in TXA-MAL and TXA-PIC structures. Similarly, the thermogram for TXA-APAP cocrystal hydrate (Figure 15f) exhibits two endothermic events: a broad peak at 82.62 °C (loss of 3 water molecules; SCXRD confirmed hydrate) and a doublet melting endotherm with peaks at 157.81 °C and 167 °C (corresponding to dissociated polymorphic form II and I of APAP),^53^ followed by its melting endotherm with a peak at 257.22 °C. This suggests the salt dissociates upon heating to liberate water and APAP before melting. This melting endotherm of TXA-APAP is observed as intermediate between the MPs of pure TXA (304.7 °C) and APAP (171.17 °C). The DSC thermogram of TXA-MLE (Figure 15g) shows two endothermic events: a peak at 64 °C attributed to water loss, and a broad melting transition appearing as a doublet with peaks at 130.22 °C (Tonset: 88 °C) and 146 °C (Tonset: 135.4 °C). This result is consistent with the SCXRD analysis, which confirmed the TXA-MLE monohydrate salt. The melting endotherm of this monohydrate salt appears at an intermediate temperature between those of pure TXA and MLE. The DSC thermogram of TXA-SAL (Figure 15h) exhibits a melting endotherm at 201 °C, which is intermediate to the melting points of pure TXA and ASA. Similarly, the thermogram of TXA-CTR (Figure 15i) shows a sharp melting endotherm at 170.3 °C, which, like TXA-SAL, is intermediate to its pure components (TXA and CTR). The distinct melting endotherms observed in the DSC thermograms of all TXA multicomponent crystals confirm their identity as novel chemical entities.

**Figure 15:**
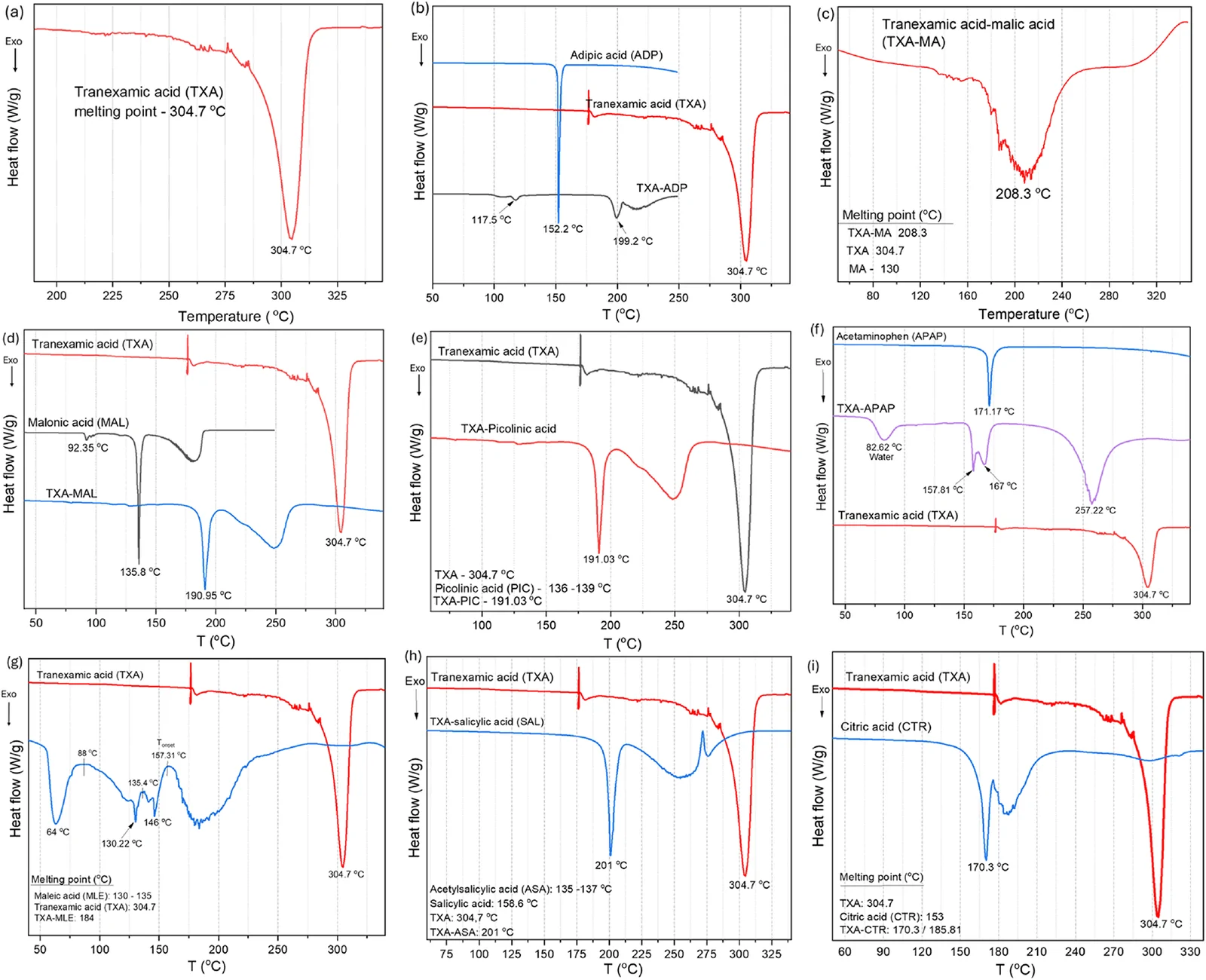
DSC curve comparison of TXA/coformers and multicomponent forms: (a) TXA; (b) TXA-ADP; (c) TXA-MA; (d) TXA-MAL; (e) TXA-PIC; (f) TXA-APAP; (g) TXA-MLE; (h) TXA-SAL; and (i) TXA-CTR.

## 4 Conclusions

The low membrane permeability and variable oral bioavailability (30–40%) of TXA hinder its rapid emergency use due to its poor transcellular absorption. Its zwitterionic nature at physiological pH limits passive diffusion across lipid membranes, including the gastrointestinal (GI) tract and blood-brain barrier. Structural modification via inclusion of a stable, favorably interacting coformer in the crystal lattice of TXA, achieved by cocrystallization or salt formation, could optimize its membrane permeability and oral bioavailability. Herein, we report the synthesis and characterization of a TXA cocrystal and salts, engineered through strategic hydrogen and ionic bonding interactions. Salt formation occurs with stronger carboxylic acids, where the energetic gain from creating an extensive network of strong, charge-assisted N^+^–H···^–^O hydrogen bonds in the solid state stabilizes the ionic pair, even against an ostensibly unfavorable Δp*K*_a_. In contrast, TXA-APAP cocrystal hydrate emerges in the absence of proton transfer; here, stability is achieved through optimal supramolecular assembly between the TXA zwitterion and neutral coformers via neutral hydrogen bonds, without new ion formation. Characterization by single-crystal and powder X-ray diffraction confirmed unique diffraction patterns, structural novelty, and key intermolecular interactions. Powder XRD analysis indicated that the TXA multicomponent forms exhibited satisfactory physical and kinetic stability over six months under accelerated conditions (40 °C ± 2 °C/75% ± 5% RH). Although minor changes were observed in some diffraction patterns, structural integrity was maintained. The TXA cocrystal/salts produced exhibit characteristics of new chemical entities, as evidenced by distinct melting temperatures and endotherms intermediate to those of pure TXA and the coformers. In perspective, these findings justify further in vitro investigation and stability of the new TXA salts and cocrystal, especially in combination with oxytocin

## Supplementary Material







## Notes

CIF File TXA Multicomponents (ZIP)
Report TXA CIF Files (ZIP)
Spectroscopic techniques (Figures S1–S6; Tables S1–S2); crystallography method (Figures S7–S10); PXRD patterns (Tables S4–S8; Figure 11); and thermal method (Table 12) (PDF)
