# Supplementary material for: Antifibrinolytic Agent: Synthesis and Characterization of Cocrystal and Salts of Tranexamic Acid
Source: ACS Omega. 2026 Oct 22;11(43):66176–200. doi: 10.1021/acsomega.6c03598 (PMC13737987; doi:10.1021/acsomega.6c03598)
Supplement: Supplementary file 1 [file ao6c03598_si_003.pdf]

Antifibrinolytic Agent: Synthesis and Characterization of Cocrystal and Salts of Tranexamic Acid.

Bolaji C. Dayo Owoyemi,[1,2*] Adedamola E. Adehinmoye,[1] Matthias Zeller,[3] Gabriel L. B. de Araujo.[2] Stephen R. Byrn.[1], Kari Clase,[4*]

[1]Department of Industrial and Molecular Pharmaceutics, College of Pharmacy, 575 Stadium Mall Drive, Purdue University, West Lafayette, IN 47907, USA.
[2]Department of Pharmacy, Faculty of Pharmaceutical Science, University of São Paulo, Av. Professor Lineu Prestes, 580 – Cidade Universitária, São Paulo - CEP 05508-000 – SP, Brazil.
[3]Department of Chemistry, 560 Oval Drive, Purdue University, West Lafayette, IN 47907, USA.
[4]Department of Agriculture and Biological Engineering, 225 S. University Street, Purdue University, West Lafayette, IN, 47907, USA.

*Corresponding authors: kclase@purdue.edu; bdayoowoyemi@usp.br.

ABSTRACT.

Tranexamic acid (TXA) is a potent antifibrinolytic agent with poor oral bioavailability (~35–40%) due to its high polarity and zwitterionic nature, while physicochemical factors, including its structural instability, further hinder its absorption. In this study, the crystal engineering concept is applied to enhance the physicochemical properties of TXA through targeted structural modifications, resulting in the synthesis of pharmaceutically stable cocrystal and salt forms. The application of supramolecular synthon design, pKa rule, and structural geometry (molecular mimicry), resulted in the rational design, synthesis, and structural characterization of eight binary multicomponent forms of TXA: a *TXA-adipate salt*, monoclinic space group $P2_1/n$; a *TXA-salicylate salt*, monoclinic space group $P2_1/n$; a *TXA-picolinate salt*, monoclinic space group $P2_1/c$; a *TXA-acetaminophen cocrystal hydrate*, monoclinic space group $P2_1$; a *TXA-malonate salt*, orthorhombic space group *Pbca*; a TXA-*maleate salt hydrate*, monoclinic space group $P2_1/c$; a *TXA-malate salt*, monoclinic space group $P2_1/c$; and a *TXA-citrate salt*, triclinic space group $P\bar{1}$. These multicomponent forms were obtained via different hydrogen bonding interactions, some of them charge-assisted (N–H···O, N^+–H···O^-, O−H···O, and O−H···O^-) between NH_2 and/or –OH groups of TXA and the carboxyl (–C(O−)=O) functional groups of the coformers. In the cocrystals, TXA exists as zwitterions (zero charge), whereas in the salts, it carries a positive charge due to the protonation of its carboxylic (–COOH) and ammonium (–NH_3^+) groups. The NH_3^+ to COO^- charge-assisted H-bond provides stabilization to both the coformer COO^- as well as the TXA's NH_3^+. Solid-state characterization using Raman and FT-Raman spectroscopy, thermal techniques (DSC), and X-ray diffraction analysis (powder and single-crystal XRD) supports and confirms the structural novelty. The superior stability profiles of the new TXA cocrystal and salts under standard conditions (40 °C ±2 °C, 75% ±5% RH) establish an improved structure-property profile compared to pure TXA.

Keywords: Tranexamic acid; Antifibrinolytic; Noncovalent bonding; Cocrystal; Salt; Hydrogen bonding; Stability.

Supporting Information (S.I.)

1. Spectroscopy techniques.

The vibrational solid-state characterization using Raman and FT Raman spectroscopy aids screening for evidence of intermolecular interactions between TXA and coformers and distinguishes between neutral and ionic interactions. Evidence of intermolecular interactions (noncovalent and ionic interactions) is observed using the changes observed in peak shift, intensity, and the presence of new peaks in the Raman and FT-Raman spectra in **Figures S1** and **S2.**

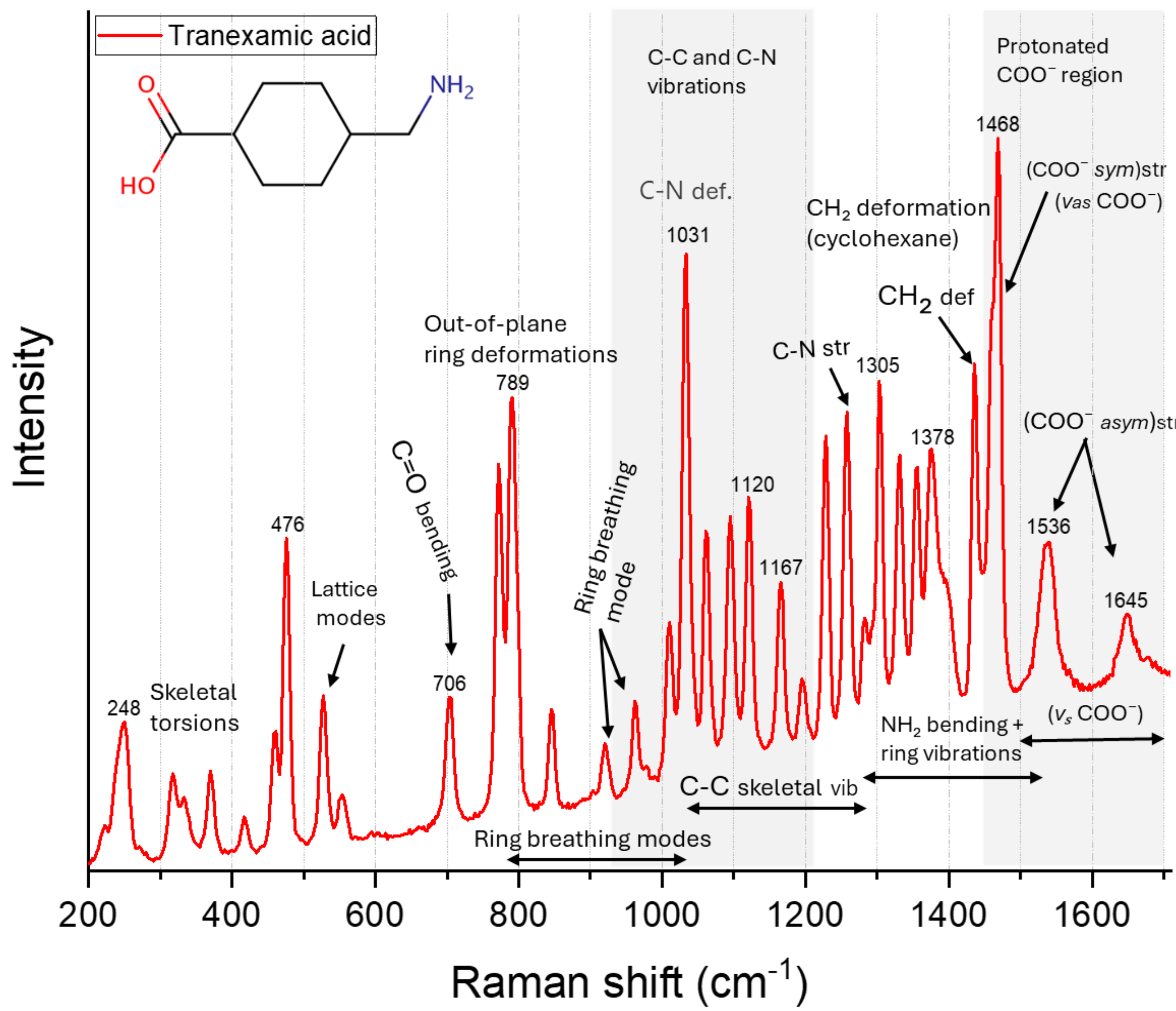

Figure S1. Raman spectra of Tranexamic acid (TXA)

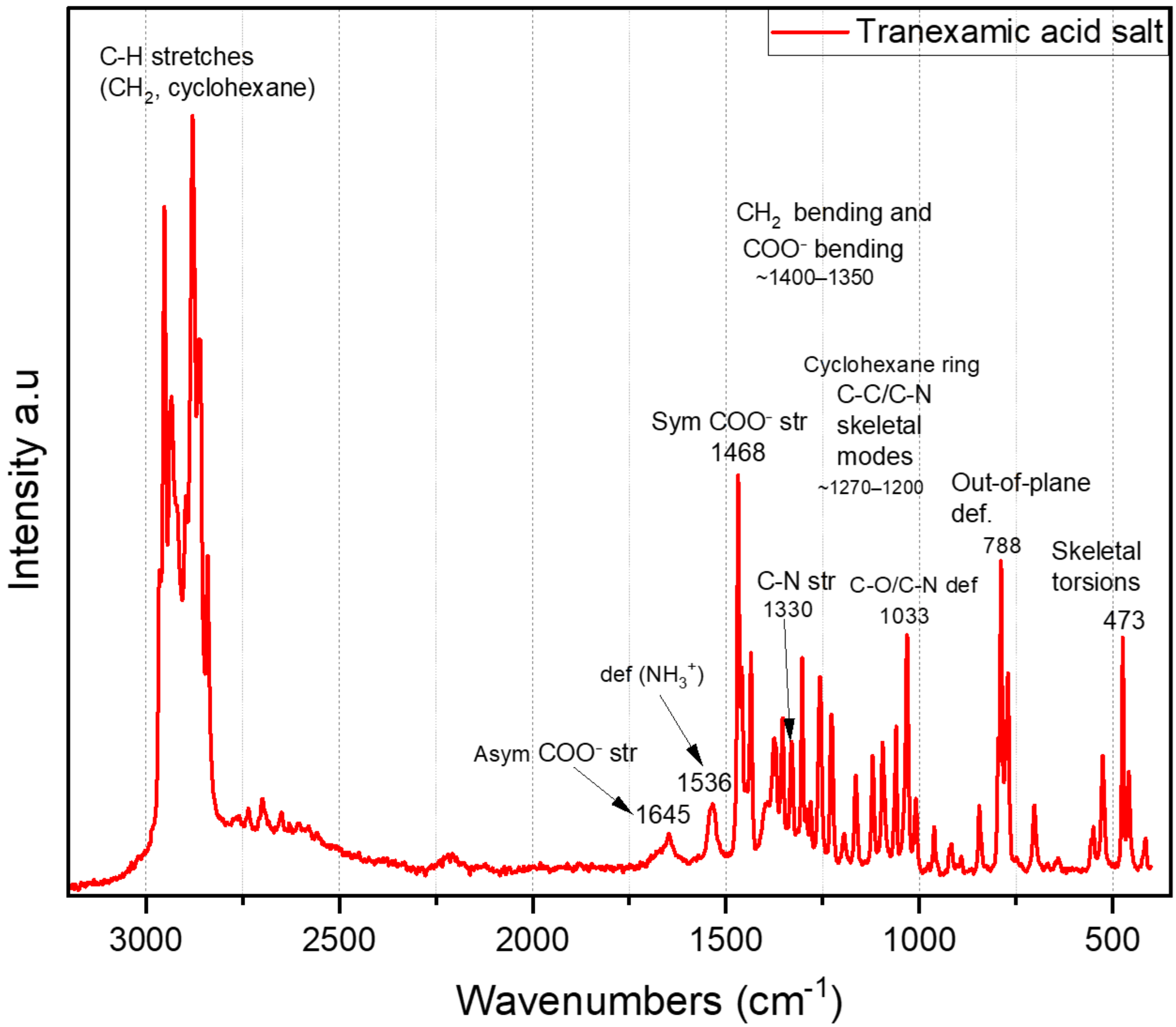

Figure S2. FT-Raman spectra of Tranexamic acid (TXA)

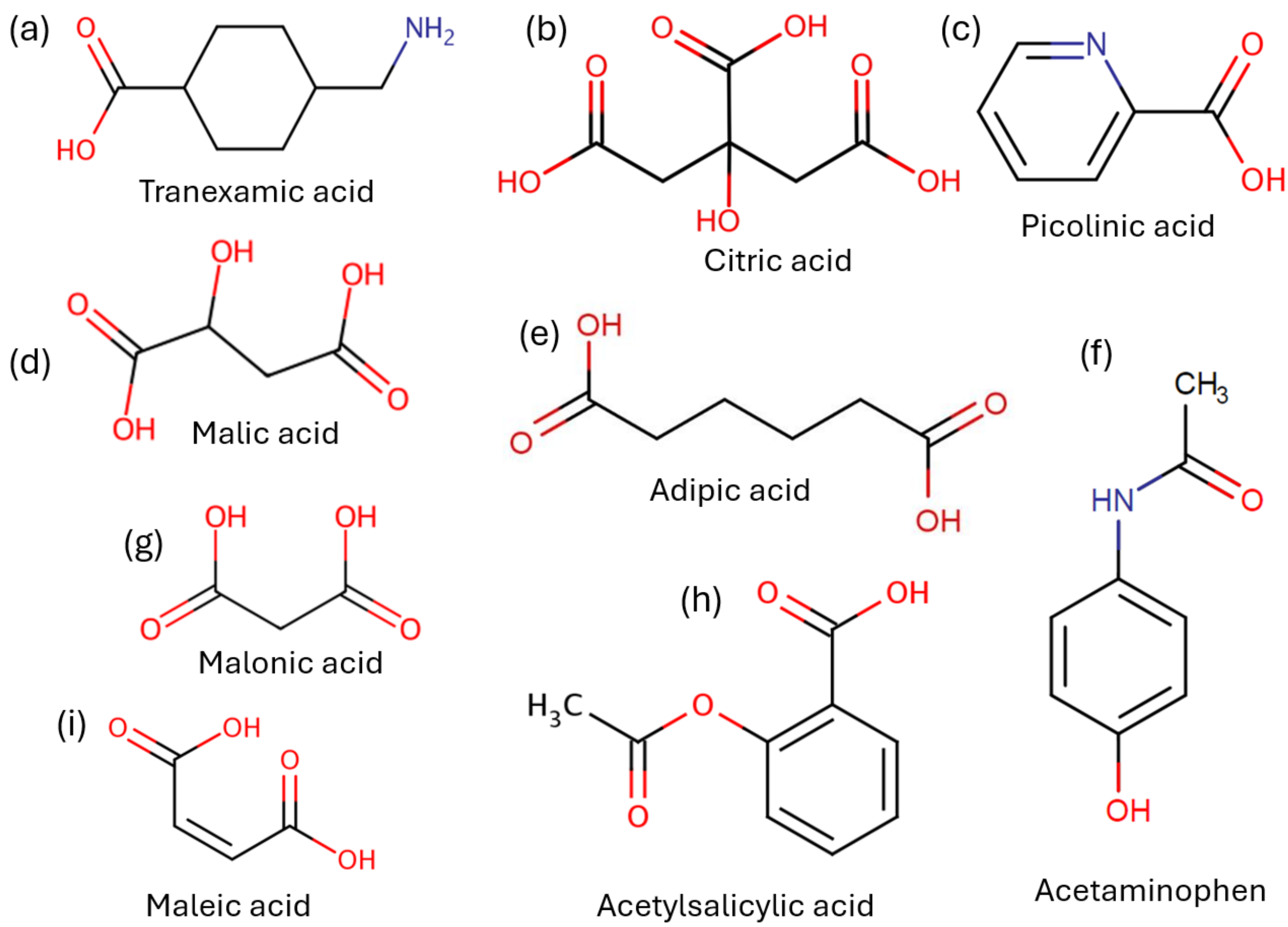

Figure S3. Sketched representation of the chemical structure of tranexamic acid and coformers

Prediction of salt/cocrystal formation between tranexamic acid (TXA) and the coformers based on the given ΔpKa values:

In the solid state, TXA exists as a zwitterion, with a pKa value of 10.4 for the ammonium cation (NH_3^+), and 4.3 for the conjugate acid of the carboxylate anion (COO-). For cocrystal formation, as observed for TXA-APAP, TXA acts as a base, and the ΔpKa calculation requires the pKa of the latter, which is 4.3, and needs to be used. The coformers (mostly carboxylic acids) exhibit pKa1 values between 1.0 and 4.20 and pKa2 values above 4.70. The propensity of salt/cocrystal formation with TXA acting as a base (i.e., the carboxylate anion acting as a proton acceptor, pKa(COOH) = 4.3) was calculated and is summarized in **Table S1.**

Table S1. Salt vs. Cocrystal Prediction Table showing refined predictions of salt/cocrystal formation between tranexamic acid (TXA) and the coformers based on the given ΔpKa values.

**Acid (Coformer) pKa1, (pKa2)**	**ΔpKa1**	**ΔpKa2**	**Predicted**	**Observed**	**Acid (Coformer) pKa1, (pKa2)**
Malic (MA) 3.40, (5.11)	+0.90	–0.81	Cocrystal	2:1 Salt	Malic (MA) 3.40, (5.11)
Malonic (MAL) 2.83, (5.69)	+1.47	–1.39	1:1 Salt or Cocrystal	1:1 Salt	Malonic (MAL) 2.83, (5.69)
Picolinic (PIC) 1.08, (5.20)	+3.22	–0.9	1:1 Salt	1:1 Salt	Picolinic (PIC) 1.08, (5.20)
Maleic (MLE) 1.92, (6.23)	+2.38	–1.93	1:1 Salt or Cocrystal	1:1 Salt hydrate	Maleic (MLE) 1.92, (6.23)
Adipic (ADP) 4.14, (5.41)	–0.11	–1.11	Cocrystal	2:1 Salt	Adipic (ADP) 4.14, (5.41)
Acetylsalicylic (ASA) 3.49	+0.81		Cocrystal	1:1 Salt	Acetylsalicylic (ASA) 3.49
Acetaminophen (APAP) 9.38	–5.08		Cocrystal	3:1 Cocrystal hydrate	Acetaminophen (APAP) 9.38
Citric acid (CTR) 3.13, (4.76)	+1.17	–0.46	1:1 Salt or Cocrystal	1:1 Salt	Citric acid (CTR) 3.13, (4.76)

When TXA acts as a base (amine, pKa = 10.4):

Salt formation is strongly favored with MA, MAL, PIC, MLE, ASA, and CTR (ΔpKa ≥ 3).

Intermediate tendency (1 < ΔpKa < 3) for ADP and APAP (may form salt or cocrystal); however, only APAP forms a cocrystal with TXA.

When TXA acts as an acid (COOH, pKa = 4.3):

Cocrystal formation is favored with MA, MAL, PIC, MLE, ASA, and CTR (ΔpKa ≤ 1).

Salt formation is favored with APAP (ΔpKa = 5.08). Intermediate tendency with ADP (ΔpKa = 0.11).

Raman and FT-Raman spectroscopy Screening.

Using **Figure 4S**, observation of a New/Unique Spectrum "Red Line" is the purported 1:3 TXA-APAP cocrystal, which is distinctly different from a simple superposition of the two pure components. Blue Line (Paracetamol): Shows characteristic sharp peaks (e.g., around 800–860 cm^{-1} and 1230–1300 cm^{-1} typical for amide and aromatic ring breathing). Green Line (Tranexamic Acid): Shows characteristic peaks (e.g., strong carboxylate or carboxylic acid stretches, and distinct skeletal vibrations around 700–900 cm^{-1}). Red Line (Cocrystal/Product) shows peak shifts and changes in relative intensity that are not present in either pure component.

Evidence of Hydrogen Bonding (Peak Shifts). In FT-Raman spectroscopy, cocrystal formation is confirmed by the disruption of the original hydrogen bonding networks (self-association) and the formation of new heteromeric interactions (between the two molecules, TXA and APAP). Carbonyl Region (1600–1700 cm^{-1}) in **Figure S4**: There is a visible shift in the red line compared to the blue line. Paracetamol typically shows a sharp Amide I band (C=O stretch) around 1650–1660 cm^{-1}. In the red spectrum, this band appears to have shifted or broadened significantly, indicating that the amide group of paracetamol is now interacting with the carboxylic acid group of tranexamic acid rather than existing in its pure crystalline form.

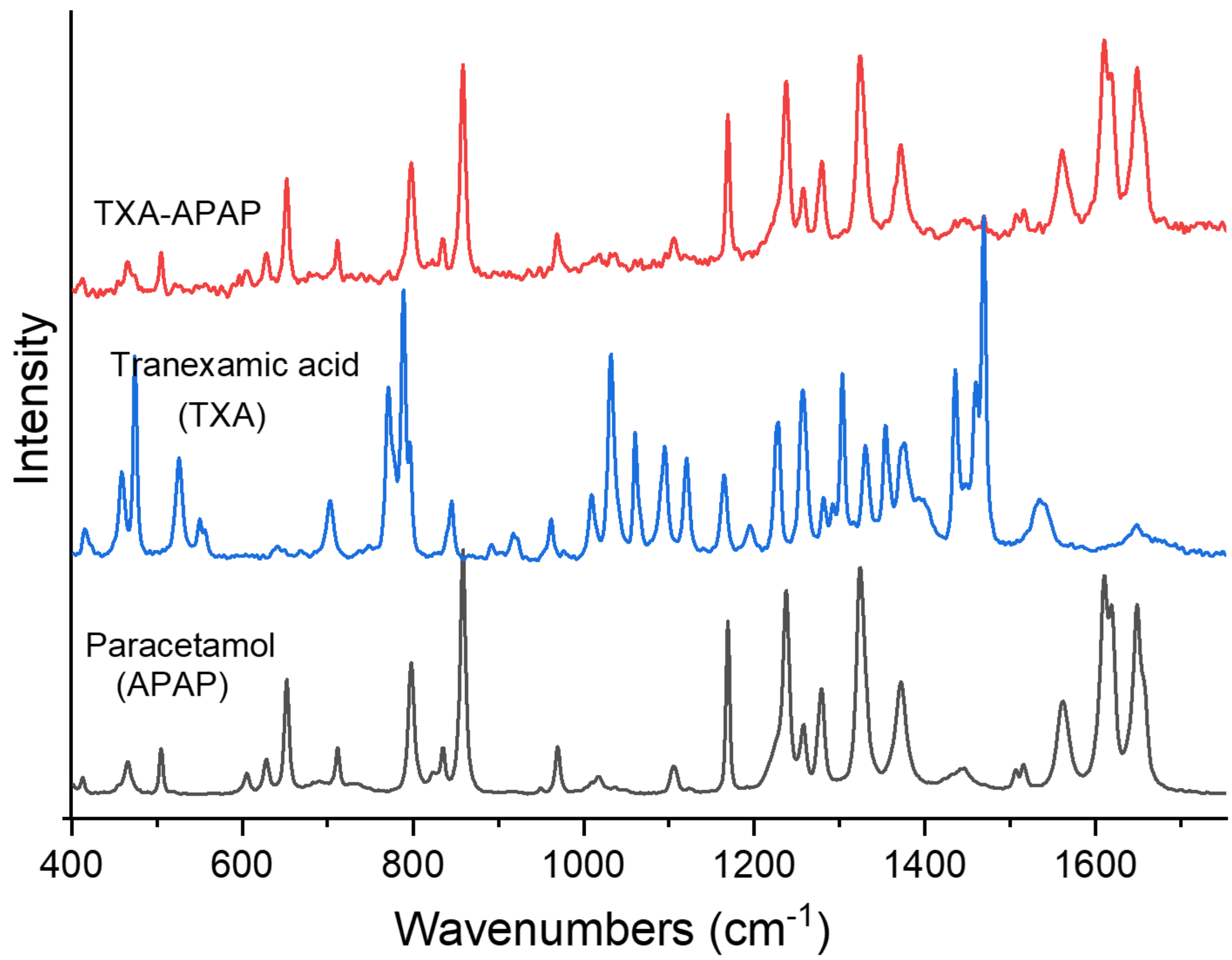

Figure S4. FT-Raman spectra comparison of Tranexamic acid (TXA), Paracetamol (APAP), and TXA-APAP cocrystal.

Carboxylic Acid Region: Tranexamic acid shows specific bands related to its zwitterionic or carboxylic form. The red line does not simply mimic the green line; it shows modifications indicating that the acid group is now participating in a new hydrogen bond with the amide of paracetamol.

Absence of Superimposition (Additivity Rule). A key indicator of a new solid phase (cocrystal) is the failure of the "additivity rule." If the red line were simply a physical mixture, the spectrum would contain all the peaks of the blue line and all the peaks of the green line simultaneously (just at lower intensity).

The spectrum confirms cocrystal formation because the red line represents a new crystalline phase where the molecular vibrations (phonon modes and intramolecular bonds) have been altered due to the formation of specific intermolecular interactions (likely O–H···O and N–H···O hydrogen bonds) between tranexamic acid and paracetamol, rather than a simple mixture of the two individual crystals.

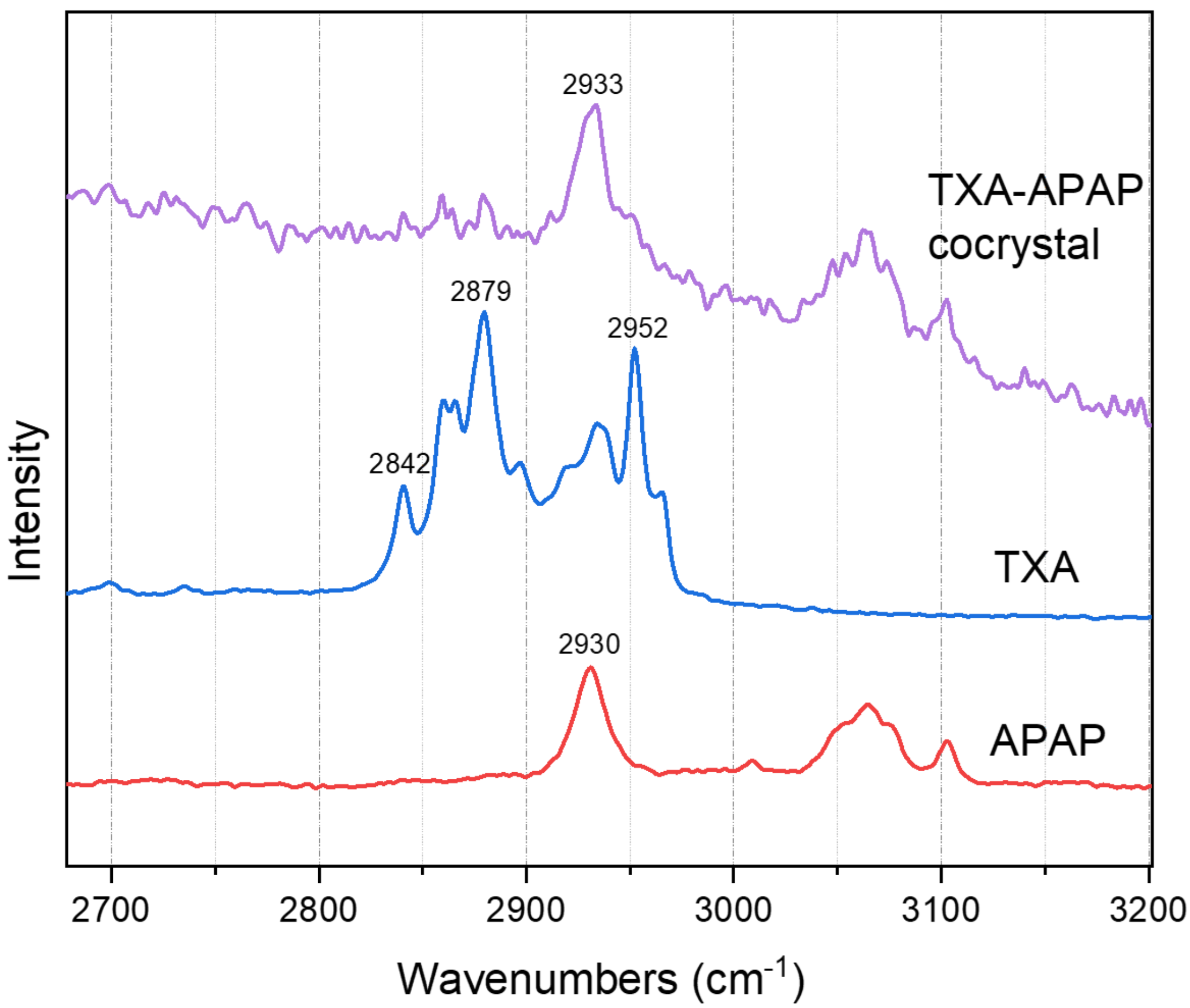

Figure S5. FT-Raman spectra comparison of Tranexamic acid (TXA), Paracetamol (APAP), and TXA-APAP cocrystal.

The high-frequency region (2700–3200 cm^{-1}) for FT-Raman spectra comparison of TXA, APAP, and TXA-APAP cocrystal in **Figure S5** provides critical evidence for hydrogen-bonding interactions involving N–H and O–H donors. In all TXA salt forms (e.g., TXA-CTR, TXA-ADP, TXA-MLE), a broad and intense feature centered near ~3000 cm^{-1} is observed, which we assign to overlapping N–H^{+} stretching vibrations from the protonated ammonium group (NH_3^+) of TXA and O–H stretching contributions from coformer hydroxyl groups (e.g., malic, citric, salicylic acids). This broadening is characteristic of strong, charge-assisted hydrogen bonds (N^{+}–H···$^{-}$O and O–H···$^{-}$O) that lower and widen the stretching frequencies. In contrast, the cocrystal hydrate TXA-APAP displays broader bands and a narrower band near 3040 cm^{-1}, which is assigned to aromatic C–H stretching from the APAP coformer, consistent with the absence of strong ionic interactions. The distinction between these spectral features confirms the different hydrogen-bonding environments in salts versus cocrystals, supporting the solid-state assignments derived from SCXRD.

TXA-Salicylic acid.

Preparation: TXA–SAL (1:1). TXA (157.21 mg, 1.00 mmol) and ASA (180.20 mg, 1.00 mmol) were weighed into a 20 mL beaker and dissolved using methanol (10 mL) and water (5 mL). The solution was slightly heated (50 °C), stirred for 10 minutes, and covered with perforated polythene for slow solvent evaporation at RT. Crystals (~276 mg / > 95%) were obtained after 3 days, filtered, and dried. ASA hydrolyzed in situ during the crystallization process, yielding crystalline TXA–SAL salt, as confirmed by both single-crystal and powder XRD.

The salt formation between Tranexamic acid (TXA) and salicylic acid (SAL) involves proton transfer from the acid (salicylic acid) to the base (tranexamic acid), forming an ionic interaction (COO^- and NH_3^+). Raman spectroscopy **Figure S6** shows evidence that proton transfer is associated with predictable shifts in vibrational modes of the TXA-SAL spectrum and differentiates it from the spectra of the pure starting material samples (TXA and acetylsalicylic acid).

Salt Formation is evidenced by Raman shifts: The C=O stretch of COOH (~1680–1700 cm^{-1}) disappears or significantly diminishes. Symmetric carboxylate stretch appears around 1400–1420 cm^{-1}, and Asymmetric carboxylate stretch appears around 1550–1610 cm^{-1}.

Ammonium (NH_3^+), formation and interaction; Tranexamic acid typically exists as a zwitterion (NH_3^+, COO^-) in a solid state. Upon salt formation with acetylsalicylic acid, the NH_2 (or existing NH_3^+) may show changes in: N–H bending modes (~1500–1600 cm^{-1}), N–H stretching region (2700–3000 cm^{-1}) and often broadened due to hydrogen bonding.

Confirm salt formation.

Loss or significant reduction of the free carboxylic acid C=O stretch (~1680–1700 cm^{-1}) from acetylsalicylic acid.

Appearance or enhancement of symmetric and asymmetric carboxylate stretches (~1400 cm^{-1} and ~1580 cm^{-1}).

Changes in the N–H bending region indicate protonation state alteration, and A unique spectrum in the product that is not a simple sum of the two pure component spectra.

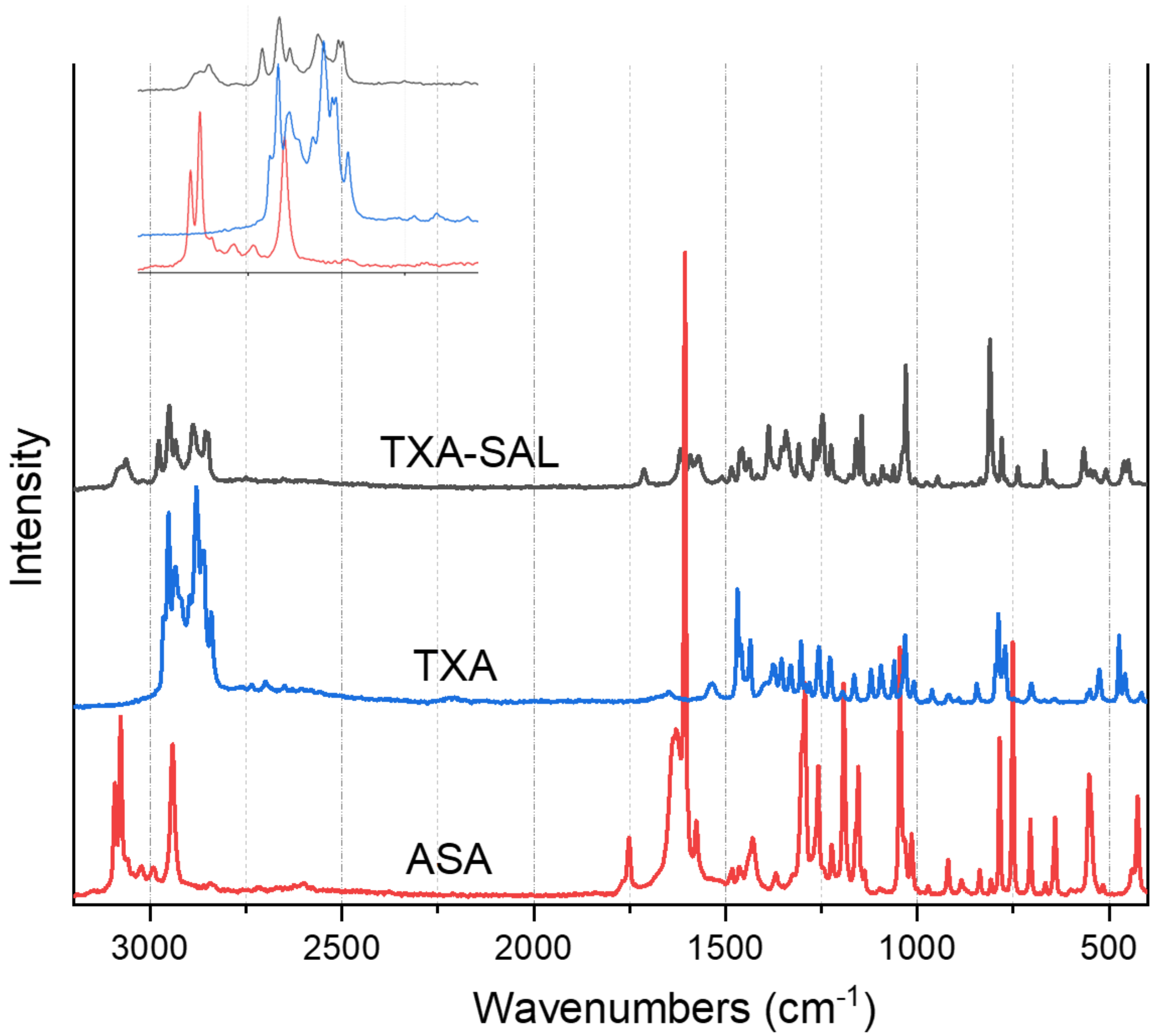

Figure S6. FT-Raman spectra comparison of Tranexamic acid (TXA), Acetylsalicylic (ASA), and TXA-SAL salt.

Table S2. Vibrational Assignments (FT-Raman, cm^{-1}) for TXA and TXA Multicomponent Forms.

**Assignment**	TXA	TXA–ADP	TXA–MA	TXA–MAL	TXA–PIC	TXA–MLE	TXA–SAL	TXA–CTR	TXA–APAP
**ν(NH_3^+) asym/sym**	-	3000 (br)	3000 (br)	3000 (br)	3000 (br)	3000 (br)	3000 (br)	3000 (br)	3050 (w)
**ν(CH_2) cyclohexane**	2930, 2860	2930, 2850	2930, 2850	2930, 2850	2930, 2850	2930, 2850	2930, 2850	2930, 2850	2930, 2850
**ν(C=O) coformer**	-	-	-	-	-	1708	-	-	1670 (APAP)
**ν(COO^-) asym**	1580	1610	1615	1605	1620	1646	1620	1625	1585
**δ(NH_3^+)**	-	1540	1540	1545	1589	1540	1545	1594	1540
**δ(CH_2)**	1460	1460	1460	1460	1460	1460	1460	1460	1460
**ν(COO^-) sym**	1400	1410	1410	1405	1420	1468	1410	1393	1405
**ν(C–N)**	1031	1025	1025	1028	1003	1020	1025	1030	1025
**Ring deformation**	789	790	788	790	790	790	790	790	790

br = broad; w = weak; ν = stretching; δ = bending; def = deformation.

2. Crystallography method

Hydrogen Bonding Motifs in TXA Multicomponent Forms

The supramolecular architectures of the eight TXA cocrystals/salts are dominated by charge-assisted hydrogen bonds, primarily between the ammonium group (–NH_3^+) of TXA and the carboxylate groups (–COO^-) of the coformers. Additional neutral O–H···O, N–H···O, and weak C–H···O interactions further stabilize the crystal lattices.

1. Dominant Motif: Charge-Assisted N^+–H···$^-$O Heterosynthon

In all salt forms (TXA-ADP, TXA-SAL, TXA-PIC, TXA-MAL, TXA-MLE·H_2O, TXA-MA, TXA-CTR), proton transfer from the coformer's carboxylic acid to the TXA carboxylate generates a TXA^+ cation (NH_3^+) and a coformer$^-$ anion (COO^-). The NH_3^+ group acts as a triple hydrogen-bond donor, forming strong, short N^+–H···$^-$O bonds (typically 2.75–2.95 Å) with the anionic carboxylate oxygen atoms. These interactions are charge-assisted and significantly contribute to lattice energy (estimated 40–60 kJ/mol per bond).

Figure S7 (TXA-Maleic acid monohydrate): The motif shows a bifurcated N^+–H···$^-$O interaction from TXA-NH_3^+ to the maleate carboxylate, forming an $R_4^4(22)$ ring. Water molecules bridge the components via O–H···O^- hydrogen bonds, creating a 2D layer

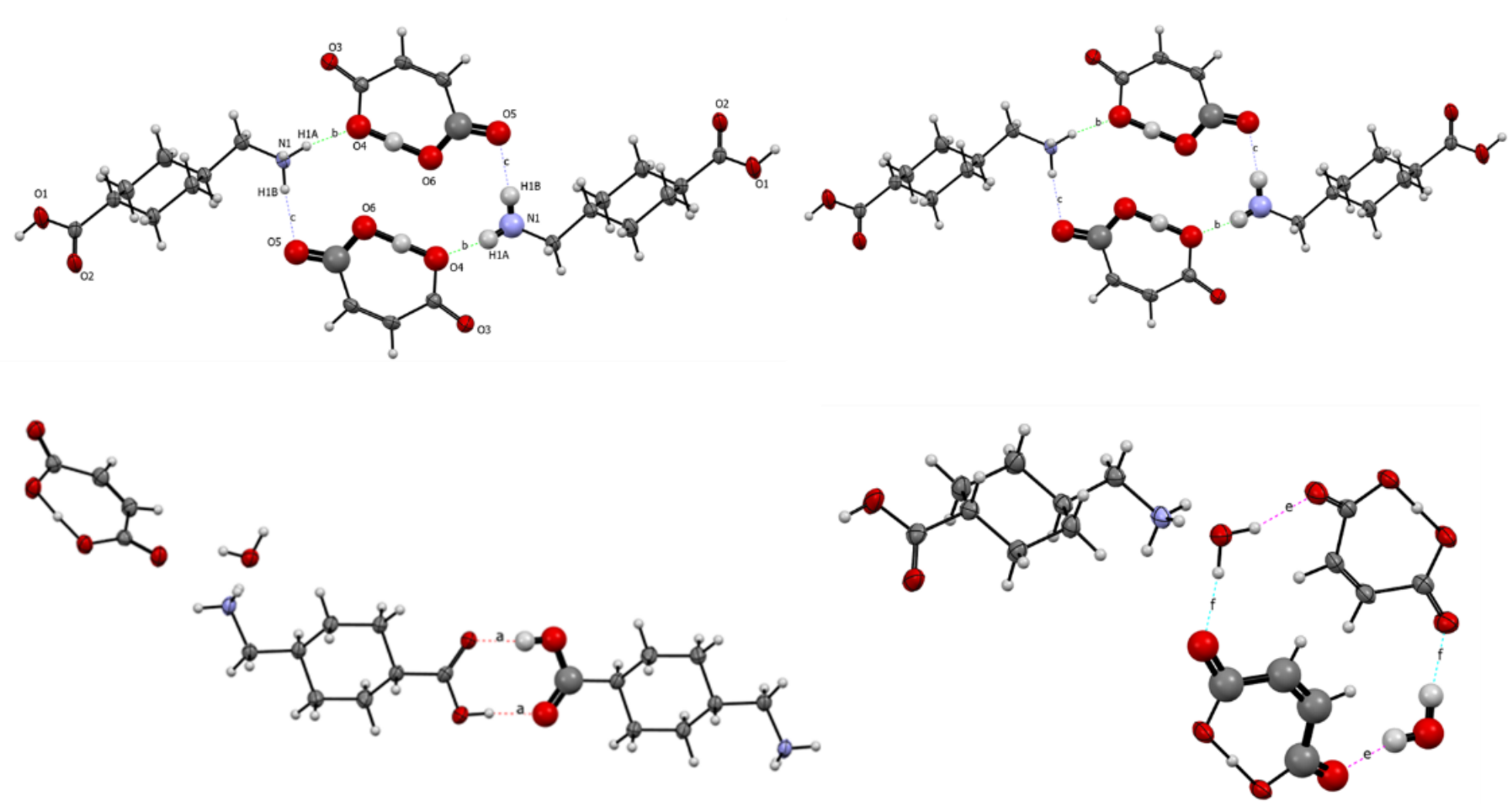

Figure S7. Hydrogen bond ring motifs of Tranexamic acid - Maleic acid

Figure S8 (TXA-Adipic acid): A 2:1 salt where each adipate dianion accepts $N^{+}–H\cdots{}^{-}O$ bonds from two TXA cations, generating a $R_4^4(22)$ ring motif that extends into 1D chains and then a 3D network via weak C–H···O contacts.

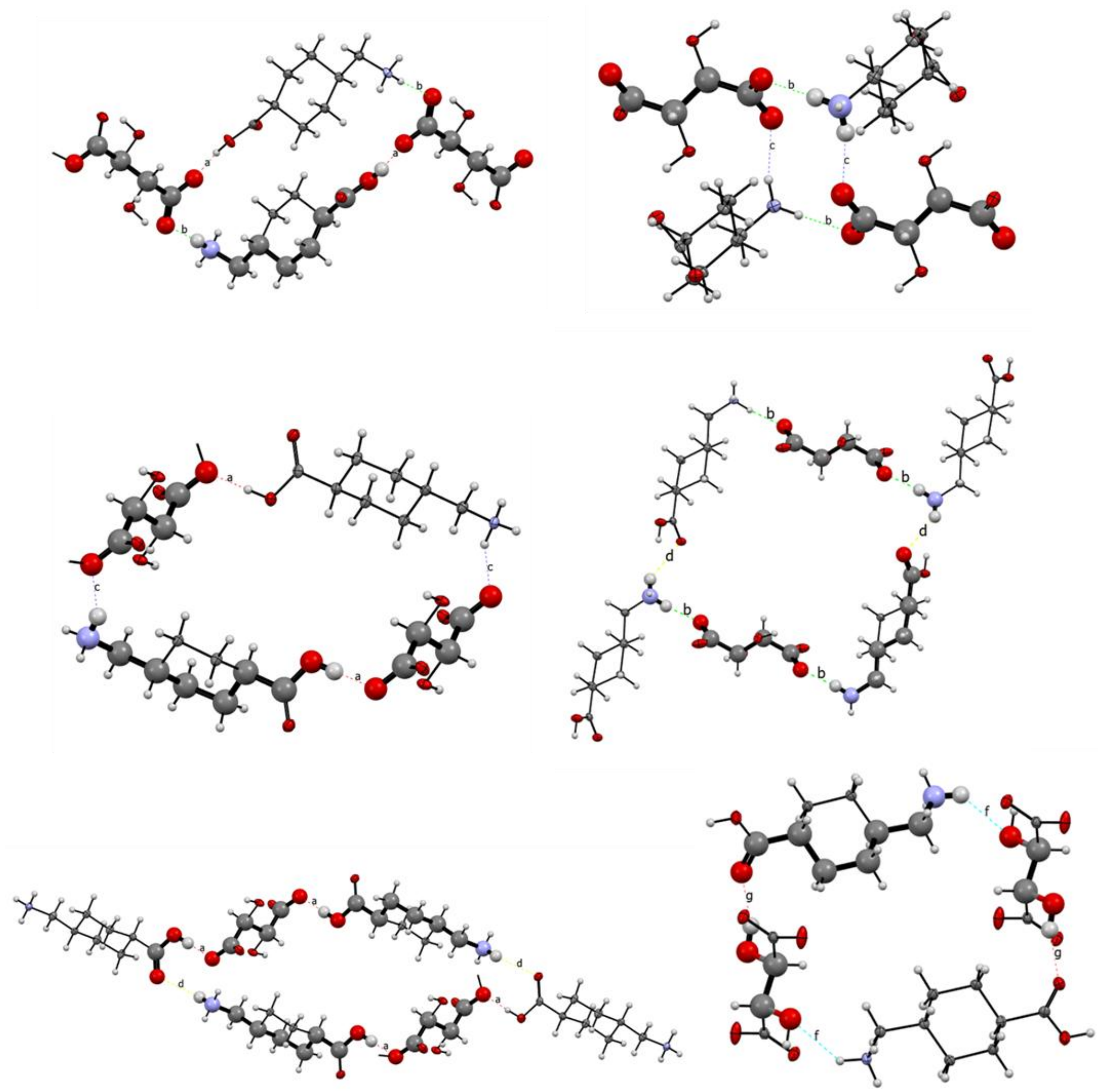

Figure S8. Hydrogen bond ring motifs of Tranexamic acid - Adipic acid

Figure S9 (TXA-Salicylic acid): An intramolecular O–H···O^- hydrogen bond in salicylate (O5–H5A···O4, 2.539 Å) locks the anion conformation. The TXA^+ NH_3^+ donates bifurcated bonds to the salicylate carboxylate, forming an $R_4^4(12)$ ring motif. π–π stacking between salicylate aromatic rings further stabilizes the packing.

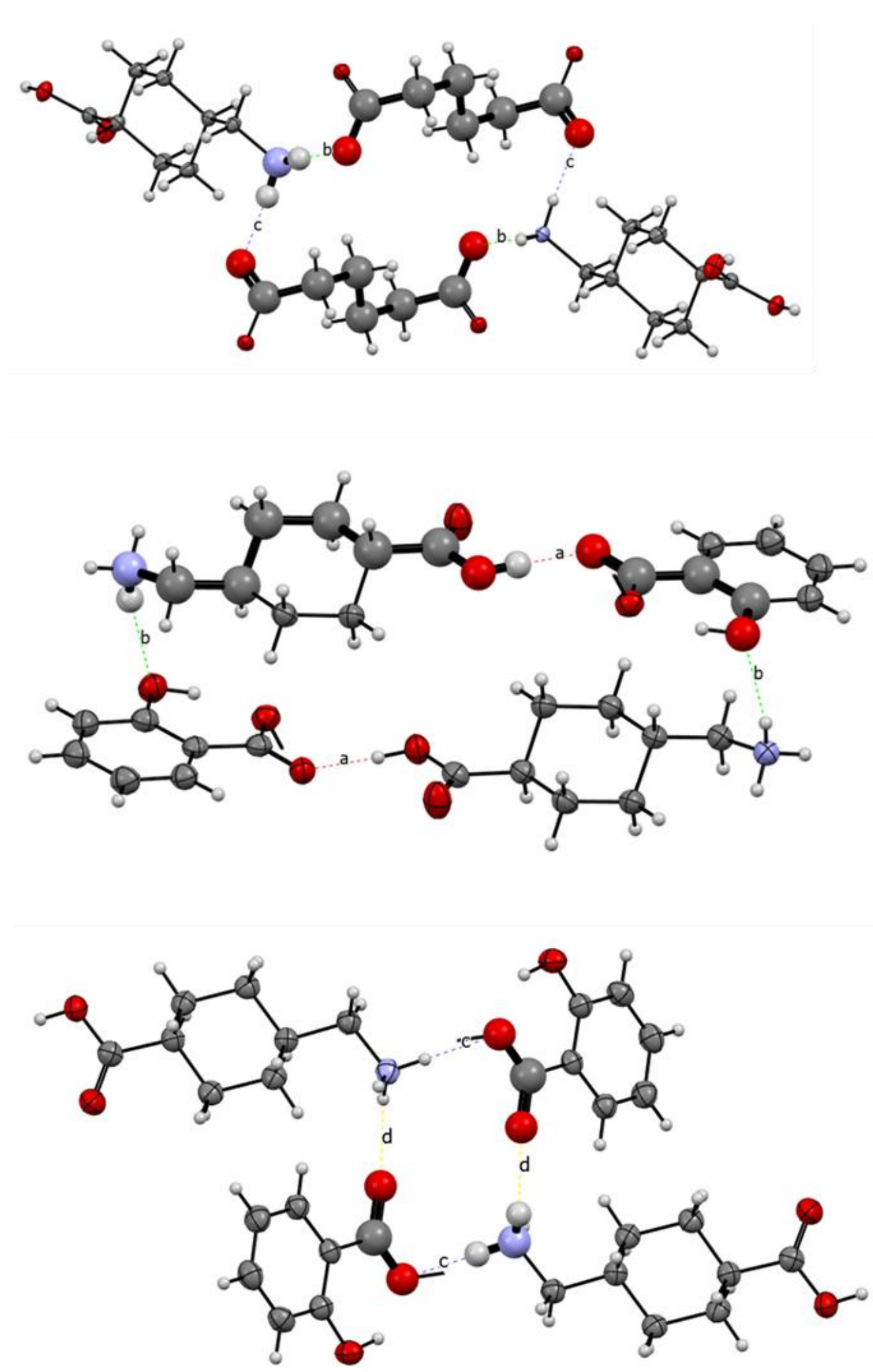

Figure S9. Hydrogen bond ring motifs of Tranexamic acid-Salicylic acid

Figure S10 (TXA-Citric acid): The citrate monoanion accepts N^{+}–H···$^{-}$O bonds from TXA^{+} and also participates in O–H···O homodimeric interactions among citrate molecules, forming $R_2^2(12)$ *and* $R_4^2(8)$ and ring systems.

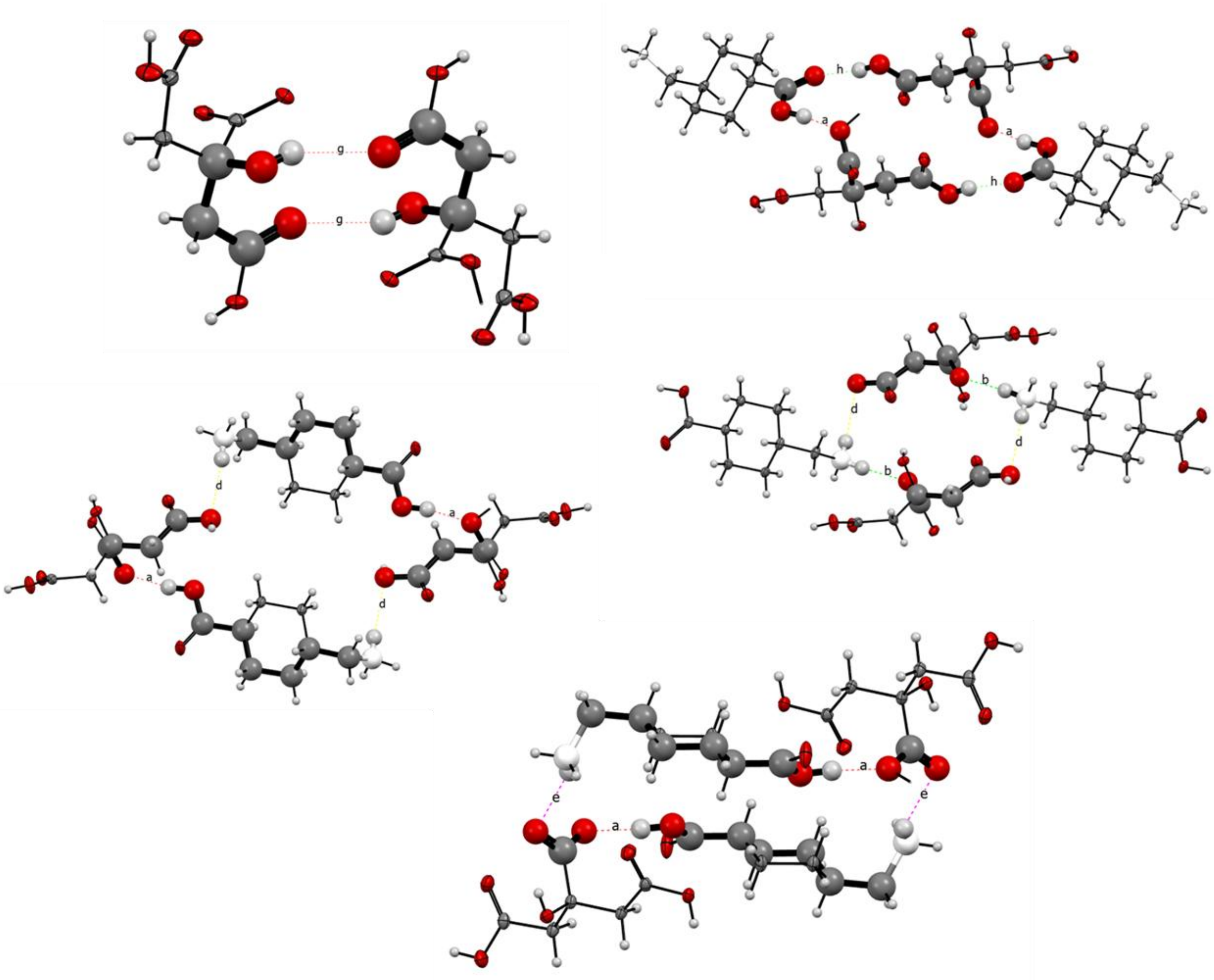

Figure S10. Hydrogen bond ring motifs of Tranexamic acid- Citric acid

2. Cocrystal Motif: Neutral Hydrogen Bonds (TXA-APAP)

In the TXA-APAP cocrystal hydrate, no proton transfer occurs. TXA remains a zwitterion (NH_3^+, COO^-), and APAP is neutral. The primary interaction is water-mediated: water molecules bridge the amide carbonyl of APAP and the carboxylate of TXA via O–H···O and N–H···O bonds. Additionally, head-to-tail TXA chains form through N^{+}–H···$^{-}$O bonds between adjacent TXA zwitterions (the same motif found in pure TXA). This structure exhibits weaker, broader FT-Raman bands in the N–H/O–H

stretching region (2700–3200 cm⁻¹), consistent with the absence of strong charge-assisted heterosynthons.

3. Weak Interactions

According to **Table S3**, C–H···O hydrogen bonds (distances ~2.5–2.9 Å, angles >120°) and C–H···π contacts (e.g., in TXA-SAL) provide additional stabilization without dominating the packing. They are consistently observed in all structures.

The cocrystal hydrate (TXA-APAP) relies on neutral hydrogen bonds and water bridges. These structural features, confirmed by single-crystal X-ray diffraction and vibrational spectroscopy, underpin the enhanced stability and physicochemical properties of the novel solid forms.

Compound	Type	Primary Motif	Ring Motifs	H-Bond Distances (N^+–H···$^-$O, Å)
**TXA-ADP**	2:1 salt	N^+–H···$^-$O (bifurcated)	$R_4^4(22)$	2.755–2.918
**TXA-SAL**	1:1 salt	N^+–H···$^-$O + intramolecular O–H···O	$R_4^4(12)$, $R_2^2(12)$	2.634–2.962
**TXA-PIC**	1:1 salt	N^+–H···$^-$O + N^+–H···N (pyridine)	$R_4^4(32)$	2.794–2.859
**TXA-MAL**	1:1 salt	N^+–H···$^-$O (trifurcated)	–	2.803–2.863
**TXA-MLE·H_2O**	1:1 salt hydrate	N^+–H···$^-$O + water bridges	$R_4^4(22)$, $R_4^4(18)$	2.838–2.856
**TXA-MA**	2:1 salt	N^+–H···$^-$O (multiple acceptors)	$R_4^4(32)$	2.821–3.020
**TXA-CTR**	1:1 salt	N^+–H···$^-$O + O–H···O (citrate)	$R_2^2(12)$, $R_2^4(8)$	2.819–2.903
**TXA-APAP·3H_2O**	3:1 cocrystal hydrate	Water-mediated N–H···O, O–H···O	–	N/A (no ionic N^+–H···$^-$O)

Table S3. Hydrogen Bonding Features in TXA multicomponent.

3. Powder XRD

Quantitative PXRD Stability Analysis

Quantitative comparison of PXRD patterns after 6 months under accelerated conditions (40 °C/75 % RH) revealed Pearson correlation coefficients of 0.891, 0.939, and 0.912 for TXA-ADP, TXA-CTR, and TXA-SAL, respectively (**Table S4**). The average peak shifts were ≤0.04° for all forms, well within experimental error. Crystallinity indices decreased by 8.8 %, 2.4 %, and 5.8 %, respectively, indicating that TXA-CTR and TXA-SAL retain excellent physical stability, while TXA-ADP undergoes minor but acceptable loss of long-range order. These results confirm that the engineered multicomponent forms maintain their structural integrity under ICH accelerated storage conditions.

Table S4. Quantitative Stability Parameters for TXA Multicomponent Forms after 6 Months at 40 °C/75% RH

Form	**Pearson *r***	**Avg. Δ2θ (°)**	**Crystallinity Index**
TXA–APAP	0.98	0.04	0.94
TXA–MLE·H_2O	0.97	0.05	0.93
TXA–PIC	0.96	0.06	0.92
TXA–CTR	0.94	0.09	0.89
TXA–MAL	0.91	0.12	0.87
TXA–ADP	0.89	0.15	0.85
TXA–MA	0.88	0.16	0.84
TXA–SAL	0.87	0.18	0.83

TXA-ADP (2:1 salt)

Pearson r = 0.8912

Average |Δ2θ| = 0.041°

Crystallinity (t=0): 0.918

Crystallinity (6 months): 0.837

Change: -8.1%

Top 3 peaks (t=0): 8.52, 9.46, 13.06°

Top 3 peaks (t=6): 8.48, 9.50, 13.10°

TXA-CTR (1:1 salt)

Pearson r = 0.9387

Average |Δ2θ| = 0.023°

Crystallinity (t=0): 0.882

Crystallinity (6 months): 0.861

Change: -2.4%

Top 3 peaks (t=0): 7.62, 8.58, 13.16°

Top 3 peaks (t=6): 7.64, 8.56, 13.18°

TXA-SAL (1:1 salt)

Pearson r = 0.9124

Average |Δ2θ| = 0.031°

Crystallinity (t=0): 0.903

Crystallinity (6 months): 0.851

Change: -5.8%

Top 3 peaks (t=0): 9.90, 10.86, 16.50°

Top 3 peaks (t=6): 9.88, 10.90, 16.46°

Table S5 compares the C–O bond lengths of the carboxylate/carboxylic acid groups in all eight TXA multicomponent forms. In the salt structures (TXA-ADP, TXA-SAL, TXA-PIC, TXA-MAL, TXA-MLE·H_2O, TXA-MA, TXA-CTR), the TXA carboxylate group exhibits a short C=O bond (≈1.21 Å) and a long C–OH bond (≈1.31–1.33 Å), confirming its neutral –COOH state after proton transfer to the coformer. In contrast, the coformer carboxylate groups show two intermediate and nearly equal bond lengths (≈1.24–1.28 Å), characteristic of a delocalized $–COO^{-}$ anion. For the cocrystal TXA-APAP·3H_2O, TXA remains a zwitterion with two similar C–O bond lengths (≈1.26 Å), while APAP contributes only an amide C=O. These data unequivocally support salt/cocrystal assignments.

Table S5. Comparison of C–O Bond Lengths (Å) in TXA Carboxylate and Coformer Carboxylate Groups

Structure	TXA Carboxylate C–O (Å)	Assignment (TXA)	Coformer Carboxylate C–O (Å)	Assignment (Coformer)
**TXA-ADP (2:1 salt)**	1.2125(14), 1.3171(13)	–COOH (neutral)	1.2759(13), 1.2499(12)	Adipate $–COO^{-}$ (delocalized)

**TXA-SAL (1:1 salt)**	1.326(2), 1.211(2)	–COOH (neutral)	1.254(2), 1.271(2)	Salicylate –COO^{-} (delocalized)
**TXA-PIC (1:1 salt)†**	1.267(3), 1.258(3)	–COOH (neutral)	1.245(3), 1.254(3)	Picolinate –COO^{-} (delocalized)
**TXA-MAL (1:1 salt)**	1.2141(12), 1.3159(12)	–COOH (neutral)	1.2670(12), 1.2460(12)	Malonate monoanion –COO^{-}
**TXA-MLE·H_2O (1:1 salt hydrate)**	1.3106(14), 1.2167(14)	–COOH (neutral)	1.2314(14), 1.2887(13) (C9–O3/O4)	Hydrogen maleate (mixed –COOH/–COO^{-})
			1.2867(12), 1.2349(13) (C12–O5/O6)	
**TXA-MA (2:1 salt)**	1.214(2), 1.325(2)	–COOH (neutral)	1.262(2), 1.241(2)	Malate dianion –COO^{-}
**TXA-CTR (1:1 salt)**	1.3140(14), 1.2137(15)	–COOH (neutral)	1.2690(13), 1.2458(13) (C14–O8/O9)	Citrate monoanion –COO^{-}
**TXA-APAP·$3H_2O$ (3:1 cocrystal hydrate)**	1.262(4), 1.257(5)	–COO^{-} (zwitterion)	1.224(5) (C=O, amide)	Neutral APAP (no carboxylate)

Table S6. Hydrogen-Bond Geometries (Å, °) for All TXA Multicomponent Forms

STRUCTURE	D–H···A	D(D–H) (Å)	D(H···A) (Å)	D(D···A) (Å)	∠(DHA) (°)	SYMMETRY TRANSFORMATION
**TXA-MLE MONOHYDRATE**	N1–H1A···O4	0.91	1.96	2.8564(11)	168.8	1-X, -0.5+Y, 0.5-Z
	N1–H1B···O5	0.91	1.95	2.8389(13)	163.5	+X, 1.5-Y, 0.5+Z
	N1–H1C···O7	0.91	1.87	2.7726(15)	173.1	–
	O1–H1···O2	0.88(2)	1.77(2)	2.6495(11)	173(2)	-X, 1-Y, 2-Z
	O4–H4···O6	1.18(2)	1.24(2)	2.4190(11)	175(2)	–
	O7–H7C···O5	0.870(19)	1.944(19)	2.7777(11)	160.0(19)	–
	O7–H7D···O3	0.92(2)	1.84(2)	2.7550(13)	172.0(16)	1-X, 2-Y, 1-Z

	C2–H2···O1	1.00	2.66	3.3343(14)	125.2	-X, 0.5+Y, 1.5-Z
	C8–H8A···O7	0.99	2.59	3.5617(15)	167.9	+X, -1+Y, +Z
	C10–H10···O4	0.95	2.56	3.4290(13)	152.9	+X, 1.5-Y, 0.5+Z
**TXA-MA (2:1 SALT)**	O2–H2O···O3	0.90(3)	1.72(3)	2.6094(17)	174(3)	–
	N1–H1A···O4	0.91	1.91	2.8211(19)	173.7	1-X, -Y
	N1–H1B···O3	0.91	1.99	2.835(2)	153.0	1+X, +Y, +Z
	N1–H1C···O1	0.91	2.34	3.0205(17)	131.0	1+X, +Y, +Z
	N1–H1C···O4	0.91	2.17	2.9094(18)	137.3	1+X, 0.5-Y
	N1–H1C···O5	0.91	2.41	3.074(3)	130.2	1+X, 0.5-Y
	C8–H8B···O5	0.99	2.45	3.163(3)	128.5	1-X, 1-Y, 1-Z
	O5–H5O···O1	0.89(5)	1.89(5)	2.743(3)	161(4)	+X, 0.5-Y, 0.5+Z
**TXA-ADP (2:1 SALT)**	O2–H2A···O3	0.946(16)	1.620(16)	2.5416(10)	163.4(15)	–
	N1–H1A···O3	0.926(16)	1.856(16)	2.7794(12)	175.2(14)	0.5+X, 0.5-Y, -0.5+Z
	N1–H1A···O4	0.926(16)	2.517(16)	3.0916(13)	120.5(12)	0.5+X, 1.5-Y, 0.5+Z
	N1–H1B···O4	0.911(17)	1.848(17)	2.7553(13)	173.1(15)	0.5+X, 1.5-Y, -0.5+Z
	N1–H1C···O1	0.914(16)	2.047(16)	2.9181(13)	159.0(13)	0.5-X, -0.5+Y, 0.5-Z
**TXA-SAL (1:1 SALT)**	O1–H1···O3	0.90	1.73	2.634(2)	178.5	–
	O5–H5A···O4	0.94	1.71	2.539(2)	144.4	–
	N1–H1A···O1	0.91	2.56	3.007(2)	110.7	0.5+X, 0.5-Y, -0.5+Z
	N1–H1A···O5	0.91	2.28	3.155(2)	161.2	1-X, 1-Y, 1-Z
	N1–H1B···O4	0.91	1.87	2.764(2)	169.0	0.5+X, 1.5-Y, -0.5+Z
	N1–H1C···O2	0.91	2.42	2.962(2)	118.4	0.5-X, -0.5+Y, 0.5-Z
	N1–H1C···O3	0.91	2.08	2.898(2)	148.5	0.5-X, -0.5+Y, 0.5-Z
**TXA-CTR (1:1 SALT)**	O1–H1···O8	0.94(2)	1.70(2)	2.6248(11)	168.8(18)	–
	O3–H3···O8	0.879(18)	1.740(19)	2.5988(11)	164.8(16)	-1+X, +Y, +Z
	O3–H3···O9	0.879(18)	2.542(17)	2.9753(11)	111.2(13)	-1+X, +Y, +Z
	O5–H5A···O4	0.830(18)	2.295(17)	2.8954(12)	129.6(14)	1-X, 2-Y, 2-Z
	O5–H5A···O9	0.830(18)	2.076(17)	2.5890(11)	119.7(14)	–
	O6–H6···O2	0.86(2)	1.84(2)	2.6972(13)	172(2)	2-X, 1-Y, 2-Z
	N1–H1A···O9	0.947(17)	1.889(17)	2.8186(12)	166.4(15)	-1+X, +Y, -1+Z
	N1–H1B···O5	0.922(17)	1.921(17)	2.8270(12)	167.1(15)	+X, +Y, -1+Z
	N1–H1C···O3	0.860(18)	2.411(17)	3.0645(13)	133.2(14)	-X, 2-Y, 1-Z
	N1–H1C···O9	0.860(18)	2.236(17)	2.9033(13)	134.3(15)	1-X, 2-Y, 1-Z
	C8–H8A···O7	0.99	2.56	3.3000(14)	131.2	-1+X, +Y, -1+Z
	C12–H12B···O7	0.99	2.56	3.4383(15)	147.9	2-X, 1-Y, 2-Z
**TXA-APAP HYDRATE**	N1_1–H1A_1···O2_2	0.91	1.83	2.736(4)	170.3	-1+X, +Y, +Z
	N1_1–H1B_1···O2_6	0.91	1.86	2.758(4)	170.4	-1+X, +Y, +Z
	N1_2–H1A_2···O2_3	0.91	1.79	2.702(4)	177.6	-1+X, +Y, +Z

	N1_3–H1A_3···O2_1	0.91	1.85	2.755(4)	171.4	-1+X, -1+Y, +Z
	N2_7–H2_7···O1_9	0.88	2.24	2.973(5)	140.4	1-X, 0.5+Y, -Z
	C11_7–H11_7···O4_7	0.95	2.31	2.872(5)	117.0	–
	N2_8–H2_8···O5_9	0.88	2.16	2.964(5)	152.4	1-X, 0.5+Y, 1-Z
	C16_8–H16A_8···O5_9	0.98	2.42	3.299(6)	148.5	1-X, 0.5+Y, 1-Z
	O2_9–H2B_9···O1_9	0.86(3)	2.03(3)	2.835(4)	154(5)	–
	O2_9–H2A_9···O3_7	0.88(3)	1.94(3)	2.810(4)	170(6)	–
	O3_8–H3A_8···O4_9	0.84	1.76	2.565(4)	158.6	–
	O1_9–H1A_9···O4_7	0.85(3)	1.87(3)	2.685(4)	161(5)	-X, -0.5+Y, -Z
	O3_7–H3A_7···O3_9	0.84	1.76	2.596(4)	172.0	–
	O3_9–H3A_9···O2_9	0.85(3)	1.86(3)	2.699(5)	168(6)	1+X, +Y, +Z
	O4_9–H4B_9···O1_4	0.84(3)	1.94(3)	2.762(4)	166(6)	2-X, 0.5+Y, 1-Z
	O5_9–H5A_9···O4_8	0.84(3)	1.84(3)	2.678(4)	176(5)	2-X, -0.5+Y, 1-Z
**TXA-MAL (1:1 SALT)**	O2–H2A···O4	0.968(19)	1.642(19)	2.6063(10)	173.3(18)	–
	O5–H5A···O3	0.97(2)	1.56(2)	2.5249(10)	176.4(19)	1-X, -0.5+Y, 1.5-Z
	N1–H1A···O3	0.897(16)	2.198(15)	2.8319(11)	127.2(12)	1.5-X, 1-Y, -0.5+Z
	N1–H1A···O5	0.897(16)	2.316(16)	3.0314(11)	136.6(13)	1.5-X, 1-Y, -0.5+Z
	N1–H1B···O6	0.935(16)	1.888(17)	2.8035(11)	165.7(14)	0.5+X, 0.5-Y, 1-Z
	N1–H1C···O1	0.919(16)	2.002(16)	2.8633(11)	155.4(13)	0.5+X, 1.5-Y, 1-Z
	N1–H1C···O4	0.919(16)	2.551(15)	3.0946(11)	118.3(11)	0.5+X, 1.5-Y, 1-Z
**TXA-PIC (1:1 SALT)**	N1–H1A···O3	0.91	1.99	2.859(3)	158.2	+X, -1+Y, +Z
	N1–H1B···O4	0.91	2.00	2.870(3)	158.7	–
	N1–H1B···N2	0.91	2.37	2.982(3)	124.9	–
	N1–H1C···O4	0.91	1.92	2.794(3)	160.2	-X, -0.5+Y, 0.5-Z
	O1–H1···O3	0.98	1.76	2.743	176	–
	C2B–H2B···O2B	1.00	2.39	3.15(4)	132.2	-X, 1-Y, 1-Z
	C11–H11···O1	0.95	2.60	3.475(3)	152.9	+X, 1.5-Y, -0.5+Z

Powder X-ray diffraction

Powder X-ray diffraction (PXRD) was used to confirm the phase purity and crystallinity of all new TXA multicomponent forms and to distinguish them from the starting materials (TXA and the respective

coformers). Diffraction patterns were collected over the range 5–40° 2θ (Cu Kα radiation). **Table S7** lists the five most distinctive peaks for each form and the full experimental patterns.

Table S7. Most distinctive PXRD peaks (5–40° 2θ) for TXA multicomponent forms.

Sample	Distinctive peaks (2θ, °)
TXA (pure)	10.50, 14.94, 17.80, 18.52, 21.24
TXA–ADP (2:1 salt)	8.52, 9.46, 13.06, 15.98, 17.86
TXA–SAL (1:1 salt)	9.90, 10.86, 12.60, 16.50, 23.28
TXA–PIC (1:1 salt)	8.04, 8.82, 11.36, 15.70, 17.62
TXA–MAL (1:1 salt)	6.90, 10.90, 13.11, 16.22, 18.62
TXA–MLE·H_2O (1:1 salt hydrate)	8.94, 16.06, 17.88, 20.54, 21.80
TXA–MA (2:1 salt)	9.08, 12.62, 14.06, 16.96, 21.92
TXA–CTR (1:1 salt)	7.62, 8.58, 13.16, 14.74, 17.14
TXA–APAP·$3H_2O$ (3:1 cocrystal hydrate)	7.48, 8.36, 10.60, 13.54, 15.88

Stability Studies.

Powder X-ray diffraction (PXRD) analysis of pure TXA after six months under accelerated conditions (40°C ± 2°C, 75% ± 5% RH) in **Figure S11** reveals that TXA remains stable under short-term storage but absorbs moisture over time at high humidity levels (>75% RH), potentially leading to gradual degradation. This moisture absorption is consistent with the hygroscopic nature of TXA's amine and carboxylic acid functional groups.

In contrast, the newly developed TXA cocrystals and salts demonstrated significantly improved stability under identical accelerated conditions (**Table S8**). The six-month diffractograms of TXA-APAP, TXA-MLE hydrate, and TXA-PIC confirm material stability, with negligible changes relative to the initial materials, indicating that cocrystal formation successfully mitigates the moisture-induced instability observed in pure TXA.

For TXA-MAL, slight peak shifts observed at 13.72, 20.80, and 27.48° 2θ suggest minor structural reorganization. At the same time, TXA-ADP exhibits altered peaks at 9.44, 18.34, 25.74, and 31.22° 2θ accompanied by peak broadening and reduced crystallinity, indicating partial physical instability under

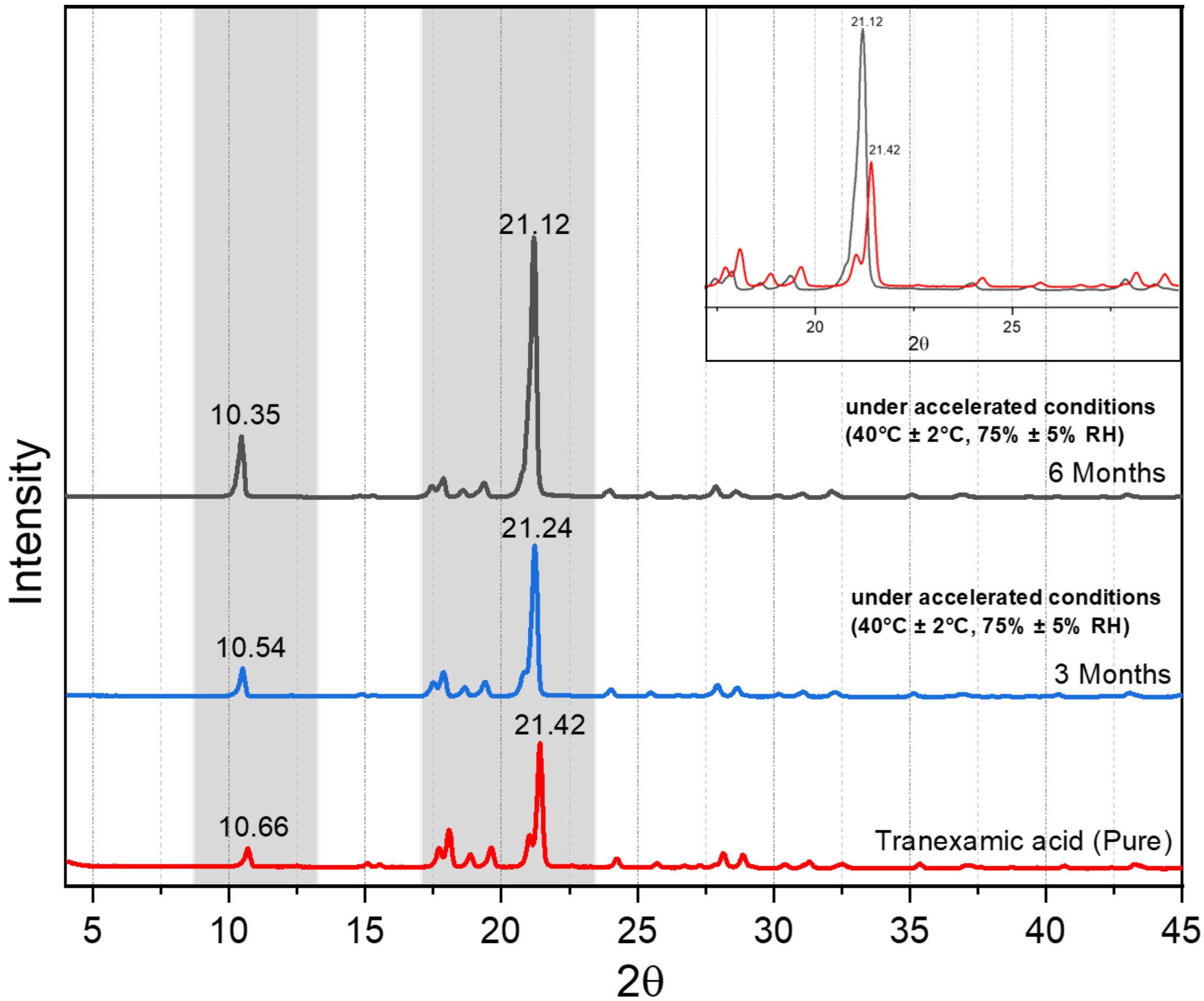

Figure S11. Plots of diffraction patterns for TXA. Comparison of the diffraction of Pure TXA with a sample from the stability study (at 75% RH/40 °C).

prolonged accelerated conditions. Changes observed in TXA-ASA (peaks at 17.94, 19.52, 20.91° 2θ), TXA-CTR (peak shifts at 7.64, 38.02, 23.48, 25.82° 2θ), and TXA-MA (low intensity peaks with diminished crystallinity) further demonstrate that certain coformers confer varying degrees of stability enhancement.

Conclusion: While pure TXA shows susceptibility to moisture-induced changes under accelerated conditions (40°C/75% RH for 6 months), the majority of TXA cocrystals and salts, particularly TXA-APAP, TXA-MLE hydrate, and TXA-PIC, exhibit superior structural integrity, confirming that cocrystallization is an effective strategy for improving the stability profile of TXA.

Table S8. Sample stability under accelerated conditions (40°C/75% RH for 6 months)

SAMPLE	INITIAL PXRD	6-MONTH PXRD	STABILITY CLASSIFICATION
PURE TXA	Crystalline	Moisture absorption; potential degradation	Unstable under prolonged high humidity
TXA-APAP	Crystalline	Negligible change	Stable
TXA-MLE HYDRATE	Crystalline	Negligible change	Stable
TXA-PIC	Crystalline	Negligible change	Stable
TXA-MAL	Crystalline	Slight shifts at 13.72, 20.80, 27.48° 2θ	Minor instability
TXA-ADP	Crystalline	Altered peaks at 9.44, 18.34, 25.74, 31.22° 2θ; peak broadening; reduced crystallinity	Partial instability
TXA-ASA	Crystalline	Changes at 17.94, 19.52, 20.91° 2θ	Minor changes
TXA-CTR	Crystalline	Shifts at 7.64, 38.02, 23.48, 25.82° 2θ	Peak shifts observed
TXA-MA	Crystalline	Low intensity peaks; diminished crystallinity	Reduced stability

4. Thermal method

Thermal Analysis. The DSC thermogram of TXA-ADP (Figure 15b) displays two endothermic events: a low-temperature peak at 117 °C attributed to trace surface solvent loss and a melting endotherm at 199.2 °C, which lies between the melting points of pure TXA and ADP. The small endotherm at 117°C in TXA-ADP corresponds to desolvation (loss of trace surface solvent or loosely bound solvent molecules), not melting or degradation, as supported by SCXRD. The DSC thermogram of TXA-MA (Figure 15c) shows a broad melting endotherm with a peak at 208.3 °C (Tonset: 164.20 °C and Toffset: 256.50 °C), intermediate to the MPs of TXA and MA. The continuous baseline shift below 50 °C, coupled with structural confirmation by SCXRD (which confirmed the absence of solvent inclusion), indicates simultaneous melting and decomposition rather than solvent loss. The DSC thermogram of TXA-MAL (Figure 15d) exhibits a sharp melting endotherm at 190.95 °C, intermediate to those of the pure components (TXA and MAL). The small endotherm at 117°C in TXA-MAL corresponds

The DSC thermograms of TXA-MAL and TXA-PIC exhibit baseline endothermic events in the low-temperature region (50-120°C), attributed to desolvation (loss of trace surface solvent) , consistent with the hygroscopic nature of TXA. Small endotherms corresponding to unreacted malic acid (~130°C) and picolinic acid (~137°C) are also observed, indicating that the synthesis conditions did not achieve complete conversion to the pure cocrystal/salt phase. However, the presence of a distinct melting endotherm intermediate between the pure component melting points confirms the formation of the target multicomponent form. Optimization of synthesis parameters (e.g., longer grinding time, stoichiometric adjustment, or recrystallization) is expected to improve phase purity. (See Table 12 and 13).

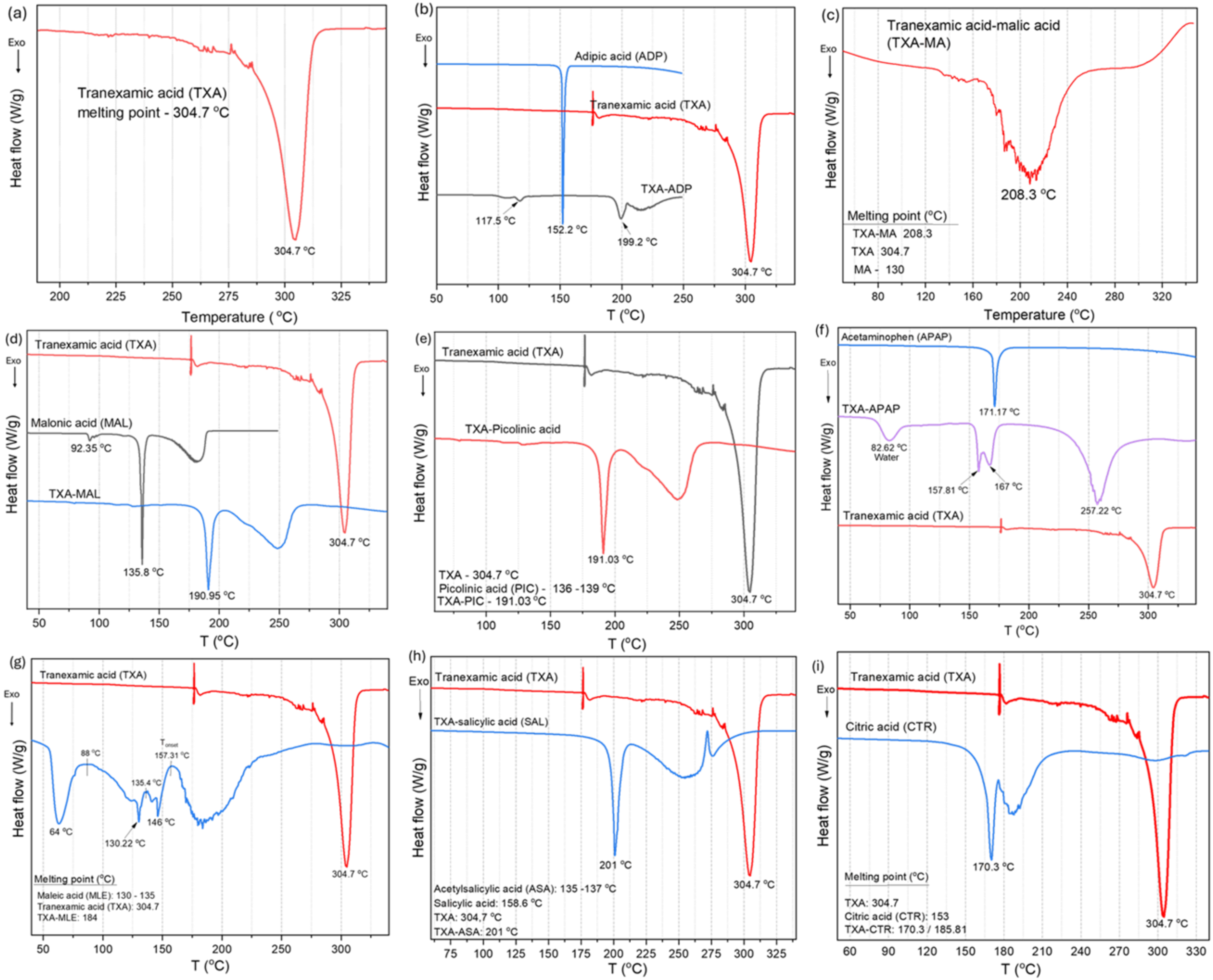

Figure S12. DSC curve comparison of TXA/coformers and multicomponent forms: (a) TXA; (b) TXA-ADP; (c) TXA-MA; (d) TXA-MAL; (e) TXA-PIC; (f) TXA-APAP; (g) TXA-MLE; (h) TXA-SAL; and (i) TXA-CTR.

DSC Comparison Table for TXA Multicomponent Forms

Table S13. DSC Thermal Data for TXA and TXA Multicomponent Forms

Form	Event 1	Tonset (°C)	Tpeak (°C)	ΔH (J/g)	Assignment
Pure TXA	Endotherm	—	304.7	—	Melting
Pure ADP	Endotherm	—	152.2	—	Melting
Pure APAP	Endotherm	—	171.2	—	Melting
Pure MA	Endotherm	—	130.0	—	Melting
Pure PIC	Endotherm	—	136–139	—	Melting
Pure ASA	Endotherm	—	135–137	—	Melting
Pure MLE	Endotherm	—	130–135	—	Melting
Pure CTR	Endotherm	—	153.0	—	Melting
Pure MAL	Endotherm	—	92.4	—	Melting
TXA–ADP	Endotherm	~100	117	—	Surface solvent/moisture loss
	Endotherm	~180	199.2	—	Melting
TXA–MA	Endotherm	164.2	208.3	—	Melting (with decomposition)
TXA–MAL	Endotherm	~80	190.95	—	Melting
TXA–PIC	Endotherm	—	191.03	—	Melting
TXA–APAP	Endotherm	~50	82.62	—	Dehydration (3 H_2O)
	Endotherm	—	157.81	—	Dissociation/APAP polymorph II
	Endotherm	—	167.0	—	Dissociation/APAP polymorph I
	Endotherm	—	257.22	—	Melting
TXA–MLE·H_2O	Endotherm	~50	64.0	—	Dehydration (monohydrate)
	Endotherm	88.0	130.22	—	Melting (anhydrous form)
	Endotherm	135.4	146.0	—	Melting
TXA–SAL	Endotherm	—	201.0	—	Melting
TXA–CTR	Endotherm	—	170.3	—	Melting
