# Supplementary material for: Antifibrinolytic Agent: Synthesis and Characterization of Cocrystal and Salts of Tranexamic Acid
Source: ACS Omega. 2026 Oct 22;11(43):66176–200. doi: 10.1021/acsomega.6c03598 (PMC13737987; doi:10.1021/acsomega.6c03598)
Supplement: Supplementary file 3 [file ao6c03598_si_002.zip › report_TXA_ASA_0m_complete-finalcif.docx]

# Structure Tables

A colourless, fragment-shaped crystal was mounted on the goniometer. Data for TXA_ASA_0m were collected from a shock-cooled single crystal at 150(2) K on a Bruker AXS D8 Quest four circle diffractometer with an I-mu-S 3.0 microsource X-ray tube using a HELIOS multilayer Montel optics as monochromator and a PhotonIII_C14 charge-integrating and photon counting pixel array detector. The diffractometer used CuKα radiation (λ = 1.54178 Å). All data were integrated with SAINT V8.40B and a multi-scan absorption correction using SADABS 2016/2 was applied.^[1,2]^ The structure was solved by dual methods with SHELXT and refined by full-matrix least-squares methods against *F*^2^ using SHELXL-2019/2.^[3,4]^ All non-hydrogen atoms were refined with anisotropic displacement parameters. All C-bound hydrogen atoms were refined isotropic on calculated positions using a riding model with their *U*_iso_ values constrained to 1.5 times the *U*_eq_ of their pivot atoms for terminal sp^3^ carbon atoms and 1.2 times for all other carbon atoms. Crystallographic data for the structures reported in this paper have been deposited with the Cambridge Crystallographic Data Centre.^[5]^ CCDC ?????? contain the supplementary crystallographic data for this paper. These data can be obtained free of charge from The Cambridge Crystallographic Data Centre via www.ccdc.cam.ac.uk/‌structures. This report and the CIF file were generated using FinalCif.^[6]^

## Table 1 Crystal data and structure refinement for TXA_ASA_0m

| CCDC number |  |
| --- | --- |
| Empirical formula | C15H21NO5 |
| Formula weight | 295.33 |
| Temperature [K] | 150(2) |
| Crystal system | monoclinic |
| Space group (number) | $P2_{1}/n$ (14) |
| *a* [Å] | 9.0893(8) |
| *b* [Å] | 13.9859(12) |
| *c* [Å] | 11.9845(11) |
| α [°] | 90 |
| β [°] | 105.219(5) |
| γ [°] | 90 |
| Volume [Å^3^] | 1470.1(2) |
| *Z* | 4 |
| *ρ*_calc_ [gcm^−3^] | 1.334 |
| *μ* [mm^−1^] | 0.831 |
| *F*(000) | 632 |
| Crystal size [mm^3^] | 0.090×0.130×0.150 |
| Crystal colour | colourless |
| Crystal shape | fragment |
| Radiation | CuKα (λ=1.54178 Å) |
| 2θ range [°] | 9.92 to 159.77 (0.78 Å) |
| Index ranges | −11 ≤ h ≤ 11 −17 ≤ k ≤ 17 −15 ≤ l ≤ 14 |
| Reflections collected | 17320 |
| Independent reflections | 3196  *R*_int_ = 0.0865 *R*_sigma_ = 0.0842 |
| Completeness to  θ = 67.679° | 100.0 % |
| Data / Restraints / Parameters | 3196 / 0 / 195 |
| Absorption correction T_min_/T_max_ (method) | 0.5662 / 0.7543  (multi-scan) |
| Goodness-of-fit on *F*^2^ | 1.060 |
| Final *R* indexes  [*I*≥2σ(*I*)] | *R*_1_ = 0.0549 w*R*_2_ = 0.1441 |
| Final *R* indexes  [all data] | *R*_1_ = 0.0716 w*R*_2_ = 0.1585 |
| Largest peak/hole [eÅ^−3^] | 0.28/−0.21 |

## Table 1 Atomic coordinates and *U_eq_* [Å^2^] for TXA_ASA_0m

| **Atom** | ***x*** | ***y*** | ***z*** | ***U*_eq_** |
| --- | --- | --- | --- | --- |
| O1 | 0.27106(16) | 0.54394(11) | 0.51195(12) | 0.0402(3) |
| H1 | 0.213(4) | 0.492(3) | 0.5126(3) | 0.060 |
| O2 | 0.1767(2) | 0.52830(12) | 0.32240(13) | 0.0530(4) |
| O3 | 0.10597(15) | 0.38944(10) | 0.51390(11) | 0.0372(3) |
| O4 | 0.18755(15) | 0.37978(10) | 0.70540(11) | 0.0381(3) |
| O5 | 0.14045(16) | 0.23014(11) | 0.81088(11) | 0.0417(3) |
| H5A | 0.171(3) | 0.294(3) | 0.8028(6) | 0.062 |
| N1 | 0.61721(17) | 0.92784(12) | 0.20312(14) | 0.0358(4) |
| H1A | 0.682765 | 0.887022 | 0.181593 | 0.054 |
| H1B | 0.652664 | 0.988745 | 0.203837 | 0.054 |
| H1C | 0.523704 | 0.923585 | 0.151976 | 0.054 |
| C1 | 0.2523(2) | 0.57318(14) | 0.40387(16) | 0.0349(4) |
| C2 | 0.32705(19) | 0.66810(13) | 0.39376(15) | 0.0328(4) |
| H2 | 0.275210 | 0.717095 | 0.430920 | 0.039 |
| C3 | 0.3037(2) | 0.69812(14) | 0.26789(16) | 0.0363(4) |
| H3A | 0.353462 | 0.651093 | 0.227939 | 0.044 |
| H3B | 0.193333 | 0.698648 | 0.228614 | 0.044 |
| C4 | 0.3704(2) | 0.79717(14) | 0.25971(16) | 0.0352(4) |
| H4A | 0.312885 | 0.845068 | 0.292197 | 0.042 |
| H4B | 0.358570 | 0.813268 | 0.177380 | 0.042 |
| C5 | 0.53970(19) | 0.80229(13) | 0.32507(15) | 0.0314(4) |
| H5 | 0.597037 | 0.757164 | 0.287206 | 0.038 |
| C6 | 0.56186(19) | 0.77031(14) | 0.45047(15) | 0.0338(4) |
| H6A | 0.510781 | 0.816417 | 0.490607 | 0.041 |
| H6B | 0.672034 | 0.770490 | 0.490360 | 0.041 |
| C7 | 0.4971(2) | 0.67023(14) | 0.45825(16) | 0.0348(4) |
| H7A | 0.510126 | 0.652892 | 0.540359 | 0.042 |
| H7B | 0.553203 | 0.622935 | 0.423669 | 0.042 |
| C8 | 0.6049(2) | 0.90181(13) | 0.32132(16) | 0.0341(4) |
| H8A | 0.538810 | 0.948908 | 0.346417 | 0.041 |
| H8B | 0.707294 | 0.905040 | 0.376279 | 0.041 |
| C9 | 0.11903(18) | 0.34560(13) | 0.60727(15) | 0.0323(4) |
| C10 | 0.05001(18) | 0.24789(13) | 0.60437(15) | 0.0312(4) |
| C11 | 0.0604(2) | 0.19653(14) | 0.70668(16) | 0.0342(4) |
| C12 | −0.0081(2) | 0.10685(15) | 0.70301(18) | 0.0390(4) |
| H12 | −0.000913 | 0.072377 | 0.772538 | 0.047 |
| C13 | −0.0862(2) | 0.06838(15) | 0.59816(19) | 0.0421(5) |
| H13 | −0.133932 | 0.007724 | 0.596105 | 0.051 |
| C14 | −0.0960(2) | 0.11735(15) | 0.49547(18) | 0.0421(5) |
| H14 | −0.148948 | 0.090095 | 0.423527 | 0.051 |
| C15 | −0.0276(2) | 0.20647(14) | 0.49914(16) | 0.0359(4) |
| H15 | −0.033558 | 0.239852 | 0.429047 | 0.043 |

*U*_eq_ is defined as 1/3 of the trace of the orthogonalized *U_ij_* tensor.

## Table 1 Anisotropic displacement parameters [Å^2^] for TXA_ASA_0m. The anisotropic displacement factor exponent takes the form: −2π^2^[ *h*^2^*(a*)*^2^*U*_11_ *+ k*^2^*(b*)*^2^*U*_22_ + … + *2hka*b*U*_12_ ]

| **Atom** | ***U*_11_** | ***U*_22_** | ***U*_33_** | ***U*_23_** | ***U*_13_** | ***U*_12_** |
| --- | --- | --- | --- | --- | --- | --- |
| O1 | 0.0415(7) | 0.0401(8) | 0.0387(7) | 0.0022(6) | 0.0099(5) | −0.0091(6) |
| O2 | 0.0629(10) | 0.0512(9) | 0.0413(8) | −0.0022(6) | 0.0074(6) | −0.0211(7) |
| O3 | 0.0360(6) | 0.0366(7) | 0.0376(7) | 0.0037(5) | 0.0069(5) | −0.0037(5) |
| O4 | 0.0384(7) | 0.0356(7) | 0.0373(7) | −0.0010(5) | 0.0045(5) | 0.0005(5) |
| O5 | 0.0442(7) | 0.0440(8) | 0.0343(7) | 0.0028(6) | 0.0058(5) | −0.0026(6) |
| N1 | 0.0324(7) | 0.0351(8) | 0.0380(8) | 0.0039(6) | 0.0056(6) | −0.0025(6) |
| C1 | 0.0295(8) | 0.0385(10) | 0.0376(9) | −0.0015(7) | 0.0102(7) | −0.0005(7) |
| C2 | 0.0282(8) | 0.0334(9) | 0.0368(9) | 0.0002(7) | 0.0088(6) | −0.0005(6) |
| C3 | 0.0290(8) | 0.0414(10) | 0.0369(9) | 0.0006(7) | 0.0055(6) | −0.0034(7) |
| C4 | 0.0286(8) | 0.0397(10) | 0.0363(9) | 0.0039(7) | 0.0065(6) | −0.0001(7) |
| C5 | 0.0281(8) | 0.0318(9) | 0.0344(8) | 0.0013(7) | 0.0086(6) | 0.0009(6) |
| C6 | 0.0280(8) | 0.0390(10) | 0.0334(9) | 0.0003(7) | 0.0063(6) | −0.0013(7) |
| C7 | 0.0295(8) | 0.0385(10) | 0.0351(9) | 0.0050(7) | 0.0061(6) | 0.0001(7) |
| C8 | 0.0317(8) | 0.0345(9) | 0.0353(9) | 0.0002(7) | 0.0076(6) | −0.0010(7) |
| C9 | 0.0258(7) | 0.0335(9) | 0.0375(9) | 0.0007(7) | 0.0078(6) | 0.0045(6) |
| C10 | 0.0248(7) | 0.0311(9) | 0.0379(9) | 0.0010(7) | 0.0086(6) | 0.0041(6) |
| C11 | 0.0291(8) | 0.0377(10) | 0.0360(9) | 0.0002(7) | 0.0088(6) | 0.0032(7) |
| C12 | 0.0365(9) | 0.0388(10) | 0.0432(10) | 0.0065(8) | 0.0133(7) | 0.0020(7) |
| C13 | 0.0375(9) | 0.0346(10) | 0.0535(12) | 0.0005(8) | 0.0106(8) | −0.0013(7) |
| C14 | 0.0401(10) | 0.0396(10) | 0.0435(11) | −0.0041(8) | 0.0053(8) | −0.0016(8) |
| C15 | 0.0348(9) | 0.0349(9) | 0.0371(9) | 0.0000(7) | 0.0081(7) | 0.0015(7) |

## Table 1 Bond lengths and angles for TXA_ASA_0m

| **Atom–Atom** | **Length [Å]** |
| --- | --- |
| O1–C1 | 1.326(2) |
| O2–C1 | 1.211(2) |
| O3–C9 | 1.254(2) |
| O4–C9 | 1.271(2) |
| O5–C11 | 1.354(2) |
| N1–C8 | 1.495(2) |
| C1–C2 | 1.510(3) |
| C2–C3 | 1.526(3) |
| C2–C7 | 1.536(2) |
| C3–C4 | 1.525(3) |
| C4–C5 | 1.535(2) |
| C5–C8 | 1.518(3) |
| C5–C6 | 1.530(2) |
| C6–C7 | 1.531(3) |
| C9–C10 | 1.500(3) |
| C10–C15 | 1.399(3) |
| C10–C11 | 1.403(3) |
| C11–C12 | 1.396(3) |
| C12–C13 | 1.380(3) |
| C13–C14 | 1.391(3) |
| C14–C15 | 1.389(3) |
|  |  |
| **Atom–Atom–Atom** | **Angle [°]** |
| O2–C1–O1 | 121.84(18) |
| O2–C1–C2 | 124.25(18) |
| O1–C1–C2 | 113.85(15) |
| C1–C2–C3 | 111.86(15) |
| C1–C2–C7 | 113.01(15) |
| C3–C2–C7 | 110.63(14) |
| C4–C3–C2 | 111.03(15) |
| C3–C4–C5 | 111.82(15) |
| C8–C5–C6 | 110.21(14) |
| C8–C5–C4 | 112.01(14) |
| C6–C5–C4 | 110.09(14) |
| C5–C6–C7 | 111.94(15) |
| C6–C7–C2 | 109.83(15) |
| N1–C8–C5 | 112.22(15) |
| O3–C9–O4 | 123.40(17) |
| O3–C9–C10 | 118.93(16) |
| O4–C9–C10 | 117.67(16) |
| C15–C10–C11 | 118.57(17) |
| C15–C10–C9 | 120.46(16) |
| C11–C10–C9 | 120.96(16) |
| O5–C11–C12 | 117.93(17) |
| O5–C11–C10 | 121.67(17) |
| C12–C11–C10 | 120.37(17) |
| C13–C12–C11 | 119.85(18) |
| C12–C13–C14 | 120.78(19) |
| C15–C14–C13 | 119.35(19) |
| C14–C15–C10 | 121.06(18) |

Bonds and angles to hydrogen atoms were omitted.

## Table 1 Torsion angles for TXA_ASA_0m

| **Atom–Atom–Atom–Atom** | **Torsion Angle [°]** |
| --- | --- |
| O2–C1–C2–C3 | −3.3(3) |
| O1–C1–C2–C3 | 179.42(15) |
| O2–C1–C2–C7 | −129.0(2) |
| O1–C1–C2–C7 | 53.8(2) |
| C1–C2–C3–C4 | 176.07(15) |
| C7–C2–C3–C4 | −57.0(2) |
| C2–C3–C4–C5 | 55.9(2) |
| C3–C4–C5–C8 | −177.66(15) |
| C3–C4–C5–C6 | −54.7(2) |
| C8–C5–C6–C7 | 179.81(14) |
| C4–C5–C6–C7 | 55.8(2) |
| C5–C6–C7–C2 | −57.34(19) |
| C1–C2–C7–C6 | −176.38(15) |
| C3–C2–C7–C6 | 57.3(2) |
| C6–C5–C8–N1 | 167.49(14) |
| C4–C5–C8–N1 | −69.57(19) |
| O3–C9–C10–C15 | 0.0(2) |
| O4–C9–C10–C15 | 179.68(16) |
| O3–C9–C10–C11 | −179.36(15) |
| O4–C9–C10–C11 | 0.3(2) |
| C15–C10–C11–O5 | 176.53(16) |
| C9–C10–C11–O5 | −4.1(3) |
| C15–C10–C11–C12 | −1.2(3) |
| C9–C10–C11–C12 | 178.16(16) |
| O5–C11–C12–C13 | −177.73(17) |
| C10–C11–C12–C13 | 0.1(3) |
| C11–C12–C13–C14 | 0.9(3) |
| C12–C13–C14–C15 | −0.8(3) |
| C13–C14–C15–C10 | −0.4(3) |
| C11–C10–C15–C14 | 1.4(3) |
| C9–C10–C15–C14 | −178.02(16) |

Bonds and angles to hydrogen atoms were omitted.

## Table 1 Hydrogen bonds for TXA_ASA_0m

| **D–H⋯A [Å]** | **d(D–H) [Å]** | **d(H⋯A) [Å]** | **d(D⋯A) [Å]** | **<(DHA) [°]** |
| --- | --- | --- | --- | --- |
| O1–H1⋯O3 | 0.90 | 1.73 | 2.634(2) | 178.5 |
| O5–H5A⋯O4 | 0.94 | 1.71 | 2.539(2) | 144.4 |
| N1–H1A⋯O1#1 | 0.91 | 2.56 | 3.007(2) | 110.7 |
| N1–H1A⋯O5#2 | 0.91 | 2.28 | 3.155(2) | 161.2 |
| N1–H1B⋯O4#1 | 0.91 | 1.87 | 2.764(2) | 169.0 |
| N1–H1C⋯O2#3 | 0.91 | 2.42 | 2.962(2) | 118.4 |
| N1–H1C⋯O3#3 | 0.91 | 2.08 | 2.898(2) | 148.5 |

Symmetry transformations used to generate equivalent atoms:

#1: 0.5+X, 1.5-Y, -0.5+Z; #2: 1-X, 1-Y, 1-Z; #3: 0.5-X, 0.5+Y, 0.5-Z;

## Bibliography

[1]	Bruker, SAINT, V8.40B, Bruker AXS Inc., Madison, Wisconsin, USA.

[2]	L. Krause, R. Herbst-Irmer, G. M. Sheldrick, D. Stalke, J. Appl. Cryst. 2015, 48, 3–10, doi:10.1107/S1600576714022985.

[3]	G. M. Sheldrick, Acta Cryst. 2015, A71, 3–8, doi:10.1107/S2053273314026370.

[4]	G. M. Sheldrick, Acta Cryst. 2015, C71, 3–8, doi:10.1107/S2053229614024218.

[5]	C. R. Groom, I. J. Bruno, M. P. Lightfoot, S. C. Ward, Acta Cryst. 2016, B72, 171–179, doi:10.1107/S2052520616003954.

[6]	D. Kratzert, FinalCif, V144, https://dkratzert.de/finalcif.html.
