# Supplementary material for: Antifibrinolytic Agent: Synthesis and Characterization of Cocrystal and Salts of Tranexamic Acid
Source: ACS Omega. 2026 Oct 22;11(43):66176–200. doi: 10.1021/acsomega.6c03598 (PMC13737987; doi:10.1021/acsomega.6c03598)
Supplement: Supplementary file 3 [file ao6c03598_si_002.zip › report_TXA_Citric_0m_complete-finalcif.docx]

# Structure Tables

A colourless, fragment-shaped crystal was mounted on the goniometer. Data for TXA_Citric_0m were collected from a shock-cooled single crystal at 150(2) K on a Bruker AXS D8 Quest four circle diffractometer with an I-mu-S 3.0 microsource X-ray tube using a HELIOS multilayer Montel optics as monochromator and a PhotonIII_C14 charge-integrating and photon counting pixel array detector. The diffractometer used Cu*K_α_* radiation (λ = 1.54178 Å). All data were integrated with SAINT V8.40B and a multi-scan absorption correction using SADABS 2016/2 was applied.^[1,2]^ The structure was solved by dual methods with SHELXT and refined by full-matrix least-squares methods against *F*^2^ using SHELXL-2019/2.^[3,4]^ All non-hydrogen atoms were refined with anisotropic displacement parameters. All hydrogen atoms were refined with isotropic displacement parameters. Some of their coordinates were refined freely and some on calculated positions using a riding model with their *U*_iso_ values constrained to 1.5 times the *U*_eq_ of their pivot atoms for terminal sp^3^ carbon atoms and 1.2 times for all other carbon atoms. Crystallographic data for the structures reported in this paper have been deposited with the Cambridge Crystallographic Data Centre.^[5]^ CCDC ?????? contain the supplementary crystallographic data for this paper. These data can be obtained free of charge from The Cambridge Crystallographic Data Centre via www.ccdc.cam.ac.uk/‌structures. This report and the CIF file were generated using FinalCif.^[6]^

## Table 1 Crystal data and structure refinement for TXA_Citric_0m

| CCDC number |  |
| --- | --- |
| Empirical formula | C_14_H_23_NO_9_ |
| Formula weight | 349.33 |
| Temperature [K] | 150(2) |
| Crystal system | triclinic |
| Space group (number) | $P\overline{1}$ (2) |
| *a* [Å] | 6.1653(2) |
| *b* [Å] | 11.1121(3) |
| *c* [Å] | 12.4264(4) |
| α [°] | 69.7695(9) |
| β [°] | 85.8749(10) |
| γ [°] | 80.3449(10) |
| Volume [Å^3^] | 787.40(4) |
| *Z* | 2 |
| *ρ*_calc_ [gcm^−3^] | 1.473 |
| *μ* [mm^−1^] | 1.062 |
| *F*(000) | 372 |
| Crystal size [mm^3^] | 0.140×0.230×0.260 |
| Crystal colour | colourless |
| Crystal shape | fragment |
| Radiation | Cu*K_α_* (λ=1.54178 Å) |
| 2θ range [°] | 7.58 to 160.32 (0.78 Å) |
| Index ranges | −7 ≤ h ≤ 7 −14 ≤ k ≤ 14 −15 ≤ l ≤ 15 |
| Reflections collected | 12844 |
| Independent reflections | 3344  *R*_int_ = 0.0449 *R*_sigma_ = 0.0421 |
| Completeness to  θ = 67.679° | 99.8 % |
| Data / Restraints / Parameters | 3344 / 0 / 238 |
| Absorption correction T_min_/T_max_ (method) | 0.6362 / 0.7543  (multi-scan) |
| Goodness-of-fit on *F*^2^ | 1.073 |
| Final *R* indexes  [*I*≥2σ(*I*)] | *R*_1_ = 0.0328 w*R*_2_ = 0.0877 |
| Final *R* indexes  [all data] | *R*_1_ = 0.0399 w*R*_2_ = 0.0894 |
| Largest peak/hole [eÅ^−3^] | 0.35/−0.25 |

## Table 1 Atomic coordinates and *U_eq_* [Å^2^] for TXA_Citric_0m

| **Atom** | ***x*** | ***y*** | ***z*** | ***U*_eq_** |
| --- | --- | --- | --- | --- |
| O1 | 0.68452(15) | 0.83632(9) | 0.64635(8) | 0.0276(2) |
| H1 | 0.764(3) | 0.8080(19) | 0.7144(18) | 0.041 |
| O2 | 0.81931(18) | 0.63841(10) | 0.64467(9) | 0.0377(3) |
| O3 | 0.18676(13) | 0.94127(8) | 0.76593(7) | 0.02017(18) |
| H3 | 0.107(3) | 0.8783(17) | 0.7895(15) | 0.030 |
| O4 | 0.45873(14) | 0.99951(8) | 0.83249(8) | 0.02498(19) |
| O5 | 0.53764(13) | 0.79379(8) | 1.07627(7) | 0.01840(17) |
| H5A | 0.598(3) | 0.8576(17) | 1.0683(14) | 0.028 |
| O6 | 0.83282(15) | 0.45815(9) | 1.21472(8) | 0.0296(2) |
| H6 | 0.953(3) | 0.430(2) | 1.2538(18) | 0.044 |
| O7 | 1.03691(14) | 0.60047(9) | 1.09869(8) | 0.0257(2) |
| O8 | 0.89318(12) | 0.78716(8) | 0.83729(7) | 0.01897(18) |
| O9 | 0.88241(12) | 0.88401(8) | 0.96807(6) | 0.01799(17) |
| N1 | 0.11319(16) | 0.84497(10) | 0.16805(8) | 0.0178(2) |
| H1A | 0.026(3) | 0.8463(15) | 0.1079(15) | 0.027 |
| H1B | 0.255(3) | 0.8177(15) | 0.1483(14) | 0.027 |
| H1C | 0.100(3) | 0.9234(17) | 0.1679(14) | 0.027 |
| C1 | 0.70095(18) | 0.74161(11) | 0.60436(9) | 0.0187(2) |
| C2 | 0.56095(18) | 0.77451(10) | 0.49976(9) | 0.0169(2) |
| H2 | 0.624271 | 0.842714 | 0.435522 | 0.020 |
| C3 | 0.56985(19) | 0.65603(11) | 0.46344(9) | 0.0197(2) |
| H3A | 0.514713 | 0.585370 | 0.527175 | 0.024 |
| H3B | 0.724363 | 0.625133 | 0.446662 | 0.024 |
| C4 | 0.43112(19) | 0.68817(11) | 0.35727(9) | 0.0189(2) |
| H4A | 0.491900 | 0.754843 | 0.292065 | 0.023 |
| H4B | 0.437313 | 0.609405 | 0.336330 | 0.023 |
| C5 | 0.19222(18) | 0.73840(11) | 0.37994(9) | 0.0187(2) |
| H5 | 0.140167 | 0.669698 | 0.448166 | 0.022 |
| C6 | 0.18179(18) | 0.85910(11) | 0.41432(10) | 0.0199(2) |
| H6A | 0.235584 | 0.929312 | 0.349592 | 0.024 |
| H6B | 0.027084 | 0.889645 | 0.431081 | 0.024 |
| C7 | 0.32154(18) | 0.82986(11) | 0.52008(9) | 0.0201(2) |
| H7A | 0.258166 | 0.766498 | 0.586974 | 0.024 |
| H7B | 0.318921 | 0.910533 | 0.537542 | 0.024 |
| C8 | 0.04213(18) | 0.75668(12) | 0.28093(9) | 0.0206(2) |
| H8A | 0.037218 | 0.670832 | 0.274496 | 0.025 |
| H8B | −0.108892 | 0.792003 | 0.299053 | 0.025 |
| C9 | 0.36005(17) | 0.91375(10) | 0.83241(9) | 0.0166(2) |
| C10 | 0.42619(17) | 0.77225(10) | 0.90189(9) | 0.0163(2) |
| H10A | 0.471781 | 0.723628 | 0.848551 | 0.020 |
| H10B | 0.295962 | 0.738438 | 0.945828 | 0.020 |
| C11 | 0.61362(17) | 0.74550(10) | 0.98586(9) | 0.0150(2) |
| C12 | 0.67524(18) | 0.59793(10) | 1.03942(10) | 0.0186(2) |
| H12A | 0.545624 | 0.561709 | 1.081971 | 0.022 |
| H12B | 0.711223 | 0.561168 | 0.977075 | 0.022 |
| C13 | 0.86717(18) | 0.55485(10) | 1.11993(9) | 0.0185(2) |
| C14 | 0.81307(16) | 0.81122(10) | 0.92594(9) | 0.0150(2) |

*U*_eq_ is defined as 1/3 of the trace of the orthogonalized *U_ij_* tensor.

## Table 1 Anisotropic displacement parameters [Å^2^] for TXA_Citric_0m. The anisotropic displacement factor exponent takes the form: −2π^2^[ *h*^2^*(a*)*^2^*U*_11_ *+ k*^2^*(b*)*^2^*U*_22_ + … + *2hka*b*U*_12_ ]

| **Atom** | ***U*_11_** | ***U*_22_** | ***U*_33_** | ***U*_23_** | ***U*_13_** | ***U*_12_** |
| --- | --- | --- | --- | --- | --- | --- |
| O1 | 0.0324(5) | 0.0304(5) | 0.0240(4) | −0.0141(4) | −0.0134(4) | 0.0005(4) |
| O2 | 0.0461(6) | 0.0301(5) | 0.0371(5) | −0.0133(4) | −0.0278(5) | 0.0092(4) |
| O3 | 0.0152(4) | 0.0241(4) | 0.0183(4) | −0.0036(3) | −0.0045(3) | −0.0012(3) |
| O4 | 0.0251(4) | 0.0196(4) | 0.0297(4) | −0.0058(3) | −0.0069(3) | −0.0046(3) |
| O5 | 0.0187(4) | 0.0220(4) | 0.0178(4) | −0.0104(3) | 0.0020(3) | −0.0051(3) |
| O6 | 0.0260(4) | 0.0285(5) | 0.0258(4) | 0.0025(4) | −0.0105(4) | −0.0024(4) |
| O7 | 0.0191(4) | 0.0291(4) | 0.0273(4) | −0.0069(4) | −0.0076(3) | −0.0025(3) |
| O8 | 0.0145(4) | 0.0288(4) | 0.0166(4) | −0.0113(3) | −0.0014(3) | −0.0032(3) |
| O9 | 0.0178(4) | 0.0218(4) | 0.0166(4) | −0.0078(3) | −0.0034(3) | −0.0052(3) |
| N1 | 0.0170(5) | 0.0223(5) | 0.0143(4) | −0.0066(4) | −0.0037(3) | −0.0010(4) |
| C1 | 0.0175(5) | 0.0232(5) | 0.0157(5) | −0.0056(4) | −0.0032(4) | −0.0047(4) |
| C2 | 0.0189(5) | 0.0181(5) | 0.0137(5) | −0.0046(4) | −0.0038(4) | −0.0029(4) |
| C3 | 0.0237(5) | 0.0181(5) | 0.0172(5) | −0.0061(4) | −0.0073(4) | 0.0006(4) |
| C4 | 0.0238(5) | 0.0187(5) | 0.0151(5) | −0.0068(4) | −0.0052(4) | −0.0018(4) |
| C5 | 0.0209(5) | 0.0232(5) | 0.0124(5) | −0.0044(4) | −0.0033(4) | −0.0067(4) |
| C6 | 0.0174(5) | 0.0256(5) | 0.0179(5) | −0.0095(4) | −0.0043(4) | −0.0002(4) |
| C7 | 0.0193(5) | 0.0265(5) | 0.0166(5) | −0.0103(4) | −0.0041(4) | −0.0006(4) |
| C8 | 0.0199(5) | 0.0279(6) | 0.0152(5) | −0.0061(4) | −0.0027(4) | −0.0086(4) |
| C9 | 0.0138(5) | 0.0204(5) | 0.0150(5) | −0.0061(4) | −0.0010(4) | −0.0001(4) |
| C10 | 0.0138(5) | 0.0179(5) | 0.0178(5) | −0.0062(4) | −0.0043(4) | −0.0017(4) |
| C11 | 0.0137(5) | 0.0174(5) | 0.0146(5) | −0.0066(4) | −0.0028(4) | −0.0007(4) |
| C12 | 0.0179(5) | 0.0171(5) | 0.0206(5) | −0.0056(4) | −0.0063(4) | −0.0010(4) |
| C13 | 0.0186(5) | 0.0173(5) | 0.0197(5) | −0.0079(4) | −0.0046(4) | 0.0019(4) |
| C14 | 0.0126(5) | 0.0181(5) | 0.0139(5) | −0.0047(4) | −0.0046(4) | −0.0004(4) |

## Table 1 Bond lengths and angles for TXA_Citric_0m

| **Atom–Atom** | **Length [Å]** |
| --- | --- |
| O1–C1 | 1.3140(14) |
| O2–C1 | 1.2137(15) |
| O3–C9 | 1.3267(13) |
| O4–C9 | 1.2139(14) |
| O5–C11 | 1.4235(12) |
| O6–C13 | 1.3236(14) |
| O7–C13 | 1.2114(15) |
| O8–C14 | 1.2690(13) |
| O9–C14 | 1.2458(13) |
| N1–C8 | 1.4920(14) |
| C1–C2 | 1.5150(14) |
| C2–C3 | 1.5241(14) |
| C2–C7 | 1.5395(15) |
| C3–C4 | 1.5276(14) |
| C4–C5 | 1.5276(16) |
| C5–C8 | 1.5294(14) |
| C5–C6 | 1.5327(15) |
| C6–C7 | 1.5326(14) |
| C9–C10 | 1.5102(15) |
| C10–C11 | 1.5350(13) |
| C11–C12 | 1.5326(14) |
| C11–C14 | 1.5470(14) |
| C12–C13 | 1.5097(14) |
|  |  |
| **Atom–Atom–Atom** | **Angle [°]** |
| O2–C1–O1 | 123.15(10) |
| O2–C1–C2 | 123.32(10) |
| O1–C1–C2 | 113.53(9) |
| C1–C2–C3 | 110.52(9) |
| C1–C2–C7 | 111.87(9) |
| C3–C2–C7 | 110.85(9) |
| C2–C3–C4 | 111.09(9) |
| C5–C4–C3 | 110.62(9) |
| C4–C5–C8 | 112.31(9) |
| C4–C5–C6 | 109.79(9) |
| C8–C5–C6 | 114.38(9) |
| C7–C6–C5 | 111.21(9) |
| C6–C7–C2 | 110.93(9) |
| N1–C8–C5 | 113.80(9) |
| O4–C9–O3 | 120.09(10) |
| O4–C9–C10 | 123.92(10) |
| O3–C9–C10 | 115.96(9) |
| C9–C10–C11 | 114.48(8) |
| O5–C11–C12 | 107.40(8) |
| O5–C11–C10 | 109.66(8) |
| C12–C11–C10 | 107.51(8) |
| O5–C11–C14 | 108.46(8) |
| C12–C11–C14 | 112.01(9) |
| C10–C11–C14 | 111.69(8) |
| C13–C12–C11 | 114.27(9) |
| O7–C13–O6 | 123.57(10) |
| O7–C13–C12 | 123.90(10) |
| O6–C13–C12 | 112.49(10) |
| O9–C14–O8 | 124.96(10) |
| O9–C14–C11 | 117.61(9) |
| O8–C14–C11 | 117.43(9) |

Bonds and angles to hydrogen atoms were omitted.

## Table 1 Torsion angles for TXA_Citric_0m

| **Atom–Atom–Atom–Atom** | **Torsion Angle [°]** |
| --- | --- |
| O2–C1–C2–C3 | 6.82(16) |
| O1–C1–C2–C3 | −174.14(10) |
| O2–C1–C2–C7 | 130.86(13) |
| O1–C1–C2–C7 | −50.10(13) |
| C1–C2–C3–C4 | −179.44(9) |
| C7–C2–C3–C4 | 55.93(12) |
| C2–C3–C4–C5 | −58.12(12) |
| C3–C4–C5–C8 | −173.29(9) |
| C3–C4–C5–C6 | 58.27(11) |
| C4–C5–C6–C7 | −57.42(12) |
| C8–C5–C6–C7 | 175.28(9) |
| C5–C6–C7–C2 | 55.78(12) |
| C1–C2–C7–C6 | −178.54(9) |
| C3–C2–C7–C6 | −54.68(12) |
| C4–C5–C8–N1 | −56.63(13) |
| C6–C5–C8–N1 | 69.36(13) |
| O4–C9–C10–C11 | 7.38(15) |
| O3–C9–C10–C11 | −174.67(9) |
| C9–C10–C11–O5 | 69.31(11) |
| C9–C10–C11–C12 | −174.23(9) |
| C9–C10–C11–C14 | −50.96(12) |
| O5–C11–C12–C13 | −65.26(11) |
| C10–C11–C12–C13 | 176.80(9) |
| C14–C11–C12–C13 | 53.73(12) |
| C11–C12–C13–O7 | −45.69(15) |
| C11–C12–C13–O6 | 136.69(10) |
| O5–C11–C14–O9 | 4.84(13) |
| C12–C11–C14–O9 | −113.52(10) |
| C10–C11–C14–O9 | 125.81(10) |
| O5–C11–C14–O8 | −174.92(8) |
| C12–C11–C14–O8 | 66.72(12) |
| C10–C11–C14–O8 | −53.95(12) |

Bonds and angles to hydrogen atoms were omitted.

## Table 1 Hydrogen bonds for TXA_Citric_0m

| **D–H⋯A [Å]** | **d(D–H) [Å]** | **d(H⋯A) [Å]** | **d(D⋯A) [Å]** | **<(DHA) [°]** |
| --- | --- | --- | --- | --- |
| O1–H1⋯O8 | 0.94(2) | 1.70(2) | 2.6248(11) | 168.8(18) |
| O3–H3⋯O8^#1^ | 0.879(18) | 1.740(19) | 2.5988(11) | 164.8(16) |
| O3–H3⋯O9^#1^ | 0.879(18) | 2.542(17) | 2.9753(11) | 111.2(13) |
| O5–H5A⋯O4^#2^ | 0.830(18) | 2.295(17) | 2.8954(12) | 129.6(14) |
| O5–H5A⋯O9 | 0.830(18) | 2.076(17) | 2.5890(11) | 119.7(14) |
| O6–H6⋯O2^#3^ | 0.86(2) | 1.84(2) | 2.6972(13) | 172(2) |
| N1–H1A⋯O9^#4^ | 0.947(17) | 1.889(17) | 2.8186(12) | 166.4(15) |
| N1–H1B⋯O5^#5^ | 0.922(17) | 1.921(17) | 2.8270(12) | 167.1(15) |
| N1–H1C⋯O3^#6^ | 0.860(18) | 2.411(17) | 3.0645(13) | 133.2(14) |
| N1–H1C⋯O9^#7^ | 0.860(18) | 2.236(17) | 2.9033(13) | 134.3(15) |
| C8–H8A⋯O7^#4^ | 0.99 | 2.56 | 3.3000(14) | 131.2 |
| C12–H12B⋯O7^#3^ | 0.99 | 2.56 | 3.4383(15) | 147.9 |

Symmetry transformations used to generate equivalent atoms:

#1: -1+X, +Y, +Z; #2: 1-X, 2-Y, 2-Z; #3: 2-X, 1-Y, 2-Z; #4: -1+X, +Y, -1+Z; #5: +X, +Y, -1+Z; #6: -X, 2-Y, 1-Z; #7: 1-X, 2-Y, 1-Z;

## Bibliography

[1]	Bruker, *SAINT*, *V8.40B*, Bruker AXS Inc., Madison, Wisconsin, USA.

[2]	L. Krause, R. Herbst-Irmer, G. M. Sheldrick, D. Stalke, *J. Appl. Cryst.* **2015**, *48*, 3–10, doi:10.1107/S1600576714022985.

[3]	G. M. Sheldrick, *Acta Cryst.* **2015**, *A71*, 3–8, doi:10.1107/S2053273314026370.

[4]	G. M. Sheldrick, *Acta Cryst.* **2015**, *C71*, 3–8, doi:10.1107/S2053229614024218.

[5]	C. R. Groom, I. J. Bruno, M. P. Lightfoot, S. C. Ward, *Acta Cryst.* **2016**, *B72*, 171–179, doi:10.1107/S2052520616003954.

[6]	D. Kratzert, *FinalCif*, *V144*, https://dkratzert.de/finalcif.html.
