# Supplementary material for: Antifibrinolytic Agent: Synthesis and Characterization of Cocrystal and Salts of Tranexamic Acid
Source: ACS Omega. 2026 Oct 22;11(43):66176–200. doi: 10.1021/acsomega.6c03598 (PMC13737987; doi:10.1021/acsomega.6c03598)
Supplement: Supplementary file 3 [file ao6c03598_si_002.zip › report_TXA_MA_0m_complete-finalcif.docx]

# Structure Tables

A colourless, block-shaped crystal was mounted on the goniometer. Data for TXA_MA_0m were collected from a shock-cooled single crystal at 150(2) K on a Bruker AXS D8 Quest four circle diffractometer with an I-mu-S 3.0 microsource X-ray tube using a HELIOS multilayer Montel optics as monochromator and a PhotonIII_C14 charge-integrating and photon counting pixel array detector. The diffractometer used Cu*K_α_* radiation (λ = 154.178 pm). All data were integrated with SAINT V8.40B and a multi-scan absorption correction using SADABS 2016/2 was applied.^[1,2]^ The structure was solved by dual methods with SHELXT and refined by full-matrix least-squares methods against *F*^2^ using SHELXL-2019/2.^[3,4]^ All non-hydrogen atoms were refined with anisotropic displacement parameters. All C-bound hydrogen atoms were refined with isotropic displacement parameters. Some of their coordinates were refined freely and some on calculated positions using a riding model with their *U*_iso_ values constrained to 1.5 times the *U*_eq_ of their pivot atoms for terminal sp^3^ carbon atoms and 1.2 times for all other carbon atoms. Disordered moieties were refined using bond lengths restraints and displacement parameter restraints. Crystallographic data for the structures reported in this paper have been deposited with the Cambridge Crystallographic Data Centre.^[5]^ CCDC ?????? contain the supplementary crystallographic data for this paper. These data can be obtained free of charge from The Cambridge Crystallographic Data Centre via www.ccdc.cam.ac.uk/‌structures. This report and the CIF file were generated using FinalCif.^[6]^

## Table 1 Crystal data and structure refinement for TXA_MA_0m

| CCDC number |  |
| --- | --- |
| Empirical formula | C_20_H_36_N_2_O_9_ |
| Formula weight | 448.51 |
| Temperature [K] | 150(2) |
| Crystal system | monoclinic |
| Space group (number) | $P2_{1}/c$ (14) |
| *a* [pm] | 1030.14(3) |
| *b* [pm] | 860.21(2) |
| *c* [pm] | 1282.14(3) |
| α [°] | 90 |
| β [°] | 109.3912(8) |
| γ [°] | 90 |
| Volume [nm^3^] | 1.1(5) |
| *Z* | 2 |
| *ρ*_calc_ [gcm^−3^] | 1.390 |
| *μ* [mm^−1^] | 0.915 |
| *F*(000) | 484 |
| Crystal size [mm^3^] | 0.110×0.130×0.160 |
| Crystal colour | colourless |
| Crystal shape | block |
| Radiation | Cu*K_α_* (λ=154.178 pm) |
| 2θ range [°] | 12.63 to 160.21 (78 pm) |
| Index ranges | −11 ≤ h ≤ 13 −10 ≤ k ≤ 10 −16 ≤ l ≤ 15 |
| Reflections collected | 22779 |
| Independent reflections | 2307  *R*_int_ = 0.0416 *R*_sigma_ = 0.0323 |
| Completeness to  θ = 67.679° | 99.0 % |
| Data / Restraints / Parameters | 2307 / 0 / 153 |
| Absorption correction T_min_/T_max_ (method) | 0.6642 / 0.7543  (multi-scan) |
| Goodness-of-fit on *F*^2^ | 1.222 |
| Final *R* indexes  [*I*≥2σ(*I*)] | *R*_1_ = 0.0473 w*R*_2_ = 0.1087 |
| Final *R* indexes  [all data] | *R*_1_ = 0.0494 w*R*_2_ = 0.1092 |
| Largest peak/hole [eÅ^−3^] | 0.32/−0.20 |
| Extinction coefficient | 0.0089(8) |

## Refinement details for TXA_MA_0m

The hydroxyl group is disordered over the two symmetry equivalent positions at C10 and its counterpart by inversion.

## Table 1 Atomic coordinates and *U_eq_* [pm^2^] for TXA_MA_0m

| **Atom** | ***x*** | ***y*** | ***z*** | ***U*_eq_** |
| --- | --- | --- | --- | --- |
| O1 | 0.25175(12) | 0.03767(14) | 0.33929(10) | 206(3) |
| O2 | 0.30475(13) | 0.26275(15) | 0.43113(11) | 239(3) |
| H2O | 0.232(3) | 0.248(3) | 0.453(2) | 360.0 |
| O3 | 0.10331(15) | 0.20980(17) | 0.50721(12) | 325(4) |
| O4 | 0.04118(14) | 0.26372(14) | 0.65252(10) | 227(3) |
| N1 | 0.94848(14) | 0.05943(17) | 0.30800(11) | 170(3) |
| H1A | 0.945880 | −0.044042 | 0.321955 | 250.0 |
| H1B | 0.970275 | 0.113279 | 0.372629 | 250.0 |
| H1C | 1.013143 | 0.077724 | 0.275396 | 250.0 |
| C1 | 0.32461(16) | 0.15176(19) | 0.36637(13) | 158(3) |
| C2 | 0.45009(16) | 0.18076(19) | 0.33237(13) | 155(3) |
| H2 | 0.462117 | 0.295613 | 0.327658 | 190.0 |
| C3 | 0.43224(16) | 0.1101(2) | 0.21935(13) | 184(3) |
| H3A | 0.352814 | 0.159714 | 0.163067 | 220.0 |
| H3B | 0.412296 | −0.002267 | 0.220874 | 220.0 |
| C4 | 0.56102(16) | 0.1321(2) | 0.18696(13) | 183(3) |
| H4A | 0.571522 | 0.243910 | 0.173348 | 220.0 |
| H4B | 0.548614 | 0.075776 | 0.116965 | 220.0 |
| C5 | 0.69291(16) | 0.07413(19) | 0.27486(13) | 154(3) |
| H5 | 0.686441 | −0.041281 | 0.281088 | 190.0 |
| C6 | 0.70786(16) | 0.1463(2) | 0.38746(13) | 164(3) |
| H6A | 0.789922 | 0.102250 | 0.444316 | 200.0 |
| H6B | 0.721421 | 0.259884 | 0.384340 | 200.0 |
| C7 | 0.58004(16) | 0.1144(2) | 0.41976(13) | 164(3) |
| H7A | 0.591671 | 0.162560 | 0.492437 | 200.0 |
| H7B | 0.569200 | 0.000915 | 0.426688 | 200.0 |
| C8 | 0.81147(16) | 0.1106(2) | 0.23287(13) | 179(3) |
| H8A | 0.793428 | 0.059818 | 0.160127 | 220.0 |
| H8B | 0.814171 | 0.224229 | 0.221508 | 220.0 |
| C9 | 0.07122(16) | 0.30353(19) | 0.57034(13) | 158(3) |
| C10 | 0.06854(17) | 0.4774(2) | 0.54349(13) | 196(4) |
| H10 | 0.146379 | 0.503671 | 0.516370 | 240.0 |
| H10A | 0.082042 | 0.538602 | 0.611685 | 240.0 |
| H10B | 0.144923 | 0.502137 | 0.515534 | 240.0 |
| O5 | 0.0769(3) | 0.5743(3) | 0.6424(2) | 195(5) |
| H5O | 0.144(5) | 0.530(6) | 0.697(4) | 290.0 |

*U*_eq_ is defined as 1/3 of the trace of the orthogonalized *U_ij_* tensor.

## Table 1 Anisotropic displacement parameters [pm^2^] for TXA_MA_0m. The anisotropic displacement factor exponent takes the form: −2π^2^[ *h*^2^*(a*)*^2^*U*_11_ *+ k*^2^*(b*)*^2^*U*_22_ + … + *2hka*b*U*_12_ ]

| **Atom** | ***U*_11_** | ***U*_22_** | ***U*_33_** | ***U*_23_** | ***U*_13_** | ***U*_12_** |
| --- | --- | --- | --- | --- | --- | --- |
| O1 | 176(6) | 231(6) | 225(6) | −5(5) | 84(5) | −28(5) |
| O2 | 225(6) | 255(7) | 295(7) | −69(5) | 166(5) | −32(5) |
| O3 | 384(8) | 319(8) | 388(8) | −195(6) | 283(7) | −153(6) |
| O4 | 326(7) | 187(6) | 223(6) | 10(5) | 166(5) | 5(5) |
| N1 | 138(6) | 198(7) | 196(7) | −17(5) | 86(5) | −14(5) |
| C1 | 136(7) | 190(8) | 138(7) | 37(6) | 33(6) | 41(6) |
| C2 | 141(7) | 162(8) | 170(7) | 11(6) | 64(6) | 10(6) |
| C3 | 128(7) | 262(9) | 149(7) | −2(6) | 29(6) | 5(6) |
| C4 | 164(7) | 249(9) | 141(7) | 10(6) | 55(6) | 9(6) |
| C5 | 141(7) | 150(7) | 183(7) | −2(6) | 69(6) | −8(6) |
| C6 | 132(7) | 201(8) | 160(7) | −3(6) | 50(6) | −11(6) |
| C7 | 141(7) | 218(8) | 134(7) | 14(6) | 46(6) | 1(6) |
| C8 | 164(7) | 215(8) | 170(7) | 8(6) | 69(6) | −6(6) |
| C9 | 124(7) | 188(8) | 162(7) | −17(6) | 49(6) | −14(6) |
| C10 | 195(8) | 212(9) | 169(8) | 21(6) | 41(7) | −46(6) |
| O5 | 249(12) | 158(11) | 150(11) | −22(9) | 29(9) | −12(9) |

## Table 1 Bond lengths and angles for TXA_MA_0m

| **Atom–Atom** | **Length [pm]** |
| --- | --- |
| O1–C1 | 121.4(2) |
| O2–C1 | 132.5(2) |
| O3–C9 | 126.2(2) |
| O4–C9 | 124.1(2) |
| N1–C8 | 148.7(2) |
| C1–C2 | 151.6(2) |
| C2–C3 | 152.5(2) |
| C2–C7 | 154.2(2) |
| C3–C4 | 152.8(2) |
| C4–C5 | 153.2(2) |
| C5–C8 | 152.2(2) |
| C5–C6 | 153.1(2) |
| C6–C7 | 153.0(2) |
| C9–C10 | 153.3(2) |
| C10–O5 | 149.6(3) |
| C10–C10^#1^ | 153.1(3) |
|  |  |
| **Atom–Atom–Atom** | **Angle [°]** |
| O1–C1–O2 | 123.52(14) |
| O1–C1–C2 | 123.78(15) |
| O2–C1–C2 | 112.68(14) |
| C1–C2–C3 | 111.25(13) |
| C1–C2–C7 | 110.34(12) |
| C3–C2–C7 | 110.16(13) |
| C2–C3–C4 | 111.56(13) |
| C3–C4–C5 | 113.44(13) |
| C8–C5–C6 | 114.11(13) |
| C8–C5–C4 | 107.01(13) |
| C6–C5–C4 | 110.41(13) |
| C7–C6–C5 | 110.87(13) |
| C6–C7–C2 | 110.76(13) |
| N1–C8–C5 | 114.23(13) |
| O4–C9–O3 | 124.10(16) |
| O4–C9–C10 | 117.96(14) |
| O3–C9–C10 | 117.93(15) |
| O5–C10–C10^#1^ | 104.82(19) |
| O5–C10–C9 | 111.21(15) |
| C10^#1^–C10–C9 | 110.78(17) |

Bonds and angles to hydrogen atoms were omitted.

Symmetry transformations used to generate equivalent atoms:

#1: -X, 1-Y, 1-Z;

## Table 1 Torsion angles for TXA_MA_0m

| **Atom–Atom–Atom–Atom** | **Torsion Angle [°]** |
| --- | --- |
| O1–C1–C2–C3 | 31.1(2) |
| O2–C1–C2–C3 | −150.41(14) |
| O1–C1–C2–C7 | −91.51(18) |
| O2–C1–C2–C7 | 87.01(17) |
| C1–C2–C3–C4 | −177.26(14) |
| C7–C2–C3–C4 | −54.57(18) |
| C2–C3–C4–C5 | 53.03(19) |
| C3–C4–C5–C8 | −177.49(14) |
| C3–C4–C5–C6 | −52.77(18) |
| C8–C5–C6–C7 | 175.80(14) |
| C4–C5–C6–C7 | 55.23(17) |
| C5–C6–C7–C2 | −58.79(18) |
| C1–C2–C7–C6 | −178.75(13) |
| C3–C2–C7–C6 | 58.03(17) |
| C6–C5–C8–N1 | 58.47(18) |
| C4–C5–C8–N1 | −179.09(14) |
| O4–C9–C10–O5 | 19.7(2) |
| O3–C9–C10–O5 | −160.57(17) |
| O4–C9–C10–C10^#1^ | −96.4(2) |
| O3–C9–C10–C10^#1^ | 83.3(2) |

Bonds and angles to hydrogen atoms were omitted.

Symmetry transformations used to generate equivalent atoms:

#1: -X, 1-Y, 1-Z;

## Table 1 Hydrogen bonds for TXA_MA_0m

| **D–H⋯A [pm]** | **d(D–H) [pm]** | **d(H⋯A) [pm]** | **d(D⋯A) [pm]** | **<(DHA) [°]** |
| --- | --- | --- | --- | --- |
| O2–H2O⋯O3 | 90(3) | 172(3) | 260.94(17) | 174(3) |
| N1–H1A⋯O4^#1^ | 91 | 191 | 282.11(19) | 173.7 |
| N1–H1B⋯O3^#2^ | 91 | 199 | 283.5(2) | 153.0 |
| N1–H1C⋯O1^#2^ | 91 | 234 | 302.05(17) | 131.0 |
| N1–H1C⋯O4^#3^ | 91 | 217 | 290.94(18) | 137.3 |
| N1–H1C⋯O5^a^#3^ | 91 | 241 | 307.4(3) | 130.2 |
| C8–H8B⋯O5^a^#4^ | 99 | 245 | 316.3(3) | 128.5 |
| O5^a–H5O^a⋯O1^#5^ | 89(5) | 189(5) | 274.3(3) | 161(4) |

Symmetry transformations used to generate equivalent atoms:

#1: 1-X, -Y, 1-Z; #2: 1+X, +Y, +Z; #3: 1+X, 0.5-Y, -0.5+Z; #4: 1-X, 1-Y, 1-Z; #5: +X, 0.5-Y, 0.5+Z;

## Bibliography

[1]	Bruker, *SAINT*, *V8.40B*, Bruker AXS Inc., Madison, Wisconsin, USA.

[2]	L. Krause, R. Herbst-Irmer, G. M. Sheldrick, D. Stalke, *J. Appl. Cryst.* **2015**, *48*, 3–10, doi:10.1107/S1600576714022985.

[3]	G. M. Sheldrick, *Acta Cryst.* **2015**, *A71*, 3–8, doi:10.1107/S2053273314026370.

[4]	G. M. Sheldrick, *Acta Cryst.* **2015**, *C71*, 3–8, doi:10.1107/S2053229614024218.

[5]	C. R. Groom, I. J. Bruno, M. P. Lightfoot, S. C. Ward, *Acta Cryst.* **2016**, *B72*, 171–179, doi:10.1107/S2052520616003954.

[6]	D. Kratzert, *FinalCif*, *V144*, https://dkratzert.de/finalcif.html.
