# Supplementary material for: Antifibrinolytic Agent: Synthesis and Characterization of Cocrystal and Salts of Tranexamic Acid
Source: ACS Omega. 2026 Oct 22;11(43):66176–200. doi: 10.1021/acsomega.6c03598 (PMC13737987; doi:10.1021/acsomega.6c03598)
Supplement: Supplementary file 3 [file ao6c03598_si_002.zip › report_TXA_Maleic_0m_complete-finalcif.docx]

# Structure Tables

A colourless, plate-shaped crystal was mounted on the goniometer. Data for TXA_Maleic_0m were collected from a shock-cooled single crystal at 150(2) K on a Bruker AXS D8 Quest four circle diffractometer with an I-mu-S 3.0 microsource X-ray tube using a HELIOS multilayer Montel optics as monochromator and a PhotonIII_C14 charge-integrating and photon counting pixel array detector. The diffractometer used CuKα radiation (λ = 154.178 pm). All data were integrated with SAINT V8.40B and a multi-scan absorption correction using SADABS 2016/2 was applied.^[1,2]^ The structure was solved by dual methods with SHELXT and refined by full-matrix least-squares methods against *F*^2^ using SHELXL-2019/2.^[3,4]^ All non-hydrogen atoms were refined with anisotropic displacement parameters. All C-bound hydrogen atoms were refined with isotropic displacement parameters. Some of their coordinates were refined freely and some on calculated positions using a riding model with their *U*_iso_ values constrained to 1.5 times the *U*_eq_ of their pivot atoms for terminal sp^3^ carbon atoms and 1.2 times for all other carbon atoms. Crystallographic data for the structures reported in this paper have been deposited with the Cambridge Crystallographic Data Centre.^[5]^ CCDC ?????? contain the supplementary crystallographic data for this paper. These data can be obtained free of charge from The Cambridge Crystallographic Data Centre via www.ccdc.cam.ac.uk/‌structures. This report and the CIF file were generated using FinalCif.^[6]^

## Table 1 Crystal data and structure refinement for TXA_Maleic_0m

| CCDC number |  |
| --- | --- |
| Empirical formula | C12H21NO7 |
| Formula weight | 291.30 |
| Temperature [K] | 150(2) |
| Crystal system | monoclinic |
| Space group (number) | $P2_{1}/c$ (14) |
| *a* [pm] | 2022.0(2) |
| *b* [pm] | 690.01(6) |
| *c* [pm] | 1044.94(7) |
| α [°] | 90 |
| β [°] | 93.759(9) |
| γ [°] | 90 |
| Volume [nm^3^] | 1.45(2) |
| *Z* | 4 |
| *ρ*_calc_ [gcm^−3^] | 1.330 |
| *μ* [mm^−1^] | 0.933 |
| *F*(000) | 624 |
| Crystal size [mm^3^] | 0.110×0.220×0.270 |
| Crystal colour | colourless |
| Crystal shape | plate |
| Radiation | CuKα (λ=154.178 pm) |
| 2θ range [°] | 4.38 to 160.16 (78 pm) |
| Index ranges | −25 ≤ h ≤ 23 −8 ≤ k ≤ 8 −12 ≤ l ≤ 13 |
| Reflections collected | 20854 |
| Independent reflections | 3132  *R*_int_ = 0.0638 *R*_sigma_ = 0.0513 |
| Completeness to  θ = 67.679° | 99.8 % |
| Data / Restraints / Parameters | 3132 / 0 / 195 |
| Absorption correction T_min_/T_max_ (method) | 0.6662 / 0.7543  (multi-scan) |
| Goodness-of-fit on *F*^2^ | 1.041 |
| Final *R* indexes  [*I*≥2σ(*I*)] | *R*_1_ = 0.0339 w*R*_2_ = 0.0868 |
| Final *R* indexes  [all data] | *R*_1_ = 0.0593 w*R*_2_ = 0.0904 |
| Largest peak/hole [eÅ^−3^] | 0.23/−0.18 |

## Table 1 Atomic coordinates and *U_eq_* [pm^2^] for TXA_Maleic_0m

| **Atom** | ***x*** | ***y*** | ***z*** | ***U*_eq_** |
| --- | --- | --- | --- | --- |
| O1 | 0.00436(4) | 0.38004(15) | 0.84769(9) | 442(3) |
| H1 | −0.0184(10) | 0.393(3) | 0.916(2) | 660.0 |
| O2 | 0.06371(4) | 0.61445(15) | 0.94414(9) | 399(2) |
| O3 | 0.65928(4) | 0.89860(14) | 0.31470(8) | 377(2) |
| O4 | 0.58334(4) | 0.88460(14) | 0.15263(7) | 350(2) |
| H4 | 0.5251(11) | 0.875(4) | 0.137(2) | 830.0(7) |
| O5 | 0.38044(4) | 0.92198(14) | 0.24368(8) | 355(2) |
| O6 | 0.46376(4) | 0.87953(13) | 0.12110(7) | 316.3(19) |
| O7 | 0.30151(4) | 0.94090(14) | 0.45110(8) | 357(2) |
| H7C | 0.3293(9) | 0.962(3) | 0.393(2) | 540.0 |
| H7D | 0.3180(8) | 0.998(3) | 0.526(2) | 540.0 |
| N1 | 0.31615(4) | 0.54641(16) | 0.49476(9) | 298(2) |
| H1A | 0.345026 | 0.501452 | 0.438476 | 450.0 |
| H1B | 0.334160 | 0.531908 | 0.576307 | 450.0 |
| H1C | 0.307782 | 0.674104 | 0.479081 | 450.0 |
| C1 | 0.05295(4) | 0.50608(17) | 0.85330(10) | 275(2) |
| C2 | 0.09327(4) | 0.50565(16) | 0.73652(10) | 256(2) |
| H2 | 0.063704 | 0.550520 | 0.661912 | 310.0 |
| C3 | 0.11688(5) | 0.30086(17) | 0.70608(11) | 323(2) |
| H3A | 0.078139 | 0.213806 | 0.692484 | 390.0 |
| H3B | 0.145235 | 0.250547 | 0.779497 | 390.0 |
| C4 | 0.15613(5) | 0.30279(18) | 0.58559(11) | 328(2) |
| H4A | 0.172171 | 0.170255 | 0.568715 | 390.0 |
| H4B | 0.126660 | 0.343466 | 0.511024 | 390.0 |
| C5 | 0.21517(4) | 0.44082(16) | 0.60091(9) | 249(2) |
| H5 | 0.245426 | 0.393805 | 0.673907 | 300.0 |
| C6 | 0.19218(5) | 0.64507(16) | 0.63282(10) | 280(2) |
| H6A | 0.164808 | 0.697625 | 0.558785 | 340.0 |
| H6B | 0.231345 | 0.730116 | 0.647941 | 340.0 |
| C7 | 0.15165(5) | 0.64686(16) | 0.75191(11) | 287(2) |
| H7A | 0.180560 | 0.610258 | 0.828220 | 340.0 |
| H7B | 0.134783 | 0.779423 | 0.765679 | 340.0 |
| C8 | 0.25292(5) | 0.43445(17) | 0.47902(10) | 280(2) |
| H8A | 0.262864 | 0.298038 | 0.458063 | 340.0 |
| H8B | 0.224795 | 0.489191 | 0.406751 | 340.0 |
| C9 | 0.60117(5) | 0.88064(16) | 0.27329(10) | 269(2) |
| C10 | 0.54929(5) | 0.85237(17) | 0.36747(10) | 289(2) |
| H10 | 0.565993 | 0.828306 | 0.453087 | 350.0 |
| C11 | 0.48338(5) | 0.85561(17) | 0.35024(10) | 288(2) |
| H11 | 0.460625 | 0.833453 | 0.425590 | 350.0 |
| C12 | 0.43973(5) | 0.88836(14) | 0.23196(10) | 240(2) |

*U*_eq_ is defined as 1/3 of the trace of the orthogonalized *U_ij_* tensor.

## Table 1 Anisotropic displacement parameters [pm^2^] for TXA_Maleic_0m. The anisotropic displacement factor exponent takes the form: −2π^2^[ *h*^2^*(a*)*^2^*U*_11_ *+ k*^2^*(b*)*^2^*U*_22_ + … + *2hka*b*U*_12_ ]

| **Atom** | ***U*_11_** | ***U*_22_** | ***U*_33_** | ***U*_23_** | ***U*_13_** | ***U*_12_** |
| --- | --- | --- | --- | --- | --- | --- |
| O1 | 354(4) | 546(6) | 449(5) | −182(4) | 205(4) | −196(4) |
| O2 | 310(4) | 540(5) | 361(4) | −151(4) | 133(3) | −143(4) |
| O3 | 279(4) | 540(5) | 310(4) | −53(4) | 13(3) | 9(3) |
| O4 | 287(4) | 565(5) | 202(4) | 15(3) | 52(3) | 4(3) |
| O5 | 275(4) | 516(5) | 278(4) | −14(4) | 48(3) | 26(3) |
| O6 | 289(4) | 471(5) | 189(3) | −27(3) | 23(3) | 9(3) |
| O7 | 294(4) | 487(5) | 294(4) | −38(4) | 48(3) | −53(3) |
| N1 | 222(4) | 437(5) | 243(4) | −22(4) | 69(3) | 13(4) |
| C1 | 185(4) | 338(5) | 304(5) | −27(4) | 36(4) | −5(4) |
| C2 | 198(4) | 316(5) | 257(5) | −11(4) | 32(3) | 2(4) |
| C3 | 324(5) | 305(5) | 354(6) | −24(4) | 128(4) | −38(4) |
| C4 | 329(5) | 332(6) | 334(5) | −82(4) | 110(4) | −46(4) |
| C5 | 214(4) | 313(5) | 223(5) | 5(4) | 38(3) | 28(4) |
| C6 | 248(4) | 296(5) | 306(5) | −6(4) | 87(4) | −10(4) |
| C7 | 238(4) | 313(5) | 321(5) | −64(4) | 86(4) | −23(4) |
| C8 | 238(4) | 360(6) | 246(5) | −30(4) | 44(4) | 10(4) |
| C9 | 286(5) | 291(5) | 230(5) | −6(4) | 27(4) | 22(4) |
| C10 | 326(5) | 356(5) | 184(4) | 42(4) | 18(4) | 0(4) |
| C11 | 323(5) | 358(5) | 188(4) | 32(4) | 59(4) | −20(4) |
| C12 | 273(4) | 238(4) | 212(5) | −6(3) | 30(4) | −20(4) |

## Table 1 Bond lengths and angles for TXA_Maleic_0m

| **Atom–Atom** | **Length [pm]** |
| --- | --- |
| O1–C1 | 131.06(14) |
| O2–C1 | 121.67(14) |
| O3–C9 | 123.14(14) |
| O4–C9 | 128.87(13) |
| O5–C12 | 123.49(13) |
| O6–C12 | 128.67(12) |
| N1–C8 | 149.35(13) |
| C1–C2 | 151.13(13) |
| C2–C7 | 153.10(14) |
| C2–C3 | 153.16(15) |
| C3–C4 | 153.17(13) |
| C4–C5 | 152.73(14) |
| C5–C6 | 152.77(14) |
| C5–C8 | 152.79(12) |
| C6–C7 | 153.43(13) |
| C9–C10 | 149.74(13) |
| C10–C11 | 133.32(16) |
| C11–C12 | 148.82(15) |
|  |  |
| **Atom–Atom–Atom** | **Angle [°]** |
| O2–C1–O1 | 122.54(9) |
| O2–C1–C2 | 123.56(9) |
| O1–C1–C2 | 113.89(9) |
| C1–C2–C7 | 111.52(9) |
| C1–C2–C3 | 111.19(9) |
| C7–C2–C3 | 111.12(8) |
| C2–C3–C4 | 110.37(9) |
| C5–C4–C3 | 111.27(9) |
| C4–C5–C6 | 110.56(8) |
| C4–C5–C8 | 108.93(8) |
| C6–C5–C8 | 112.60(8) |
| C5–C6–C7 | 111.77(8) |
| C2–C7–C6 | 111.02(9) |
| N1–C8–C5 | 111.35(8) |
| O3–C9–O4 | 122.74(9) |
| O3–C9–C10 | 118.40(10) |
| O4–C9–C10 | 118.86(9) |
| C11–C10–C9 | 130.26(10) |
| C10–C11–C12 | 130.38(9) |
| O5–C12–O6 | 121.63(10) |
| O5–C12–C11 | 118.23(9) |
| O6–C12–C11 | 120.13(9) |

Bonds and angles to hydrogen atoms were omitted.

## Table 1 Torsion angles for TXA_Maleic_0m

| **Atom–Atom–Atom–Atom** | **Torsion Angle [°]** |
| --- | --- |
| O2–C1–C2–C7 | 4.18(15) |
| O1–C1–C2–C7 | −176.62(10) |
| O2–C1–C2–C3 | 128.82(12) |
| O1–C1–C2–C3 | −51.98(13) |
| C1–C2–C3–C4 | 178.59(9) |
| C7–C2–C3–C4 | −56.55(12) |
| C2–C3–C4–C5 | 57.36(12) |
| C3–C4–C5–C6 | −56.61(12) |
| C3–C4–C5–C8 | 179.16(9) |
| C4–C5–C6–C7 | 55.26(12) |
| C8–C5–C6–C7 | 177.37(8) |
| C1–C2–C7–C6 | 179.99(9) |
| C3–C2–C7–C6 | 55.31(12) |
| C5–C6–C7–C2 | −54.82(12) |
| C4–C5–C8–N1 | −172.52(9) |
| C6–C5–C8–N1 | 64.47(11) |
| O3–C9–C10–C11 | 169.79(13) |
| O4–C9–C10–C11 | −10.42(19) |
| C9–C10–C11–C12 | 0.0(2) |
| C10–C11–C12–O5 | −166.12(12) |
| C10–C11–C12–O6 | 14.54(19) |

Bonds and angles to hydrogen atoms were omitted.

## Table 1 Hydrogen bonds for TXA_Maleic_0m

| **D–H⋯A [pm]** | **d(D–H) [pm]** | **d(H⋯A) [pm]** | **d(D⋯A) [pm]** | **<(DHA) [°]** |
| --- | --- | --- | --- | --- |
| O1–H1⋯O2#1 | 88(2) | 177(2) | 264.95(11) | 173(2) |
| O4–H4⋯O6 | 118(2) | 124(2) | 241.90(11) | 175(2) |
| O7–H7C⋯O5 | 87.0(19) | 194.4(19) | 277.77(11) | 160.0(19) |
| O7–H7D⋯O3#2 | 92(2) | 184(2) | 275.50(13) | 172.0(16) |
| N1–H1A⋯O4#3 | 91 | 196 | 285.64(11) | 168.8 |
| N1–H1B⋯O5#4 | 91 | 195 | 283.89(13) | 163.5 |
| N1–H1C⋯O7 | 91 | 187 | 277.26(15) | 173.1 |
| C2–H2⋯O1#5 | 100 | 266 | 333.43(14) | 125.2 |
| C8–H8A⋯O7#6 | 99 | 259 | 356.17(15) | 167.9 |
| C10–H10⋯O4#4 | 95 | 256 | 342.90(13) | 152.9 |
| C11–H11⋯O6#4 | 95 | 251 | 330.88(13) | 141.2 |

Symmetry transformations used to generate equivalent atoms:

#1: -X, 1-Y, 2-Z; #2: 1-X, 2-Y, 1-Z; #3: 1-X, -0.5+Y, 0.5-Z; #4: +X, 1.5-Y, 0.5+Z; #5: -X, 0.5+Y, 1.5-Z; #6: +X, -1+Y, +Z;

## Bibliography

[1]	Bruker, SAINT, V8.40B, Bruker AXS Inc., Madison, Wisconsin, USA.

[2]	L. Krause, R. Herbst-Irmer, G. M. Sheldrick, D. Stalke, J. Appl. Cryst. 2015, 48, 3–10, doi:10.1107/S1600576714022985.

[3]	G. M. Sheldrick, Acta Cryst. 2015, A71, 3–8, doi:10.1107/S2053273314026370.

[4]	G. M. Sheldrick, Acta Cryst. 2015, C71, 3–8, doi:10.1107/S2053229614024218.

[5]	C. R. Groom, I. J. Bruno, M. P. Lightfoot, S. C. Ward, Acta Cryst. 2016, B72, 171–179, doi:10.1107/S2052520616003954.

[6]	D. Kratzert, FinalCif, V144, https://dkratzert.de/finalcif.html.
