# Supplementary material for: Antifibrinolytic Agent: Synthesis and Characterization of Cocrystal and Salts of Tranexamic Acid
Source: ACS Omega. 2026 Oct 22;11(43):66176–200. doi: 10.1021/acsomega.6c03598 (PMC13737987; doi:10.1021/acsomega.6c03598)
Supplement: Supplementary file 3 [file ao6c03598_si_002.zip › report_TXA_Malonic_0m_complete-finalcif.docx]

# Structure Tables

A colourless, block-shaped crystal was mounted on the goniometer. Data for TXA_Malonic_0m were collected from a shock-cooled single crystal at 150(2) K on a Bruker AXS D8 Quest four circle diffractometer with an I-mu-S 3.0 microsource X-ray tube using a HELIOS multilayer Montel optics as monochromator and a PhotonIII_C14 charge-integrating and photon counting pixel array detector. The diffractometer used Cu*K_α_* radiation (λ = 1.54178 Å). All data were integrated with SAINT V8.40B and a multi-scan absorption correction using SADABS 2016/2 was applied.^[1,2]^ The structure was solved by dual methods with SHELXT and refined by full-matrix least-squares methods against *F*^2^ using SHELXL-2019/2.^[3,4]^ All non-hydrogen atoms were refined with anisotropic displacement parameters. All hydrogen atoms were refined with isotropic displacement parameters. Some of their coordinates were refined freely and some on calculated positions using a riding model with their *U*_iso_ values constrained to 1.5 times the *U*_eq_ of their pivot atoms for terminal sp^3^ carbon atoms and 1.2 times for all other carbon atoms. Crystallographic data for the structures reported in this paper have been deposited with the Cambridge Crystallographic Data Centre.^[5]^ CCDC ?????? contain the supplementary crystallographic data for this paper. These data can be obtained free of charge from The Cambridge Crystallographic Data Centre via www.ccdc.cam.ac.uk/‌structures. This report and the CIF file were generated using FinalCif.^[6]^

## Table 1 Crystal data and structure refinement for TXA_Malonic_0m

| CCDC number |  |
| --- | --- |
| Empirical formula | C_11_H_19_NO_6_ |
| Formula weight | 261.27 |
| Temperature [K] | 150(2) |
| Crystal system | orthorhombic |
| Space group (number) | $Pbca$ (61) |
| *a* [Å] | 10.2415(2) |
| *b* [Å] | 9.5341(2) |
| *c* [Å] | 25.7552(6) |
| α [°] | 90 |
| β [°] | 90 |
| γ [°] | 90 |
| Volume [Å^3^] | 2514.83(9) |
| *Z* | 8 |
| *ρ*_calc_ [gcm^−3^] | 1.380 |
| *μ* [mm^−1^] | 0.954 |
| *F*(000) | 1120 |
| Crystal size [mm^3^] | 0.130×0.180×0.190 |
| Crystal colour | colourless |
| Crystal shape | block |
| Radiation | Cu*K_α_* (λ=1.54178 Å) |
| 2θ range [°] | 6.86 to 159.88 (0.78 Å) |
| Index ranges | −13 ≤ h ≤ 12 −11 ≤ k ≤ 12 −32 ≤ l ≤ 28 |
| Reflections collected | 52728 |
| Independent reflections | 2730  *R*_int_ = 0.0293 *R*_sigma_ = 0.0143 |
| Completeness to  θ = 67.679° | 100.0 % |
| Data / Restraints / Parameters | 2730 / 0 / 184 |
| Absorption correction T_min_/T_max_ (method) | 0.6866 / 0.7543  (multi-scan) |
| Goodness-of-fit on *F*^2^ | 1.048 |
| Final *R* indexes  [*I*≥2σ(*I*)] | *R*_1_ = 0.0301 w*R*_2_ = 0.0803 |
| Final *R* indexes  [all data] | *R*_1_ = 0.0307 w*R*_2_ = 0.0807 |
| Largest peak/hole [eÅ^−3^] | 0.31/−0.19 |
| Extinction coefficient | 0.00084(15) |

## Table 1 Atomic coordinates and *U_eq_* [Å^2^] for TXA_Malonic_0m

| **Atom** | ***x*** | ***y*** | ***z*** | ***U*_eq_** |
| --- | --- | --- | --- | --- |
| O1 | 0.56678(8) | 0.64734(8) | 0.57144(3) | 0.02925(19) |
| O2 | 0.70761(8) | 0.47108(8) | 0.58021(3) | 0.02658(18) |
| H2A | 0.6766(19) | 0.477(2) | 0.6157(7) | 0.061(5) |
| O3 | 0.49875(7) | 0.34831(7) | 0.73036(3) | 0.02139(17) |
| O4 | 0.60792(7) | 0.47880(7) | 0.67327(3) | 0.02231(17) |
| O5 | 0.62927(7) | 0.06781(7) | 0.74922(3) | 0.02279(17) |
| H5A | 0.5772(19) | −0.014(2) | 0.7566(7) | 0.063(5) |
| O6 | 0.56333(8) | 0.03654(8) | 0.66719(3) | 0.02755(19) |
| N1 | 0.96316(8) | 0.73708(9) | 0.33458(3) | 0.01804(18) |
| H1A | 0.9748(15) | 0.7715(15) | 0.3025(6) | 0.035(4) |
| H1B | 1.0091(16) | 0.6525(17) | 0.3364(6) | 0.038(4) |
| H1C | 0.9975(15) | 0.7975(17) | 0.3588(6) | 0.037(4) |
| C1 | 0.64604(9) | 0.56706(10) | 0.55285(4) | 0.01729(19) |
| C2 | 0.68190(9) | 0.56609(9) | 0.49580(3) | 0.01595(19) |
| H2 | 0.658848 | 0.471776 | 0.481495 | 0.019 |
| C3 | 0.60477(9) | 0.67586(10) | 0.46545(4) | 0.0180(2) |
| H3A | 0.621682 | 0.769815 | 0.480375 | 0.022 |
| H3B | 0.510238 | 0.656167 | 0.468829 | 0.022 |
| C4 | 0.64226(9) | 0.67597(11) | 0.40808(4) | 0.0186(2) |
| H4A | 0.593629 | 0.750979 | 0.389848 | 0.022 |
| H4B | 0.617700 | 0.585049 | 0.392293 | 0.022 |
| C5 | 0.78913(9) | 0.69983(10) | 0.40134(3) | 0.01608(19) |
| H5 | 0.810979 | 0.792857 | 0.417030 | 0.019 |
| C6 | 0.86607(9) | 0.58739(10) | 0.43077(3) | 0.0178(2) |
| H6A | 0.847157 | 0.494094 | 0.415666 | 0.021 |
| H6B | 0.960785 | 0.605508 | 0.427025 | 0.021 |
| C7 | 0.82958(9) | 0.58745(10) | 0.48842(3) | 0.0177(2) |
| H7A | 0.855924 | 0.677780 | 0.504216 | 0.021 |
| H7B | 0.877465 | 0.511448 | 0.506398 | 0.021 |
| C8 | 0.82158(9) | 0.70691(11) | 0.34373(4) | 0.0198(2) |
| H8A | 0.798657 | 0.616481 | 0.327182 | 0.024 |
| H8B | 0.768263 | 0.781198 | 0.327248 | 0.024 |
| C9 | 0.58868(9) | 0.36730(10) | 0.69732(3) | 0.01716(19) |
| C10 | 0.68200(10) | 0.24430(10) | 0.68789(4) | 0.0216(2) |
| H10A | 0.710050 | 0.244809 | 0.651106 | 0.026 |
| H10B | 0.760718 | 0.256367 | 0.709749 | 0.026 |
| C11 | 0.61930(9) | 0.10477(10) | 0.70022(4) | 0.0190(2) |

*U*_eq_ is defined as 1/3 of the trace of the orthogonalized *U_ij_* tensor.

## Table 1 Anisotropic displacement parameters [Å^2^] for TXA_Malonic_0m. The anisotropic displacement factor exponent takes the form: −2π^2^[ *h*^2^*(a*)*^2^*U*_11_ *+ k*^2^*(b*)*^2^*U*_22_ + … + *2hka*b*U*_12_ ]

| **Atom** | ***U*_11_** | ***U*_22_** | ***U*_33_** | ***U*_23_** | ***U*_13_** | ***U*_12_** |
| --- | --- | --- | --- | --- | --- | --- |
| O1 | 0.0388(4) | 0.0301(4) | 0.0188(3) | 0.0008(3) | 0.0082(3) | 0.0123(3) |
| O2 | 0.0314(4) | 0.0309(4) | 0.0174(3) | 0.0059(3) | 0.0052(3) | 0.0094(3) |
| O3 | 0.0274(4) | 0.0186(3) | 0.0182(3) | 0.0020(2) | 0.0074(3) | 0.0024(3) |
| O4 | 0.0298(4) | 0.0192(3) | 0.0179(3) | 0.0038(3) | 0.0040(3) | −0.0001(3) |
| O5 | 0.0313(4) | 0.0195(3) | 0.0176(3) | −0.0003(3) | 0.0010(3) | −0.0052(3) |
| O6 | 0.0373(4) | 0.0236(4) | 0.0218(4) | −0.0015(3) | −0.0032(3) | −0.0050(3) |
| N1 | 0.0209(4) | 0.0194(4) | 0.0138(4) | −0.0006(3) | 0.0024(3) | −0.0021(3) |
| C1 | 0.0182(4) | 0.0170(4) | 0.0166(4) | −0.0005(3) | 0.0018(3) | −0.0030(3) |
| C2 | 0.0174(4) | 0.0160(4) | 0.0145(4) | −0.0007(3) | 0.0020(3) | −0.0012(3) |
| C3 | 0.0159(4) | 0.0204(4) | 0.0178(4) | 0.0006(3) | 0.0021(3) | 0.0006(3) |
| C4 | 0.0178(4) | 0.0218(5) | 0.0162(4) | 0.0018(3) | −0.0007(3) | −0.0007(3) |
| C5 | 0.0179(4) | 0.0166(4) | 0.0138(4) | 0.0006(3) | 0.0009(3) | −0.0009(3) |
| C6 | 0.0175(4) | 0.0189(4) | 0.0172(4) | 0.0021(3) | 0.0032(3) | 0.0012(3) |
| C7 | 0.0163(4) | 0.0211(4) | 0.0157(4) | 0.0025(3) | 0.0009(3) | 0.0004(4) |
| C8 | 0.0196(5) | 0.0248(5) | 0.0151(4) | 0.0011(3) | 0.0003(3) | −0.0038(4) |
| C9 | 0.0217(4) | 0.0179(4) | 0.0119(4) | −0.0013(3) | −0.0008(3) | −0.0018(4) |
| C10 | 0.0232(5) | 0.0192(5) | 0.0225(5) | 0.0009(4) | 0.0069(4) | −0.0003(4) |
| C11 | 0.0203(4) | 0.0178(4) | 0.0188(4) | −0.0016(3) | 0.0040(3) | 0.0023(4) |

## Table 1 Bond lengths and angles for TXA_Malonic_0m

| **Atom–Atom** | **Length [Å]** |
| --- | --- |
| O1–C1 | 1.2141(12) |
| O2–C1 | 1.3159(12) |
| O3–C9 | 1.2670(12) |
| O4–C9 | 1.2460(12) |
| O5–C11 | 1.3142(12) |
| O6–C11 | 1.2148(13) |
| N1–C8 | 1.4969(12) |
| C1–C2 | 1.5145(12) |
| C2–C3 | 1.5265(13) |
| C2–C7 | 1.5379(12) |
| C3–C4 | 1.5266(12) |
| C4–C5 | 1.5312(13) |
| C5–C8 | 1.5220(12) |
| C5–C6 | 1.5313(12) |
| C6–C7 | 1.5309(12) |
| C9–C10 | 1.5322(13) |
| C10–C11 | 1.5109(13) |
|  |  |
| **Atom–Atom–Atom** | **Angle [°]** |
| O1–C1–O2 | 123.19(9) |
| O1–C1–C2 | 123.28(9) |
| O2–C1–C2 | 113.52(8) |
| C1–C2–C3 | 111.53(7) |
| C1–C2–C7 | 110.95(7) |
| C3–C2–C7 | 110.78(7) |
| C2–C3–C4 | 111.46(8) |
| C3–C4–C5 | 110.91(7) |
| C8–C5–C4 | 109.37(7) |
| C8–C5–C6 | 113.66(8) |
| C4–C5–C6 | 110.20(7) |
| C7–C6–C5 | 110.74(7) |
| C6–C7–C2 | 111.10(8) |
| N1–C8–C5 | 111.93(8) |
| O4–C9–O3 | 124.80(9) |
| O4–C9–C10 | 118.40(8) |
| O3–C9–C10 | 116.78(8) |
| C11–C10–C9 | 112.05(8) |
| O6–C11–O5 | 124.44(9) |
| O6–C11–C10 | 121.65(9) |
| O5–C11–C10 | 113.89(8) |

Bonds and angles to hydrogen atoms were omitted.

## Table 1 Torsion angles for TXA_Malonic_0m

| **Atom–Atom–Atom–Atom** | **Torsion Angle [°]** |
| --- | --- |
| O1–C1–C2–C3 | −0.90(13) |
| O2–C1–C2–C3 | 178.12(8) |
| O1–C1–C2–C7 | 123.12(10) |
| O2–C1–C2–C7 | −57.85(11) |
| C1–C2–C3–C4 | 179.29(7) |
| C7–C2–C3–C4 | 55.17(10) |
| C2–C3–C4–C5 | −56.56(10) |
| C3–C4–C5–C8 | −177.05(8) |
| C3–C4–C5–C6 | 57.31(10) |
| C8–C5–C6–C7 | 179.37(8) |
| C4–C5–C6–C7 | −57.46(10) |
| C5–C6–C7–C2 | 56.65(10) |
| C1–C2–C7–C6 | −179.67(7) |
| C3–C2–C7–C6 | −55.21(10) |
| C4–C5–C8–N1 | 177.24(8) |
| C6–C5–C8–N1 | −59.14(11) |
| O4–C9–C10–C11 | −155.92(9) |
| O3–C9–C10–C11 | 25.78(12) |
| C9–C10–C11–O6 | 92.14(11) |
| C9–C10–C11–O5 | −86.58(10) |

Bonds and angles to hydrogen atoms were omitted.

## Table 1 Hydrogen bonds for TXA_Malonic_0m

| **D–H⋯A [Å]** | **d(D–H) [Å]** | **d(H⋯A) [Å]** | **d(D⋯A) [Å]** | **<(DHA) [°]** |
| --- | --- | --- | --- | --- |
| O2–H2A⋯O4 | 0.968(19) | 1.642(19) | 2.6063(10) | 173.3(18) |
| O5–H5A⋯O3^#1^ | 0.97(2) | 1.56(2) | 2.5249(10) | 176.4(19) |
| N1–H1A⋯O3^#2^ | 0.897(16) | 2.198(15) | 2.8319(11) | 127.2(12) |
| N1–H1A⋯O5^#2^ | 0.897(16) | 2.316(16) | 3.0314(11) | 136.6(13) |
| N1–H1B⋯O6^#3^ | 0.935(16) | 1.888(17) | 2.8035(11) | 165.7(14) |
| N1–H1C⋯O1^#4^ | 0.919(16) | 2.002(16) | 2.8633(11) | 155.4(13) |
| N1–H1C⋯O4^#4^ | 0.919(16) | 2.551(15) | 3.0946(11) | 118.3(11) |

Symmetry transformations used to generate equivalent atoms:

#1: 1-X, -0.5+Y, 1.5-Z; #2: 1.5-X, 1-Y, -0.5+Z; #3: 0.5+X, 0.5-Y, 1-Z; #4: 0.5+X, 1.5-Y, 1-Z;

## Bibliography

[1]	Bruker, *SAINT*, *V8.40B*, Bruker AXS Inc., Madison, Wisconsin, USA.

[2]	L. Krause, R. Herbst-Irmer, G. M. Sheldrick, D. Stalke, *J. Appl. Cryst.* **2015**, *48*, 3–10, doi:10.1107/S1600576714022985.

[3]	G. M. Sheldrick, *Acta Cryst.* **2015**, *A71*, 3–8, doi:10.1107/S2053273314026370.

[4]	G. M. Sheldrick, *Acta Cryst.* **2015**, *C71*, 3–8, doi:10.1107/S2053229614024218.

[5]	C. R. Groom, I. J. Bruno, M. P. Lightfoot, S. C. Ward, *Acta Cryst.* **2016**, *B72*, 171–179, doi:10.1107/S2052520616003954.

[6]	D. Kratzert, *FinalCif*, *V144*, https://dkratzert.de/finalcif.html.
