# Supplementary material for: Antifibrinolytic Agent: Synthesis and Characterization of Cocrystal and Salts of Tranexamic Acid
Source: ACS Omega. 2026 Oct 22;11(43):66176–200. doi: 10.1021/acsomega.6c03598 (PMC13737987; doi:10.1021/acsomega.6c03598)
Supplement: Supplementary file 3 [file ao6c03598_si_002.zip › report_TXA_Para_0m_complete-finalcif.docx]

# Structure Tables

A colourless, plate-shaped crystal was mounted on the goniometer. Data for TXA_Para_0m were collected from a shock-cooled single crystal at 150(2) K on a Bruker AXS D8 Quest four circle diffractometer with an I-mu-S 3.0 microsource X-ray tube using a HELIOS multilayer Montel optics as monochromator and a PhotonIII_C14 charge-integrating and photon counting pixel array detector. The diffractometer used Cu*K_α_* radiation (λ = 1.54178 Å). All data were integrated with SAINT V8.40B and a multi-scan absorption correction using SADABS 2016/2 was applied.^[1,2]^ The structure was solved by dual methods with SHELXT and refined by full-matrix least-squares methods against *F*^2^ using SHELXL-2019/2.^[3,4]^ All non-hydrogen atoms were refined with anisotropic displacement parameters. All C-bound hydrogen atoms were refined with isotropic displacement parameters. Some of their coordinates were refined freely and some on calculated positions using a riding model with their *U*_iso_ values constrained to 1.5 times the *U*_eq_ of their pivot atoms for terminal sp^3^ carbon atoms and 1.2 times for all other carbon atoms. Crystallographic data for the structures reported in this paper have been deposited with the Cambridge Crystallographic Data Centre.^[5]^ CCDC ?????? contain the supplementary crystallographic data for this paper. These data can be obtained free of charge from The Cambridge Crystallographic Data Centre via www.ccdc.cam.ac.uk/‌structures. This report and the CIF file were generated using FinalCif.^[6]^

## Table 1 Crystal data and structure refinement for TXA_Para_0m

| CCDC number |  |
| --- | --- |
| Empirical formula | C_32_H_60_N_4_O_11_ |
| Formula weight | 676.84 |
| Temperature [K] | 150(2) |
| Crystal system | monoclinic |
| Space group (number) | $P2_{1}$ (4) |
| *a* [Å] | 6.3475(2) |
| *b* [Å] | 23.4719(7) |
| *c* [Å] | 23.6876(7) |
| α [°] | 90 |
| β [°] | 90.3581(15) |
| γ [°] | 90 |
| Volume [Å^3^] | 3529.10(19) |
| *Z* | 4 |
| *ρ*_calc_ [gcm^−3^] | 1.274 |
| *μ* [mm^−1^] | 0.788 |
| *F*(000) | 1472 |
| Crystal size [mm^3^] | 0.090×0.200×0.210 |
| Crystal colour | colourless |
| Crystal shape | plate |
| Radiation | Cu*K_α_* (λ=1.54178 Å) |
| 2θ range [°] | 5.30 to 160.24 (0.78 Å) |
| Index ranges | −8 ≤ h ≤ 7 −25 ≤ k ≤ 29 −29 ≤ l ≤ 30 |
| Reflections collected | 45797 |
| Independent reflections | 14244  *R*_int_ = 0.0551 *R*_sigma_ = 0.0560 |
| Completeness to  θ = 67.679° | 99.9 % |
| Data / Restraints / Parameters | 14244 / 19 / 894 |
| Absorption correction T_min_/T_max_ (method) | 0.6093 / 0.7543  (multi-scan) |
| Goodness-of-fit on *F*^2^ | 1.051 |
| Final *R* indexes  [*I*≥2σ(*I*)] | *R*_1_ = 0.0473 w*R*_2_ = 0.1259 |
| Final *R* indexes  [all data] | *R*_1_ = 0.0485 w*R*_2_ = 0.1268 |
| Largest peak/hole [eÅ^−3^] | 0.38/−0.23 |
| Flack X parameter | 0.07(6) |

## Refinement details for TXA_Para_0m

The structure is metrically close to orthorhombic and is twinned by this symmetry. Application of the twin matrix 1 0 0 0 -1 0 -0.048 0 -1 yielded a BASF value of 0.1772(17). Water H atom positions were refined and O-H and H...H distances were restrained to 0.84(2) and 1.36(2) Angstrom, respectively.

## Table 1 Atomic coordinates and *U_eq_* [Å^2^] for TXA_Para_0m

| **Atom** | ***x*** | ***y*** | ***z*** | ***U*_eq_** |
| --- | --- | --- | --- | --- |
| O1_1 | 0.4527(4) | 0.77487(11) | 0.20139(11) | 0.0225(5) |
| O2_1 | 0.7423(4) | 0.75512(12) | 0.15305(12) | 0.0292(6) |
| N1_1 | 0.0837(5) | 0.49164(12) | 0.19419(12) | 0.0177(6) |
| H1A_1 | −0.017730 | 0.464563 | 0.189775 | 0.027 |
| H1B_1 | 0.068719 | 0.508767 | 0.228396 | 0.027 |
| H1C_1 | 0.213012 | 0.475068 | 0.192229 | 0.027 |
| C1_1 | 0.5564(5) | 0.74432(15) | 0.16765(14) | 0.0164(6) |
| C2_1 | 0.4540(5) | 0.68970(14) | 0.14527(14) | 0.0159(6) |
| H2_1 | 0.514239 | 0.681087 | 0.107311 | 0.019 |
| C3_1 | 0.2154(5) | 0.69449(15) | 0.13928(14) | 0.0177(6) |
| H3A_1 | 0.181223 | 0.723825 | 0.110628 | 0.021 |
| H3B_1 | 0.155372 | 0.707008 | 0.175707 | 0.021 |
| C4_1 | 0.1139(6) | 0.63781(15) | 0.12185(14) | 0.0195(7) |
| H4A_1 | −0.041244 | 0.642145 | 0.120716 | 0.023 |
| H4B_1 | 0.161511 | 0.627218 | 0.083539 | 0.023 |
| C5_1 | 0.1735(6) | 0.59069(14) | 0.16350(14) | 0.0167(6) |
| H5_1 | 0.122703 | 0.602619 | 0.201584 | 0.020 |
| C6_1 | 0.4138(6) | 0.58457(15) | 0.16725(16) | 0.0193(7) |
| H6A_1 | 0.450656 | 0.554353 | 0.194752 | 0.023 |
| H6B_1 | 0.469879 | 0.573341 | 0.129969 | 0.023 |
| C7_1 | 0.5134(5) | 0.64098(14) | 0.18577(15) | 0.0187(7) |
| H7A_1 | 0.668578 | 0.636795 | 0.186928 | 0.022 |
| H7B_1 | 0.465422 | 0.650528 | 0.224313 | 0.022 |
| C8_1 | 0.0622(6) | 0.53531(15) | 0.14835(15) | 0.0200(7) |
| H8A_1 | −0.089095 | 0.543115 | 0.141481 | 0.024 |
| H8B_1 | 0.122386 | 0.519950 | 0.113029 | 0.024 |
| O1_2 | 0.4872(4) | 0.44072(11) | 0.20472(11) | 0.0225(5) |
| O2_2 | 0.7891(4) | 0.41052(12) | 0.16821(13) | 0.0272(6) |
| N1_2 | 0.0688(5) | 0.15982(12) | 0.19808(12) | 0.0181(6) |
| H1A_2 | −0.033371 | 0.133374 | 0.191619 | 0.027 |
| H1B_2 | 0.044301 | 0.177561 | 0.231565 | 0.027 |
| H1C_2 | 0.196723 | 0.142358 | 0.199407 | 0.027 |
| C1_2 | 0.5950(6) | 0.40594(14) | 0.17691(15) | 0.0171(6) |
| C2_2 | 0.4883(6) | 0.35187(14) | 0.15357(14) | 0.0173(7) |
| H2_2 | 0.555225 | 0.342137 | 0.116703 | 0.021 |
| C3_2 | 0.2520(6) | 0.35949(15) | 0.14355(16) | 0.0219(7) |
| H3A_2 | 0.229411 | 0.389070 | 0.114341 | 0.026 |
| H3B_2 | 0.185640 | 0.372883 | 0.178857 | 0.026 |
| C4_2 | 0.1458(6) | 0.30416(16) | 0.12459(16) | 0.0237(8) |
| H4A_2 | 0.202647 | 0.292415 | 0.087546 | 0.028 |
| H4B_2 | −0.007539 | 0.310525 | 0.120128 | 0.028 |
| C5_2 | 0.1845(6) | 0.25690(14) | 0.16791(14) | 0.0181(7) |
| H5_2 | 0.127703 | 0.270180 | 0.204922 | 0.022 |
| C6_2 | 0.4209(6) | 0.24796(16) | 0.17515(18) | 0.0251(8) |
| H6A_2 | 0.481911 | 0.235549 | 0.138801 | 0.030 |
| H6B_2 | 0.446559 | 0.217511 | 0.203286 | 0.030 |
| C7_2 | 0.5294(6) | 0.30296(15) | 0.19469(17) | 0.0230(7) |
| H7A_2 | 0.476728 | 0.313552 | 0.232511 | 0.028 |
| H7B_2 | 0.683048 | 0.296335 | 0.197871 | 0.028 |
| C8_2 | 0.0667(6) | 0.20286(15) | 0.15147(15) | 0.0212(7) |
| H8A_2 | −0.080977 | 0.212599 | 0.141846 | 0.025 |
| H8B_2 | 0.132443 | 0.186122 | 0.117505 | 0.025 |
| O1_3 | 0.4615(4) | 0.10949(11) | 0.21112(11) | 0.0235(5) |
| O2_3 | 0.7722(5) | 0.07913(13) | 0.18026(14) | 0.0347(7) |
| N1_3 | 0.0548(5) | −0.17092(12) | 0.18736(12) | 0.0180(6) |
| H1A_3 | −0.043166 | −0.197870 | 0.178603 | 0.027 |
| H1B_3 | 0.024158 | −0.155287 | 0.221491 | 0.027 |
| H1C_3 | 0.184674 | −0.187280 | 0.188888 | 0.027 |
| C1_3 | 0.5772(6) | 0.07532(15) | 0.18368(15) | 0.0184(7) |
| C2_3 | 0.4728(6) | 0.02342(15) | 0.15619(14) | 0.0180(7) |
| H2_3 | 0.544241 | 0.016101 | 0.119400 | 0.022 |
| C3_3 | 0.2367(6) | 0.03138(15) | 0.14436(15) | 0.0207(7) |
| H3A_3 | 0.165888 | 0.043044 | 0.179641 | 0.025 |
| H3B_3 | 0.217504 | 0.062340 | 0.116418 | 0.025 |
| C4_3 | 0.1332(6) | −0.02279(15) | 0.12192(15) | 0.0210(7) |
| H4A_3 | 0.192893 | −0.032362 | 0.084587 | 0.025 |
| H4B_3 | −0.019933 | −0.016340 | 0.116991 | 0.025 |
| C5_3 | 0.1692(6) | −0.07235(15) | 0.16258(14) | 0.0172(7) |
| H5_3 | 0.109904 | −0.061053 | 0.199953 | 0.021 |
| C6_3 | 0.4073(6) | −0.08200(16) | 0.17089(18) | 0.0249(8) |
| H6A_3 | 0.430701 | −0.114107 | 0.197294 | 0.030 |
| H6B_3 | 0.472131 | −0.091985 | 0.134295 | 0.030 |
| C7_3 | 0.5110(6) | −0.02813(15) | 0.19451(17) | 0.0225(7) |
| H7A_3 | 0.664497 | −0.034563 | 0.198539 | 0.027 |
| H7B_3 | 0.453751 | −0.020094 | 0.232464 | 0.027 |
| C8_3 | 0.0526(6) | −0.12542(15) | 0.14312(14) | 0.0206(7) |
| H8A_3 | −0.095031 | −0.115336 | 0.133872 | 0.025 |
| H8B_3 | 0.119169 | −0.140194 | 0.108380 | 0.025 |
| N1_4 | 0.5635(5) | 0.15951(13) | 0.31309(12) | 0.0184(6) |
| H1A_4 | 0.537423 | 0.143405 | 0.278800 | 0.028 |
| H1B_4 | 0.691663 | 0.176847 | 0.312663 | 0.028 |
| H1C_4 | 0.462017 | 0.185761 | 0.320696 | 0.028 |
| O1_4 | 1.2613(4) | −0.10023(12) | 0.33627(13) | 0.0290(6) |
| O2_4 | 0.9638(4) | −0.11901(11) | 0.28939(11) | 0.0231(5) |
| C1_4 | 1.0720(6) | −0.09008(14) | 0.32386(15) | 0.0173(6) |
| C2_4 | 0.9722(5) | −0.03709(14) | 0.34967(14) | 0.0163(6) |
| H2_4 | 1.040271 | −0.029984 | 0.387194 | 0.020 |
| C3_4 | 1.0181(6) | 0.01437(15) | 0.31107(16) | 0.0204(7) |
| H3A_4 | 1.172314 | 0.020281 | 0.308963 | 0.025 |
| H3B_4 | 0.965641 | 0.006119 | 0.272484 | 0.025 |
| C4_4 | 0.9142(6) | 0.06856(15) | 0.33271(16) | 0.0208(7) |
| H4A_4 | 0.942021 | 0.100179 | 0.306117 | 0.025 |
| H4B_4 | 0.976142 | 0.078886 | 0.369785 | 0.025 |
| C5_4 | 0.6760(5) | 0.06049(14) | 0.33883(14) | 0.0170(7) |
| H5_4 | 0.618186 | 0.049428 | 0.301029 | 0.020 |
| C6_4 | 0.6339(6) | 0.01141(15) | 0.37950(15) | 0.0206(7) |
| H6A_4 | 0.691193 | 0.021029 | 0.417275 | 0.025 |
| H6B_4 | 0.480004 | 0.005710 | 0.383109 | 0.025 |
| C7_4 | 0.7352(6) | −0.04336(15) | 0.35857(15) | 0.0205(7) |
| H7A_4 | 0.667850 | −0.054709 | 0.322481 | 0.025 |
| H7B_4 | 0.709733 | −0.074071 | 0.386347 | 0.025 |
| C8_4 | 0.5622(6) | 0.11440(15) | 0.35757(15) | 0.0209(7) |
| H8A_4 | 0.414621 | 0.104844 | 0.366962 | 0.025 |
| H8B_4 | 0.630917 | 0.129353 | 0.392158 | 0.025 |
| O1_5 | 1.2489(5) | 0.23382(12) | 0.34784(13) | 0.0308(6) |
| O2_5 | 0.9596(4) | 0.21368(11) | 0.29823(11) | 0.0234(5) |
| N1_5 | 0.5875(5) | 0.49658(12) | 0.30647(12) | 0.0175(6) |
| H1A_5 | 0.563784 | 0.479173 | 0.272695 | 0.026 |
| H1B_5 | 0.718878 | 0.512103 | 0.306773 | 0.026 |
| H1C_5 | 0.490153 | 0.524527 | 0.311643 | 0.026 |
| C1_5 | 1.0645(6) | 0.24392(15) | 0.33224(15) | 0.0195(7) |
| C2_5 | 0.9635(5) | 0.29894(15) | 0.35402(14) | 0.0171(6) |
| H2_5 | 1.027196 | 0.308218 | 0.391633 | 0.020 |
| C3_5 | 1.0176(6) | 0.34738(15) | 0.31259(16) | 0.0196(7) |
| H3A_5 | 1.172486 | 0.351831 | 0.310686 | 0.024 |
| H3B_5 | 0.965610 | 0.337322 | 0.274380 | 0.024 |
| C4_5 | 0.9182(6) | 0.40401(15) | 0.33106(16) | 0.0221(7) |
| H4A_5 | 0.979539 | 0.415959 | 0.367718 | 0.027 |
| H4B_5 | 0.949844 | 0.433876 | 0.302854 | 0.027 |
| C5_5 | 0.6791(6) | 0.39758(14) | 0.33678(14) | 0.0168(6) |
| H5_5 | 0.621567 | 0.385243 | 0.299336 | 0.020 |
| C6_5 | 0.6277(6) | 0.35116(15) | 0.37977(15) | 0.0195(7) |
| H6A_5 | 0.473066 | 0.347063 | 0.383050 | 0.023 |
| H6B_5 | 0.684900 | 0.361917 | 0.417258 | 0.023 |
| C7_5 | 0.7246(6) | 0.29433(15) | 0.36087(15) | 0.0187(7) |
| H7A_5 | 0.692452 | 0.264580 | 0.389158 | 0.022 |
| H7B_5 | 0.660379 | 0.282651 | 0.324482 | 0.022 |
| C8_5 | 0.5706(6) | 0.45389(15) | 0.35282(15) | 0.0203(7) |
| H8A_5 | 0.420170 | 0.446572 | 0.360859 | 0.024 |
| H8B_5 | 0.636868 | 0.469387 | 0.387507 | 0.024 |
| O1_6 | 1.3004(4) | 0.58031(12) | 0.31970(13) | 0.0282(6) |
| O2_6 | 0.9900(4) | 0.54325(11) | 0.29557(11) | 0.0221(5) |
| N1_6 | 0.5694(5) | 0.82686(13) | 0.30224(13) | 0.0195(6) |
| H1A_6 | 0.551265 | 0.809603 | 0.268147 | 0.029 |
| H1B_6 | 0.693845 | 0.846093 | 0.302570 | 0.029 |
| H1C_6 | 0.461751 | 0.851702 | 0.308248 | 0.029 |
| C1_6 | 1.1040(6) | 0.58150(15) | 0.31792(14) | 0.0171(6) |
| C2_6 | 0.9984(5) | 0.63478(14) | 0.34176(14) | 0.0157(6) |
| H2_6 | 1.070269 | 0.644773 | 0.378116 | 0.019 |
| C3_6 | 1.0310(6) | 0.68398(15) | 0.30044(15) | 0.0205(7) |
| H3A_6 | 0.974071 | 0.673071 | 0.262972 | 0.025 |
| H3B_6 | 1.183874 | 0.691106 | 0.296279 | 0.025 |
| C4_6 | 0.9231(6) | 0.73887(16) | 0.32010(17) | 0.0222(7) |
| H4A_6 | 0.942201 | 0.768949 | 0.291278 | 0.027 |
| H4B_6 | 0.989636 | 0.752102 | 0.355665 | 0.027 |
| C5_6 | 0.6868(5) | 0.72903(15) | 0.32969(14) | 0.0174(7) |
| H5_6 | 0.623223 | 0.715905 | 0.293203 | 0.021 |
| C6_6 | 0.6579(6) | 0.68155(16) | 0.37308(15) | 0.0210(7) |
| H6A_6 | 0.719497 | 0.693573 | 0.409686 | 0.025 |
| H6B_6 | 0.505710 | 0.674528 | 0.378704 | 0.025 |
| C7_6 | 0.7640(6) | 0.62672(15) | 0.35359(14) | 0.0192(7) |
| H7A_6 | 0.692922 | 0.612968 | 0.318845 | 0.023 |
| H7B_6 | 0.746883 | 0.597181 | 0.383043 | 0.023 |
| C8_6 | 0.5714(6) | 0.78276(16) | 0.34785(15) | 0.0217(7) |
| H8A_6 | 0.424603 | 0.772941 | 0.357843 | 0.026 |
| H8B_6 | 0.640870 | 0.798645 | 0.381915 | 0.026 |
| O3_7 | 0.5112(5) | 0.45433(13) | 0.00910(14) | 0.0343(6) |
| H3A_7 | 0.632349 | 0.445696 | 0.020701 | 0.051 |
| O4_7 | 0.0841(5) | 0.70324(13) | −0.01404(13) | 0.0323(6) |
| N2_7 | 0.4389(5) | 0.69004(15) | −0.01228(14) | 0.0271(7) |
| H2_7 | 0.559405 | 0.708353 | −0.015008 | 0.032 |
| C9_7 | 0.4967(6) | 0.51176(17) | 0.00337(15) | 0.0251(8) |
| C10_7 | 0.3060(6) | 0.53519(18) | −0.01443(16) | 0.0284(8) |
| H10_7 | 0.189401 | 0.510810 | −0.021616 | 0.034 |
| C11_7 | 0.2831(6) | 0.59365(17) | −0.02189(17) | 0.0267(8) |
| H11_7 | 0.153420 | 0.608893 | −0.035254 | 0.032 |
| C12_7 | 0.4513(6) | 0.62964(17) | −0.00964(15) | 0.0238(7) |
| C13_7 | 0.6439(6) | 0.60654(17) | 0.00740(16) | 0.0247(7) |
| H13_7 | 0.759907 | 0.631063 | 0.014808 | 0.030 |
| C14_7 | 0.6681(6) | 0.54788(18) | 0.01368(17) | 0.0283(8) |
| H14_7 | 0.800237 | 0.532472 | 0.024910 | 0.034 |
| C15_7 | 0.2628(6) | 0.72267(17) | −0.01108(15) | 0.0249(8) |
| C16_7 | 0.3006(7) | 0.78579(18) | −0.00475(19) | 0.0339(9) |
| H16A_7 | 0.317584 | 0.795165 | 0.035331 | 0.051 |
| H16B_7 | 0.180232 | 0.806786 | −0.020371 | 0.051 |
| H16C_7 | 0.428680 | 0.796419 | −0.025118 | 0.051 |
| O3_8 | 0.9574(5) | 0.43195(12) | 0.48574(12) | 0.0307(6) |
| H3A_8 | 0.840413 | 0.422315 | 0.499110 | 0.046 |
| O4_8 | 1.4075(4) | 0.67730(13) | 0.49988(13) | 0.0338(6) |
| N2_8 | 1.0533(5) | 0.66831(15) | 0.49468(14) | 0.0285(7) |
| H2_8 | 0.934671 | 0.687673 | 0.495695 | 0.034 |
| C9_8 | 0.9757(6) | 0.49020(17) | 0.48724(15) | 0.0246(7) |
| C10_8 | 1.1643(6) | 0.51384(18) | 0.46970(16) | 0.0269(8) |
| H10_8 | 1.273229 | 0.489934 | 0.455899 | 0.032 |
| C11_8 | 1.1946(6) | 0.57231(18) | 0.47223(15) | 0.0263(8) |
| H11_8 | 1.323933 | 0.588353 | 0.460038 | 0.032 |
| C12_8 | 1.0349(6) | 0.60777(17) | 0.49270(15) | 0.0241(7) |
| C13_8 | 0.8454(6) | 0.58384(19) | 0.50915(16) | 0.0281(8) |
| H13_8 | 0.734896 | 0.607681 | 0.522157 | 0.034 |
| C14_8 | 0.8156(6) | 0.52524(18) | 0.50676(16) | 0.0274(8) |
| H14_8 | 0.685747 | 0.509142 | 0.518482 | 0.033 |
| C15_8 | 1.2320(6) | 0.69898(17) | 0.49515(15) | 0.0243(7) |
| C16_8 | 1.2028(7) | 0.76212(18) | 0.48818(18) | 0.0318(9) |
| H16A_8 | 1.066531 | 0.773399 | 0.503890 | 0.048 |
| H16B_8 | 1.206626 | 0.771865 | 0.447976 | 0.048 |
| H16C_8 | 1.316106 | 0.782265 | 0.508138 | 0.048 |
| O1_9 | 0.2224(5) | 0.26237(13) | −0.03707(13) | 0.0324(6) |
| H1A_9 | 0.127(8) | 0.239(2) | −0.028(2) | 0.049 |
| H1B_9 | 0.224(9) | 0.261(2) | −0.0730(11) | 0.049 |
| O2_9 | 0.1735(5) | 0.37950(14) | −0.01109(15) | 0.0371(7) |
| H2A_9 | 0.288(7) | 0.4008(19) | −0.008(3) | 0.056 |
| H2B_9 | 0.225(8) | 0.3455(12) | −0.013(3) | 0.056 |
| O3_9 | 0.8696(5) | 0.42524(15) | 0.05398(14) | 0.0377(7) |
| H3A_9 | 0.962(8) | 0.406(2) | 0.036(2) | 0.056 |
| H3B_9 | 0.874(10) | 0.416(2) | 0.0890(11) | 0.056 |
| O4_9 | 0.6564(5) | 0.39794(16) | 0.54912(13) | 0.0390(7) |
| H4A_9 | 0.541(6) | 0.385(3) | 0.538(2) | 0.058 |
| H4B_9 | 0.659(9) | 0.400(3) | 0.5845(11) | 0.058 |
| O5_9 | 0.2815(5) | 0.24588(13) | 0.46462(13) | 0.0303(6) |
| H5A_9 | 0.379(7) | 0.223(2) | 0.474(2) | 0.045 |
| H5B_9 | 0.283(9) | 0.245(2) | 0.4280(11) | 0.045 |
| O6_9 | 0.2803(6) | 0.35233(16) | 0.51804(16) | 0.0448(8) |
| H6A_9 | 0.296(10) | 0.327(2) | 0.492(2) | 0.067 |
| H6B_9 | 0.172(8) | 0.372(2) | 0.505(3) | 0.067 |

*U*_eq_ is defined as 1/3 of the trace of the orthogonalized *U_ij_* tensor.

## Table 1 Anisotropic displacement parameters [Å^2^] for TXA_Para_0m. The anisotropic displacement factor exponent takes the form: −2π^2^[ *h*^2^*(a*)*^2^*U*_11_ *+ k*^2^*(b*)*^2^*U*_22_ + … + *2hka*b*U*_12_ ]

| **Atom** | ***U*_11_** | ***U*_22_** | ***U*_33_** | ***U*_23_** | ***U*_13_** | ***U*_12_** |
| --- | --- | --- | --- | --- | --- | --- |
| O1_1 | 0.0228(13) | 0.0163(12) | 0.0284(13) | −0.0077(10) | −0.0040(10) | 0.0021(10) |
| O2_1 | 0.0206(13) | 0.0272(16) | 0.0398(15) | −0.0074(12) | 0.0037(11) | −0.0110(11) |
| N1_1 | 0.0180(13) | 0.0115(14) | 0.0234(14) | −0.0024(11) | 0.0022(11) | −0.0018(11) |
| C1_1 | 0.0180(15) | 0.0104(15) | 0.0209(15) | 0.0006(12) | −0.0031(12) | −0.0001(13) |
| C2_1 | 0.0197(16) | 0.0099(15) | 0.0182(14) | −0.0011(12) | 0.0017(12) | −0.0029(12) |
| C3_1 | 0.0191(16) | 0.0133(16) | 0.0206(15) | 0.0027(12) | −0.0011(12) | −0.0029(12) |
| C4_1 | 0.0202(16) | 0.0159(17) | 0.0225(16) | 0.0000(13) | −0.0015(13) | −0.0047(13) |
| C5_1 | 0.0199(16) | 0.0106(16) | 0.0196(15) | −0.0015(12) | 0.0013(12) | −0.0017(12) |
| C6_1 | 0.0181(16) | 0.0095(17) | 0.0302(18) | −0.0009(13) | 0.0002(13) | −0.0005(12) |
| C7_1 | 0.0177(16) | 0.0120(16) | 0.0264(17) | −0.0013(13) | −0.0039(13) | −0.0029(13) |
| C8_1 | 0.0241(17) | 0.0129(17) | 0.0230(16) | −0.0018(12) | 0.0002(13) | −0.0062(13) |
| O1_2 | 0.0191(12) | 0.0177(13) | 0.0306(13) | −0.0088(10) | −0.0031(10) | 0.0029(10) |
| O2_2 | 0.0181(13) | 0.0218(14) | 0.0417(15) | −0.0064(11) | 0.0037(11) | −0.0079(10) |
| N1_2 | 0.0189(13) | 0.0116(13) | 0.0237(13) | −0.0021(11) | 0.0013(11) | −0.0041(11) |
| C1_2 | 0.0194(16) | 0.0116(16) | 0.0203(15) | 0.0003(12) | −0.0006(12) | 0.0011(12) |
| C2_2 | 0.0196(16) | 0.0125(16) | 0.0196(15) | −0.0020(12) | 0.0010(12) | −0.0025(12) |
| C3_2 | 0.0210(17) | 0.0130(17) | 0.0316(17) | 0.0039(13) | −0.0056(14) | −0.0049(13) |
| C4_2 | 0.0256(18) | 0.0169(18) | 0.0285(18) | 0.0043(14) | −0.0097(14) | −0.0061(14) |
| C5_2 | 0.0206(17) | 0.0137(17) | 0.0200(15) | −0.0006(12) | −0.0001(13) | −0.0023(13) |
| C6_2 | 0.0222(18) | 0.0107(17) | 0.042(2) | 0.0023(15) | −0.0057(15) | −0.0006(14) |
| C7_2 | 0.0216(17) | 0.0143(17) | 0.0331(19) | 0.0021(14) | −0.0076(14) | −0.0008(13) |
| C8_2 | 0.0246(17) | 0.0140(17) | 0.0249(17) | 0.0023(13) | −0.0013(13) | −0.0063(14) |
| O1_3 | 0.0238(13) | 0.0163(13) | 0.0305(13) | −0.0078(10) | −0.0058(10) | 0.0030(10) |
| O2_3 | 0.0197(13) | 0.0269(15) | 0.0575(19) | −0.0111(13) | 0.0043(12) | −0.0081(12) |
| N1_3 | 0.0181(13) | 0.0112(14) | 0.0249(14) | −0.0017(11) | 0.0011(11) | −0.0035(11) |
| C1_3 | 0.0206(16) | 0.0130(16) | 0.0217(16) | 0.0016(13) | −0.0007(12) | −0.0024(13) |
| C2_3 | 0.0178(16) | 0.0149(16) | 0.0215(15) | −0.0006(13) | 0.0031(12) | −0.0048(13) |
| C3_3 | 0.0232(17) | 0.0139(17) | 0.0249(16) | 0.0025(12) | −0.0040(13) | −0.0047(13) |
| C4_3 | 0.0223(17) | 0.0165(17) | 0.0242(16) | 0.0013(13) | −0.0051(13) | −0.0057(13) |
| C5_3 | 0.0212(17) | 0.0107(16) | 0.0196(15) | −0.0027(12) | −0.0013(12) | −0.0033(13) |
| C6_3 | 0.0219(18) | 0.0124(18) | 0.040(2) | 0.0000(15) | −0.0050(15) | −0.0022(14) |
| C7_3 | 0.0212(17) | 0.0129(17) | 0.0335(19) | −0.0004(14) | −0.0071(14) | −0.0003(13) |
| C8_3 | 0.0281(18) | 0.0139(17) | 0.0199(15) | −0.0021(12) | −0.0018(13) | −0.0046(14) |
| N1_4 | 0.0178(13) | 0.0125(14) | 0.0250(14) | −0.0014(11) | −0.0014(11) | 0.0038(11) |
| O1_4 | 0.0230(13) | 0.0243(15) | 0.0396(15) | −0.0089(11) | −0.0048(11) | 0.0119(11) |
| O2_4 | 0.0240(13) | 0.0184(13) | 0.0268(12) | −0.0082(10) | 0.0024(10) | −0.0004(10) |
| C1_4 | 0.0200(16) | 0.0107(16) | 0.0211(15) | −0.0006(12) | 0.0018(12) | 0.0033(12) |
| C2_4 | 0.0203(16) | 0.0122(16) | 0.0164(14) | −0.0010(12) | −0.0017(12) | 0.0048(12) |
| C3_4 | 0.0204(17) | 0.0117(17) | 0.0293(17) | −0.0001(13) | 0.0064(13) | 0.0036(13) |
| C4_4 | 0.0198(17) | 0.0124(17) | 0.0302(18) | −0.0011(13) | 0.0040(13) | 0.0007(13) |
| C5_4 | 0.0186(16) | 0.0150(17) | 0.0173(15) | −0.0010(12) | 0.0014(12) | 0.0048(13) |
| C6_4 | 0.0232(17) | 0.0170(18) | 0.0217(16) | 0.0031(13) | 0.0067(13) | 0.0038(13) |
| C7_4 | 0.0241(17) | 0.0112(17) | 0.0264(16) | 0.0031(13) | 0.0047(14) | −0.0003(13) |
| C8_4 | 0.0231(17) | 0.0153(17) | 0.0242(17) | −0.0007(13) | 0.0021(13) | 0.0043(13) |
| O1_5 | 0.0231(14) | 0.0244(15) | 0.0447(16) | −0.0121(12) | −0.0044(12) | 0.0101(11) |
| O2_5 | 0.0259(13) | 0.0167(13) | 0.0276(13) | −0.0069(10) | 0.0015(10) | −0.0017(10) |
| N1_5 | 0.0183(13) | 0.0101(14) | 0.0243(14) | −0.0009(11) | 0.0022(11) | 0.0028(11) |
| C1_5 | 0.0240(17) | 0.0114(16) | 0.0232(16) | −0.0017(13) | 0.0008(13) | 0.0041(14) |
| C2_5 | 0.0180(15) | 0.0116(16) | 0.0216(16) | −0.0015(12) | −0.0018(12) | 0.0023(12) |
| C3_5 | 0.0175(16) | 0.0123(16) | 0.0292(17) | 0.0006(13) | 0.0054(13) | 0.0024(13) |
| C4_5 | 0.0244(18) | 0.0126(17) | 0.0295(18) | 0.0031(13) | 0.0052(14) | 0.0040(14) |
| C5_5 | 0.0198(16) | 0.0108(16) | 0.0197(15) | −0.0007(12) | 0.0000(12) | 0.0023(12) |
| C6_5 | 0.0204(16) | 0.0151(17) | 0.0232(16) | 0.0001(13) | 0.0044(13) | 0.0017(13) |
| C7_5 | 0.0209(17) | 0.0127(16) | 0.0224(15) | 0.0023(12) | 0.0009(13) | 0.0014(13) |
| C8_5 | 0.0219(17) | 0.0147(17) | 0.0242(16) | 0.0013(13) | 0.0029(13) | 0.0046(13) |
| O1_6 | 0.0165(12) | 0.0174(13) | 0.0507(17) | −0.0055(11) | −0.0004(11) | 0.0054(10) |
| O2_6 | 0.0231(12) | 0.0142(12) | 0.0292(13) | −0.0062(10) | 0.0058(10) | −0.0015(10) |
| N1_6 | 0.0196(14) | 0.0128(14) | 0.0261(14) | −0.0051(11) | −0.0025(11) | 0.0029(11) |
| C1_6 | 0.0218(16) | 0.0101(15) | 0.0195(15) | 0.0016(12) | 0.0023(12) | 0.0009(13) |
| C2_6 | 0.0153(15) | 0.0123(16) | 0.0195(14) | −0.0021(12) | −0.0002(12) | 0.0032(12) |
| C3_6 | 0.0201(16) | 0.0130(17) | 0.0286(17) | −0.0014(13) | 0.0069(13) | 0.0022(13) |
| C4_6 | 0.0223(18) | 0.0112(16) | 0.0332(19) | 0.0012(14) | 0.0062(14) | 0.0013(13) |
| C5_6 | 0.0181(16) | 0.0132(17) | 0.0210(15) | −0.0019(12) | −0.0005(12) | 0.0020(12) |
| C6_6 | 0.0172(15) | 0.0198(18) | 0.0262(17) | 0.0001(13) | 0.0051(13) | 0.0042(13) |
| C7_6 | 0.0214(17) | 0.0115(16) | 0.0246(16) | 0.0024(13) | 0.0023(13) | 0.0015(13) |
| C8_6 | 0.0245(17) | 0.0180(18) | 0.0225(16) | −0.0016(13) | 0.0026(13) | 0.0053(14) |
| O3_7 | 0.0257(14) | 0.0307(16) | 0.0464(17) | −0.0030(13) | −0.0054(12) | 0.0021(12) |
| O4_7 | 0.0278(14) | 0.0341(15) | 0.0349(14) | −0.0008(12) | −0.0018(11) | 0.0072(12) |
| N2_7 | 0.0236(15) | 0.0291(18) | 0.0286(15) | 0.0020(13) | 0.0016(12) | 0.0012(13) |
| C9_7 | 0.0254(18) | 0.030(2) | 0.0202(16) | −0.0033(14) | 0.0029(14) | 0.0049(15) |
| C10_7 | 0.0224(18) | 0.033(2) | 0.0294(18) | −0.0090(15) | −0.0041(14) | −0.0007(15) |
| C11_7 | 0.0206(17) | 0.030(2) | 0.0297(19) | −0.0088(15) | −0.0003(14) | 0.0044(15) |
| C12_7 | 0.0228(17) | 0.0272(19) | 0.0215(16) | −0.0030(14) | 0.0003(13) | 0.0042(15) |
| C13_7 | 0.0210(17) | 0.0241(19) | 0.0289(18) | −0.0024(14) | −0.0006(14) | 0.0010(14) |
| C14_7 | 0.0248(18) | 0.029(2) | 0.0309(18) | −0.0022(16) | −0.0012(15) | 0.0049(16) |
| C15_7 | 0.033(2) | 0.0240(19) | 0.0179(15) | 0.0057(13) | −0.0007(14) | 0.0061(16) |
| C16_7 | 0.031(2) | 0.028(2) | 0.042(2) | 0.0091(17) | 0.0045(18) | 0.0054(17) |
| O3_8 | 0.0334(15) | 0.0228(14) | 0.0358(15) | −0.0054(11) | 0.0053(12) | −0.0006(11) |
| O4_8 | 0.0249(14) | 0.0303(15) | 0.0461(16) | −0.0036(12) | −0.0028(12) | 0.0002(11) |
| N2_8 | 0.0277(16) | 0.0269(17) | 0.0308(16) | 0.0046(13) | −0.0022(13) | −0.0004(13) |
| C9_8 | 0.0297(19) | 0.0253(19) | 0.0187(16) | −0.0001(13) | −0.0005(14) | 0.0009(15) |
| C10_8 | 0.0273(18) | 0.027(2) | 0.0262(17) | −0.0027(14) | 0.0031(14) | 0.0018(15) |
| C11_8 | 0.0222(17) | 0.033(2) | 0.0234(17) | −0.0016(15) | 0.0027(13) | −0.0039(16) |
| C12_8 | 0.0251(18) | 0.0262(19) | 0.0209(16) | 0.0024(14) | −0.0031(14) | −0.0010(14) |
| C13_8 | 0.0245(18) | 0.035(2) | 0.0244(17) | 0.0005(15) | 0.0035(14) | 0.0029(15) |
| C14_8 | 0.0252(18) | 0.031(2) | 0.0259(17) | 0.0021(15) | 0.0039(15) | 0.0015(15) |
| C15_8 | 0.0283(18) | 0.0247(19) | 0.0199(16) | −0.0019(14) | 0.0020(14) | 0.0013(15) |
| C16_8 | 0.037(2) | 0.027(2) | 0.031(2) | 0.0033(16) | 0.0020(17) | −0.0041(17) |
| O1_9 | 0.0269(14) | 0.0305(15) | 0.0399(16) | −0.0060(12) | 0.0055(12) | −0.0041(11) |
| O2_9 | 0.0282(15) | 0.0322(17) | 0.0510(18) | −0.0065(14) | 0.0019(14) | 0.0010(12) |
| O3_9 | 0.0298(15) | 0.0393(17) | 0.0439(17) | 0.0043(14) | 0.0010(13) | 0.0102(13) |
| O4_9 | 0.0337(16) | 0.049(2) | 0.0339(15) | −0.0063(14) | 0.0027(13) | −0.0162(14) |
| O5_9 | 0.0245(14) | 0.0284(15) | 0.0381(15) | −0.0002(12) | −0.0012(12) | 0.0026(11) |
| O6_9 | 0.0370(18) | 0.0382(19) | 0.059(2) | −0.0152(15) | −0.0106(16) | 0.0004(14) |

## Table 1 Bond lengths and angles for TXA_Para_0m

| **Atom–Atom** | **Length [Å]** |
| --- | --- |
| O1_1–C1_1 | 1.262(4) |
| O2_1–C1_1 | 1.257(5) |
| N1_1–C8_1 | 1.499(4) |
| C1_1–C2_1 | 1.531(4) |
| C2_1–C3_1 | 1.524(5) |
| C2_1–C7_1 | 1.538(5) |
| C3_1–C4_1 | 1.534(5) |
| C4_1–C5_1 | 1.528(5) |
| C5_1–C8_1 | 1.521(5) |
| C5_1–C6_1 | 1.534(5) |
| C6_1–C7_1 | 1.530(5) |
| O1_2–C1_2 | 1.255(4) |
| O2_2–C1_2 | 1.255(5) |
| N1_2–C8_2 | 1.497(4) |
| C1_2–C2_2 | 1.540(5) |
| C2_2–C7_2 | 1.527(5) |
| C2_2–C3_2 | 1.527(5) |
| C3_2–C4_2 | 1.529(5) |
| C4_2–C5_2 | 1.530(5) |
| C5_2–C8_2 | 1.522(5) |
| C5_2–C6_2 | 1.524(5) |
| C6_2–C7_2 | 1.533(5) |
| O1_3–C1_3 | 1.269(5) |
| O2_3–C1_3 | 1.244(5) |
| N1_3–C8_3 | 1.496(4) |
| C1_3–C2_3 | 1.530(5) |
| C2_3–C7_3 | 1.531(5) |
| C2_3–C3_3 | 1.534(5) |
| C3_3–C4_3 | 1.525(5) |
| C4_3–C5_3 | 1.527(5) |
| C5_3–C8_3 | 1.519(5) |
| C5_3–C6_3 | 1.540(5) |
| C6_3–C7_3 | 1.530(5) |
| N1_4–C8_4 | 1.494(5) |
| O1_4–C1_4 | 1.258(5) |
| O2_4–C1_4 | 1.262(4) |
| C1_4–C2_4 | 1.526(5) |
| C2_4–C7_4 | 1.527(5) |
| C2_4–C3_4 | 1.544(5) |
| C3_4–C4_4 | 1.523(5) |
| C4_4–C5_4 | 1.532(5) |
| C5_4–C8_4 | 1.524(5) |
| C5_4–C6_4 | 1.526(5) |
| C6_4–C7_4 | 1.522(5) |
| O1_5–C1_5 | 1.248(5) |
| O2_5–C1_5 | 1.261(5) |
| N1_5–C8_5 | 1.491(4) |
| C1_5–C2_5 | 1.533(5) |
| C2_5–C7_5 | 1.530(5) |
| C2_5–C3_5 | 1.542(5) |
| C3_5–C4_5 | 1.536(5) |
| C4_5–C5_5 | 1.532(5) |
| C5_5–C6_5 | 1.528(5) |
| C5_5–C8_5 | 1.539(5) |
| C6_5–C7_5 | 1.537(5) |
| O1_6–C1_6 | 1.247(5) |
| O2_6–C1_6 | 1.267(5) |
| N1_6–C8_6 | 1.496(5) |
| C1_6–C2_6 | 1.529(5) |
| C2_6–C7_6 | 1.527(5) |
| C2_6–C3_6 | 1.529(5) |
| C3_6–C4_6 | 1.533(5) |
| C4_6–C5_6 | 1.536(5) |
| C5_6–C8_6 | 1.522(5) |
| C5_6–C6_6 | 1.528(5) |
| C6_6–C7_6 | 1.525(5) |
| O3_7–C9_7 | 1.358(5) |
| O4_7–C15_7 | 1.224(5) |
| N2_7–C15_7 | 1.356(5) |
| N2_7–C12_7 | 1.421(5) |
| C9_7–C10_7 | 1.393(5) |
| C9_7–C14_7 | 1.399(6) |
| C10_7–C11_7 | 1.391(6) |
| C11_7–C12_7 | 1.391(5) |
| C12_7–C13_7 | 1.395(5) |
| C13_7–C14_7 | 1.393(6) |
| C15_7–C16_7 | 1.508(6) |
| O3_8–C9_8 | 1.373(5) |
| O4_8–C15_8 | 1.230(5) |
| N2_8–C15_8 | 1.343(5) |
| N2_8–C12_8 | 1.426(5) |
| C9_8–C10_8 | 1.386(6) |
| C9_8–C14_8 | 1.389(6) |
| C10_8–C11_8 | 1.387(6) |
| C11_8–C12_8 | 1.401(6) |
| C12_8–C13_8 | 1.386(6) |
| C13_8–C14_8 | 1.389(6) |
| C15_8–C16_8 | 1.502(6) |
|  |  |
| **Atom–Atom–Atom** | **Angle [°]** |
| O2_1–C1_1–O1_1 | 123.6(3) |
| O2_1–C1_1–C2_1 | 118.1(3) |
| O1_1–C1_1–C2_1 | 118.2(3) |
| C3_1–C2_1–C1_1 | 113.0(3) |
| C3_1–C2_1–C7_1 | 110.6(3) |
| C1_1–C2_1–C7_1 | 107.7(3) |
| C2_1–C3_1–C4_1 | 112.1(3) |
| C5_1–C4_1–C3_1 | 110.6(3) |
| C8_1–C5_1–C4_1 | 110.7(3) |
| C8_1–C5_1–C6_1 | 113.2(3) |
| C4_1–C5_1–C6_1 | 110.3(3) |
| C7_1–C6_1–C5_1 | 110.1(3) |
| C6_1–C7_1–C2_1 | 111.4(3) |
| N1_1–C8_1–C5_1 | 111.9(3) |
| O2_2–C1_2–O1_2 | 124.7(3) |
| O2_2–C1_2–C2_2 | 116.2(3) |
| O1_2–C1_2–C2_2 | 119.0(3) |
| C7_2–C2_2–C3_2 | 110.5(3) |
| C7_2–C2_2–C1_2 | 108.5(3) |
| C3_2–C2_2–C1_2 | 112.9(3) |
| C2_2–C3_2–C4_2 | 112.1(3) |
| C3_2–C4_2–C5_2 | 110.5(3) |
| C8_2–C5_2–C6_2 | 113.3(3) |
| C8_2–C5_2–C4_2 | 110.9(3) |
| C6_2–C5_2–C4_2 | 109.2(3) |
| C5_2–C6_2–C7_2 | 111.0(3) |
| C2_2–C7_2–C6_2 | 111.5(3) |
| N1_2–C8_2–C5_2 | 111.8(3) |
| O2_3–C1_3–O1_3 | 124.5(3) |
| O2_3–C1_3–C2_3 | 117.3(3) |
| O1_3–C1_3–C2_3 | 118.0(3) |
| C1_3–C2_3–C7_3 | 108.1(3) |
| C1_3–C2_3–C3_3 | 113.6(3) |
| C7_3–C2_3–C3_3 | 110.8(3) |
| C4_3–C3_3–C2_3 | 112.3(3) |
| C3_3–C4_3–C5_3 | 110.7(3) |
| C8_3–C5_3–C4_3 | 111.3(3) |
| C8_3–C5_3–C6_3 | 113.2(3) |
| C4_3–C5_3–C6_3 | 109.6(3) |
| C7_3–C6_3–C5_3 | 110.2(3) |
| C6_3–C7_3–C2_3 | 111.7(3) |
| N1_3–C8_3–C5_3 | 111.7(3) |
| O1_4–C1_4–O2_4 | 124.4(3) |
| O1_4–C1_4–C2_4 | 117.3(3) |
| O2_4–C1_4–C2_4 | 118.2(3) |
| C1_4–C2_4–C7_4 | 112.9(3) |
| C1_4–C2_4–C3_4 | 108.7(3) |
| C7_4–C2_4–C3_4 | 110.3(3) |
| C4_4–C3_4–C2_4 | 111.7(3) |
| C3_4–C4_4–C5_4 | 111.0(3) |
| C8_4–C5_4–C6_4 | 110.9(3) |
| C8_4–C5_4–C4_4 | 113.3(3) |
| C6_4–C5_4–C4_4 | 109.3(3) |
| C7_4–C6_4–C5_4 | 110.8(3) |
| C6_4–C7_4–C2_4 | 112.5(3) |
| N1_4–C8_4–C5_4 | 112.2(3) |
| O1_5–C1_5–O2_5 | 125.0(3) |
| O1_5–C1_5–C2_5 | 117.0(3) |
| O2_5–C1_5–C2_5 | 118.0(3) |
| C7_5–C2_5–C1_5 | 113.1(3) |
| C7_5–C2_5–C3_5 | 110.1(3) |
| C1_5–C2_5–C3_5 | 108.2(3) |
| C4_5–C3_5–C2_5 | 111.3(3) |
| C5_5–C4_5–C3_5 | 110.4(3) |
| C6_5–C5_5–C4_5 | 110.2(3) |
| C6_5–C5_5–C8_5 | 110.5(3) |
| C4_5–C5_5–C8_5 | 112.5(3) |
| C5_5–C6_5–C7_5 | 109.7(3) |
| C2_5–C7_5–C6_5 | 111.6(3) |
| N1_5–C8_5–C5_5 | 111.2(3) |
| O1_6–C1_6–O2_6 | 124.5(3) |
| O1_6–C1_6–C2_6 | 116.5(3) |
| O2_6–C1_6–C2_6 | 118.9(3) |
| C7_6–C2_6–C3_6 | 110.3(3) |
| C7_6–C2_6–C1_6 | 113.4(3) |
| C3_6–C2_6–C1_6 | 108.7(3) |
| C2_6–C3_6–C4_6 | 112.2(3) |
| C3_6–C4_6–C5_6 | 110.9(3) |
| C8_6–C5_6–C6_6 | 110.8(3) |
| C8_6–C5_6–C4_6 | 112.9(3) |
| C6_6–C5_6–C4_6 | 109.3(3) |
| C7_6–C6_6–C5_6 | 110.9(3) |
| C6_6–C7_6–C2_6 | 112.5(3) |
| N1_6–C8_6–C5_6 | 111.8(3) |
| C15_7–N2_7–C12_7 | 127.5(3) |
| O3_7–C9_7–C10_7 | 118.7(4) |
| O3_7–C9_7–C14_7 | 122.1(4) |
| C10_7–C9_7–C14_7 | 119.1(4) |
| C11_7–C10_7–C9_7 | 121.2(4) |
| C12_7–C11_7–C10_7 | 119.6(4) |
| C11_7–C12_7–C13_7 | 119.6(4) |
| C11_7–C12_7–N2_7 | 123.7(3) |
| C13_7–C12_7–N2_7 | 116.7(3) |
| C14_7–C13_7–C12_7 | 120.7(4) |
| C13_7–C14_7–C9_7 | 119.7(4) |
| O4_7–C15_7–N2_7 | 123.5(4) |
| O4_7–C15_7–C16_7 | 121.2(4) |
| N2_7–C15_7–C16_7 | 115.2(4) |
| C15_8–N2_8–C12_8 | 127.1(3) |
| O3_8–C9_8–C10_8 | 117.7(4) |
| O3_8–C9_8–C14_8 | 122.5(4) |
| C10_8–C9_8–C14_8 | 119.9(4) |
| C9_8–C10_8–C11_8 | 120.2(4) |
| C10_8–C11_8–C12_8 | 120.2(4) |
| C13_8–C12_8–C11_8 | 119.2(4) |
| C13_8–C12_8–N2_8 | 117.8(4) |
| C11_8–C12_8–N2_8 | 123.0(4) |
| C12_8–C13_8–C14_8 | 120.5(4) |
| C9_8–C14_8–C13_8 | 120.0(4) |
| O4_8–C15_8–N2_8 | 122.9(4) |
| O4_8–C15_8–C16_8 | 122.0(4) |
| N2_8–C15_8–C16_8 | 115.1(4) |

Bonds and angles to hydrogen atoms were omitted.

## Table 1 Torsion angles for TXA_Para_0m

| **Atom–Atom–Atom–Atom** | **Torsion Angle [°]** |
| --- | --- |
| O2_1–C1_1–C2_1–C3_1 | −150.8(3) |
| O1_1–C1_1–C2_1–C3_1 | 31.8(4) |
| O2_1–C1_1–C2_1–C7_1 | 86.7(4) |
| O1_1–C1_1–C2_1–C7_1 | −90.7(4) |
| C1_1–C2_1–C3_1–C4_1 | −174.9(3) |
| C7_1–C2_1–C3_1–C4_1 | −54.1(4) |
| C2_1–C3_1–C4_1–C5_1 | 55.8(4) |
| C3_1–C4_1–C5_1–C8_1 | 176.5(3) |
| C3_1–C4_1–C5_1–C6_1 | −57.4(4) |
| C8_1–C5_1–C6_1–C7_1 | −176.9(3) |
| C4_1–C5_1–C6_1–C7_1 | 58.5(4) |
| C5_1–C6_1–C7_1–C2_1 | −57.3(4) |
| C3_1–C2_1–C7_1–C6_1 | 55.0(4) |
| C1_1–C2_1–C7_1–C6_1 | 178.9(3) |
| C4_1–C5_1–C8_1–N1_1 | −169.0(3) |
| C6_1–C5_1–C8_1–N1_1 | 66.6(4) |
| O2_2–C1_2–C2_2–C7_2 | 80.8(4) |
| O1_2–C1_2–C2_2–C7_2 | −96.4(4) |
| O2_2–C1_2–C2_2–C3_2 | −156.3(3) |
| O1_2–C1_2–C2_2–C3_2 | 26.5(4) |
| C7_2–C2_2–C3_2–C4_2 | −54.0(4) |
| C1_2–C2_2–C3_2–C4_2 | −175.7(3) |
| C2_2–C3_2–C4_2–C5_2 | 56.9(4) |
| C3_2–C4_2–C5_2–C8_2 | 176.0(3) |
| C3_2–C4_2–C5_2–C6_2 | −58.4(4) |
| C8_2–C5_2–C6_2–C7_2 | −177.0(3) |
| C4_2–C5_2–C6_2–C7_2 | 58.9(4) |
| C3_2–C2_2–C7_2–C6_2 | 53.8(4) |
| C1_2–C2_2–C7_2–C6_2 | 178.0(3) |
| C5_2–C6_2–C7_2–C2_2 | −57.2(4) |
| C6_2–C5_2–C8_2–N1_2 | 67.4(4) |
| C4_2–C5_2–C8_2–N1_2 | −169.4(3) |
| O2_3–C1_3–C2_3–C7_3 | 76.3(4) |
| O1_3–C1_3–C2_3–C7_3 | −99.6(4) |
| O2_3–C1_3–C2_3–C3_3 | −160.3(3) |
| O1_3–C1_3–C2_3–C3_3 | 23.8(5) |
| C1_3–C2_3–C3_3–C4_3 | −174.9(3) |
| C7_3–C2_3–C3_3–C4_3 | −52.9(4) |
| C2_3–C3_3–C4_3–C5_3 | 56.0(4) |
| C3_3–C4_3–C5_3–C8_3 | 175.5(3) |
| C3_3–C4_3–C5_3–C6_3 | −58.5(4) |
| C8_3–C5_3–C6_3–C7_3 | −175.9(3) |
| C4_3–C5_3–C6_3–C7_3 | 59.2(4) |
| C5_3–C6_3–C7_3–C2_3 | −57.3(4) |
| C1_3–C2_3–C7_3–C6_3 | 178.7(3) |
| C3_3–C2_3–C7_3–C6_3 | 53.6(4) |
| C4_3–C5_3–C8_3–N1_3 | −169.9(3) |
| C6_3–C5_3–C8_3–N1_3 | 66.2(4) |
| O1_4–C1_4–C2_4–C7_4 | −150.3(3) |
| O2_4–C1_4–C2_4–C7_4 | 33.3(4) |
| O1_4–C1_4–C2_4–C3_4 | 87.0(4) |
| O2_4–C1_4–C2_4–C3_4 | −89.4(4) |
| C1_4–C2_4–C3_4–C4_4 | 177.3(3) |
| C7_4–C2_4–C3_4–C4_4 | 53.1(4) |
| C2_4–C3_4–C4_4–C5_4 | −56.7(4) |
| C3_4–C4_4–C5_4–C8_4 | −177.1(3) |
| C3_4–C4_4–C5_4–C6_4 | 58.7(4) |
| C8_4–C5_4–C6_4–C7_4 | 176.1(3) |
| C4_4–C5_4–C6_4–C7_4 | −58.3(4) |
| C5_4–C6_4–C7_4–C2_4 | 56.8(4) |
| C1_4–C2_4–C7_4–C6_4 | −175.0(3) |
| C3_4–C2_4–C7_4–C6_4 | −53.2(4) |
| C6_4–C5_4–C8_4–N1_4 | −168.3(3) |
| C4_4–C5_4–C8_4–N1_4 | 68.4(4) |
| O1_5–C1_5–C2_5–C7_5 | −149.4(3) |
| O2_5–C1_5–C2_5–C7_5 | 33.6(5) |
| O1_5–C1_5–C2_5–C3_5 | 88.3(4) |
| O2_5–C1_5–C2_5–C3_5 | −88.7(4) |
| C7_5–C2_5–C3_5–C4_5 | 54.9(4) |
| C1_5–C2_5–C3_5–C4_5 | 179.0(3) |
| C2_5–C3_5–C4_5–C5_5 | −56.6(4) |
| C3_5–C4_5–C5_5–C6_5 | 58.6(4) |
| C3_5–C4_5–C5_5–C8_5 | −177.6(3) |
| C4_5–C5_5–C6_5–C7_5 | −59.0(4) |
| C8_5–C5_5–C6_5–C7_5 | 176.1(3) |
| C1_5–C2_5–C7_5–C6_5 | −177.0(3) |
| C3_5–C2_5–C7_5–C6_5 | −55.8(4) |
| C5_5–C6_5–C7_5–C2_5 | 58.2(4) |
| C6_5–C5_5–C8_5–N1_5 | −170.0(3) |
| C4_5–C5_5–C8_5–N1_5 | 66.4(4) |
| O1_6–C1_6–C2_6–C7_6 | −163.7(3) |
| O2_6–C1_6–C2_6–C7_6 | 19.6(4) |
| O1_6–C1_6–C2_6–C3_6 | 73.3(4) |
| O2_6–C1_6–C2_6–C3_6 | −103.4(4) |
| C7_6–C2_6–C3_6–C4_6 | 53.2(4) |
| C1_6–C2_6–C3_6–C4_6 | 178.0(3) |
| C2_6–C3_6–C4_6–C5_6 | −56.3(4) |
| C3_6–C4_6–C5_6–C8_6 | −178.5(3) |
| C3_6–C4_6–C5_6–C6_6 | 57.7(4) |
| C8_6–C5_6–C6_6–C7_6 | 177.1(3) |
| C4_6–C5_6–C6_6–C7_6 | −57.9(4) |
| C5_6–C6_6–C7_6–C2_6 | 56.9(4) |
| C3_6–C2_6–C7_6–C6_6 | −53.5(4) |
| C1_6–C2_6–C7_6–C6_6 | −175.6(3) |
| C6_6–C5_6–C8_6–N1_6 | −171.3(3) |
| C4_6–C5_6–C8_6–N1_6 | 65.8(4) |
| O3_7–C9_7–C10_7–C11_7 | −179.0(4) |
| C14_7–C9_7–C10_7–C11_7 | −0.2(6) |
| C9_7–C10_7–C11_7–C12_7 | −2.1(6) |
| C10_7–C11_7–C12_7–C13_7 | 3.0(6) |
| C10_7–C11_7–C12_7–N2_7 | −175.6(4) |
| C15_7–N2_7–C12_7–C11_7 | 19.7(6) |
| C15_7–N2_7–C12_7–C13_7 | −159.0(4) |
| C11_7–C12_7–C13_7–C14_7 | −1.7(6) |
| N2_7–C12_7–C13_7–C14_7 | 177.1(3) |
| C12_7–C13_7–C14_7–C9_7 | −0.7(6) |
| O3_7–C9_7–C14_7–C13_7 | −179.7(4) |
| C10_7–C9_7–C14_7–C13_7 | 1.6(6) |
| C12_7–N2_7–C15_7–O4_7 | −8.9(6) |
| C12_7–N2_7–C15_7–C16_7 | 170.2(4) |
| O3_8–C9_8–C10_8–C11_8 | −177.7(3) |
| C14_8–C9_8–C10_8–C11_8 | 0.8(6) |
| C9_8–C10_8–C11_8–C12_8 | 0.2(6) |
| C10_8–C11_8–C12_8–C13_8 | −1.4(6) |
| C10_8–C11_8–C12_8–N2_8 | −177.9(3) |
| C15_8–N2_8–C12_8–C13_8 | 161.2(4) |
| C15_8–N2_8–C12_8–C11_8 | −22.2(6) |
| C11_8–C12_8–C13_8–C14_8 | 1.6(6) |
| N2_8–C12_8–C13_8–C14_8 | 178.3(3) |
| O3_8–C9_8–C14_8–C13_8 | 177.8(3) |
| C10_8–C9_8–C14_8–C13_8 | −0.6(6) |
| C12_8–C13_8–C14_8–C9_8 | −0.7(6) |
| C12_8–N2_8–C15_8–O4_8 | −7.9(6) |
| C12_8–N2_8–C15_8–C16_8 | 170.8(3) |

Bonds and angles to hydrogen atoms were omitted.

## Table 1 Hydrogen bonds for TXA_Para_0m

| **D–H⋯A [Å]** | **d(D–H) [Å]** | **d(H⋯A) [Å]** | **d(D⋯A) [Å]** | **<(DHA) [°]** |
| --- | --- | --- | --- | --- |
| N1_1–H1A_1⋯O2_2^#1^ | 0.91 | 1.83 | 2.736(4) | 170.3 |
| N1_1–H1B_1⋯O2_6^#1^ | 0.91 | 1.86 | 2.758(4) | 170.4 |
| N1_1–H1C_1⋯O1_2 | 0.91 | 1.94 | 2.836(4) | 168.3 |
| N1_2–H1A_2⋯O2_3^#1^ | 0.91 | 1.79 | 2.702(4) | 177.6 |
| N1_2–H1B_2⋯O2_5^#1^ | 0.91 | 1.87 | 2.780(4) | 173.1 |
| N1_2–H1C_2⋯O1_3 | 0.91 | 1.87 | 2.774(4) | 173.1 |
| N1_3–H1A_3⋯O2_1^#2^ | 0.91 | 1.85 | 2.755(4) | 171.4 |
| N1_3–H1B_3⋯O2_4^#1^ | 0.91 | 1.86 | 2.771(4) | 176.6 |
| N1_3–H1C_3⋯O1_1^#3^ | 0.91 | 1.94 | 2.846(4) | 173.0 |
| N1_4–H1A_4⋯O1_3 | 0.91 | 1.85 | 2.759(4) | 175.2 |
| N1_4–H1B_4⋯O2_5 | 0.91 | 1.94 | 2.842(4) | 170.5 |
| N1_4–H1C_4⋯O1_5^#1^ | 0.91 | 1.88 | 2.780(4) | 170.6 |
| N1_5–H1A_5⋯O1_2 | 0.91 | 1.91 | 2.813(4) | 174.3 |
| N1_5–H1B_5⋯O2_6 | 0.91 | 1.89 | 2.793(4) | 171.4 |
| N1_5–H1C_5⋯O1_6^#1^ | 0.91 | 1.79 | 2.700(4) | 178.2 |
| N1_6–H1A_6⋯O1_1 | 0.91 | 1.88 | 2.779(4) | 167.8 |
| N1_6–H1B_6⋯O2_4^#4^ | 0.91 | 1.93 | 2.826(4) | 169.3 |
| N1_6–H1C_6⋯O1_4^#5^ | 0.91 | 1.83 | 2.724(4) | 167.7 |
| O3_7–H3A_7⋯O3_9 | 0.84 | 1.76 | 2.596(4) | 172.0 |
| N2_7–H2_7⋯O1_9^#6^ | 0.88 | 2.24 | 2.973(5) | 140.4 |
| C11_7–H11_7⋯O4_7 | 0.95 | 2.31 | 2.872(5) | 117.0 |
| C14_7–H14_7⋯O3_9 | 0.95 | 2.65 | 3.289(5) | 125.5 |
| O3_8–H3A_8⋯O4_9 | 0.84 | 1.76 | 2.565(4) | 158.6 |
| N2_8–H2_8⋯O5_9^#7^ | 0.88 | 2.16 | 2.964(5) | 152.4 |
| C11_8–H11_8⋯O4_8 | 0.95 | 2.35 | 2.884(5) | 115.0 |
| C16_8–H16A_8⋯O5_9^#7^ | 0.98 | 2.42 | 3.299(6) | 148.5 |
| O1_9–H1A_9⋯O4_7^#8^ | 0.85(3) | 1.87(3) | 2.685(4) | 161(5) |
| O1_9–H1B_9⋯O2_1^#9^ | 0.85(2) | 1.91(3) | 2.763(4) | 174(6) |
| O2_9–H2A_9⋯O3_7 | 0.88(3) | 1.94(3) | 2.810(4) | 170(6) |
| O2_9–H2B_9⋯O1_9 | 0.86(3) | 2.03(3) | 2.835(4) | 154(5) |
| O3_9–H3A_9⋯O2_9^#10^ | 0.85(3) | 1.86(3) | 2.699(5) | 168(6) |
| O3_9–H3B_9⋯O2_2 | 0.86(3) | 1.96(3) | 2.778(4) | 159(5) |
| O4_9–H4A_9⋯O6_9 | 0.83(3) | 1.88(3) | 2.714(5) | 177(6) |
| O4_9–H4B_9⋯O1_4^#11^ | 0.84(3) | 1.94(3) | 2.762(4) | 166(6) |
| O5_9–H5A_9⋯O4_8^#12^ | 0.84(3) | 1.84(3) | 2.678(4) | 176(5) |
| O5_9–H5B_9⋯O1_5^#1^ | 0.87(2) | 1.93(3) | 2.787(4) | 170(6) |
| O6_9–H6A_9⋯O5_9 | 0.86(3) | 2.02(4) | 2.801(4) | 151(6) |
| O6_9–H6B_9⋯O3_8^#1^ | 0.88(3) | 2.01(3) | 2.874(5) | 166(6) |

Symmetry transformations used to generate equivalent atoms:

#1: -1+X, +Y, +Z; #2: -1+X, -1+Y, +Z; #3: +X, -1+Y, +Z; #4: +X, 1+Y, +Z; #5: -1+X, 1+Y, +Z; #6: 1-X, 0.5+Y, -Z; #7: 1-X, 0.5+Y, 1-Z; #8: -X, -0.5+Y, -Z; #9: 1-X, -0.5+Y, -Z; #10: 1+X, +Y, +Z; #11: 2-X, 0.5+Y, 1-Z; #12: 2-X, -0.5+Y, 1-Z;

## Bibliography

[1]	Bruker, *SAINT*, *V8.40B*, Bruker AXS Inc., Madison, Wisconsin, USA.

[2]	L. Krause, R. Herbst-Irmer, G. M. Sheldrick, D. Stalke, *J. Appl. Cryst.* **2015**, *48*, 3–10, doi:10.1107/S1600576714022985.

[3]	G. M. Sheldrick, *Acta Cryst.* **2015**, *A71*, 3–8, doi:10.1107/S2053273314026370.

[4]	G. M. Sheldrick, *Acta Cryst.* **2015**, *C71*, 3–8, doi:10.1107/S2053229614024218.

[5]	C. R. Groom, I. J. Bruno, M. P. Lightfoot, S. C. Ward, *Acta Cryst.* **2016**, *B72*, 171–179, doi:10.1107/S2052520616003954.

[6]	D. Kratzert, *FinalCif*, *V144*, https://dkratzert.de/finalcif.html.
