# Supplementary material for: Antifibrinolytic Agent: Synthesis and Characterization of Cocrystal and Salts of Tranexamic Acid
Source: ACS Omega. 2026 Oct 22;11(43):66176–200. doi: 10.1021/acsomega.6c03598 (PMC13737987; doi:10.1021/acsomega.6c03598)
Supplement: Supplementary file 3 [file ao6c03598_si_002.zip › report_TXA_Adipic_0m_complete-finalcif.docx]

# Structure Tables

A colourless, plate-shaped crystal was mounted on the goniometer. Data for TXA_Adipic_0m were collected from a shock-cooled single crystal at 150(2) K on a Bruker AXS D8 Quest four circle diffractometer with an I-mu-S 3.0 microsource X-ray tube using a HELIOS multilayer Montel optics as monochromator and a PhotonIII_C14 charge-integrating and photon counting pixel array detector. The diffractometer used Cu*K_α_* radiation (λ = 1.54178 Å). All data were integrated with SAINT V8.40B and a multi-scan absorption correction using SADABS 2016/2 was applied.^[1,2]^ The structure was solved by dual methods with SHELXT and refined by full-matrix least-squares methods against *F*^2^ using SHELXL-2019/2.^[3,4]^ All non-hydrogen atoms were refined with anisotropic displacement parameters. All hydrogen atoms were refined with isotropic displacement parameters. Some of their coordinates were refined freely and some on calculated positions using a riding model with their *U*_iso_ values constrained to 1.5 times the *U*_eq_ of their pivot atoms for terminal sp^3^ carbon atoms and 1.2 times for all other carbon atoms. Crystallographic data for the structures reported in this paper have been deposited with the Cambridge Crystallographic Data Centre.^[5]^ CCDC ?????? contain the supplementary crystallographic data for this paper. These data can be obtained free of charge from The Cambridge Crystallographic Data Centre via www.ccdc.cam.ac.uk/‌structures. This report and the CIF file were generated using FinalCif.^[6]^

## Table 1 Crystal data and structure refinement for TXA_Adipic_0m

| CCDC number |  |
| --- | --- |
| Empirical formula | C_22_H_40_N_2_O_8_ |
| Formula weight | 460.56 |
| Temperature [K] | 150(2) |
| Crystal system | monoclinic |
| Space group (number) | $P2_{1}/n$ (14) |
| *a* [Å] | 11.2743(12) |
| *b* [Å] | 5.6403(7) |
| *c* [Å] | 19.231(2) |
| α [°] | 90 |
| β [°] | 100.842(8) |
| γ [°] | 90 |
| Volume [Å^3^] | 1201.1(2) |
| *Z* | 2 |
| *ρ*_calc_ [gcm^−3^] | 1.273 |
| *μ* [mm^−1^] | 0.796 |
| *F*(000) | 500 |
| Crystal size [mm^3^] | 0.030×0.140×0.190 |
| Crystal colour | colourless |
| Crystal shape | plate |
| Radiation | Cu*K_α_* (λ=1.54178 Å) |
| 2θ range [°] | 8.46 to 159.85 (0.78 Å) |
| Index ranges | −14 ≤ h ≤ 13 −7 ≤ k ≤ 6 −20 ≤ l ≤ 24 |
| Reflections collected | 10133 |
| Independent reflections | 2564  *R*_int_ = 0.0366 *R*_sigma_ = 0.0328 |
| Completeness to  θ = 67.679° | 99.9 % |
| Data / Restraints / Parameters | 2564 / 0 / 157 |
| Absorption correction T_min_/T_max_ (method) | 0.6324 / 0.7543  (multi-scan) |
| Goodness-of-fit on *F*^2^ | 1.072 |
| Final *R* indexes  [*I*≥2σ(*I*)] | *R*_1_ = 0.0349 w*R*_2_ = 0.0950 |
| Final *R* indexes  [all data] | *R*_1_ = 0.0392 w*R*_2_ = 0.0973 |
| Largest peak/hole [eÅ^−3^] | 0.29/−0.21 |

## Table 1 Atomic coordinates and *U_eq_* [Å^2^] for TXA_Adipic_0m

| **Atom** | ***x*** | ***y*** | ***z*** | ***U*_eq_** |
| --- | --- | --- | --- | --- |
| O1 | 0.17648(7) | 0.56154(18) | 0.41573(4) | 0.0309(2) |
| O2 | 0.31656(7) | 0.49692(15) | 0.51259(4) | 0.02262(19) |
| H2A | 0.2578(14) | 0.545(3) | 0.5389(8) | 0.034 |
| O3 | 0.18934(7) | 0.61636(13) | 0.60283(4) | 0.02074(18) |
| O4 | 0.10378(7) | 0.88676(14) | 0.66059(4) | 0.02299(19) |
| N1 | 0.55175(9) | 0.14415(17) | 0.18133(5) | 0.0182(2) |
| H1A | 0.6014(14) | 0.059(3) | 0.1571(8) | 0.027 |
| H1B | 0.5628(14) | 0.300(3) | 0.1723(8) | 0.027 |
| H1C | 0.4750(14) | 0.101(3) | 0.1608(8) | 0.027 |
| C1 | 0.27787(10) | 0.50360(19) | 0.44359(5) | 0.0187(2) |
| C2 | 0.37494(9) | 0.42971(19) | 0.40308(5) | 0.0177(2) |
| H2 | 0.450738 | 0.516727 | 0.423604 | 0.021 |
| C3 | 0.34171(10) | 0.4930(2) | 0.32483(6) | 0.0226(2) |
| H3A | 0.326476 | 0.665545 | 0.319759 | 0.027 |
| H3B | 0.266633 | 0.409063 | 0.303288 | 0.027 |
| C4 | 0.44328(11) | 0.4240(2) | 0.28597(6) | 0.0231(2) |
| H4A | 0.418832 | 0.462787 | 0.235075 | 0.028 |
| H4B | 0.516571 | 0.517142 | 0.305159 | 0.028 |
| C5 | 0.47225(9) | 0.15954(19) | 0.29418(5) | 0.0174(2) |
| H5 | 0.398964 | 0.068931 | 0.271373 | 0.021 |
| C6 | 0.50160(10) | 0.09097(19) | 0.37263(5) | 0.0196(2) |
| H6A | 0.577737 | 0.169162 | 0.395247 | 0.023 |
| H6B | 0.514052 | −0.082598 | 0.376717 | 0.023 |
| C7 | 0.40064(10) | 0.16262(19) | 0.41188(6) | 0.0210(2) |
| H7A | 0.424981 | 0.124065 | 0.462796 | 0.025 |
| H7B | 0.326406 | 0.072144 | 0.392795 | 0.025 |
| C8 | 0.57674(9) | 0.08768(19) | 0.25838(5) | 0.0189(2) |
| H8A | 0.650889 | 0.171598 | 0.281446 | 0.023 |
| H8B | 0.591330 | −0.084727 | 0.264762 | 0.023 |
| C9 | 0.15182(9) | 0.82691(19) | 0.60975(5) | 0.0170(2) |
| C10 | 0.16515(10) | 1.00946(19) | 0.55380(6) | 0.0203(2) |
| H10A | 0.208379 | 0.937456 | 0.518756 | 0.024 |
| H10B | 0.214018 | 1.144267 | 0.576365 | 0.024 |
| C11 | 0.04188(10) | 1.10059(18) | 0.51580(5) | 0.0198(2) |
| H11A | 0.003364 | 1.190566 | 0.549783 | 0.024 |
| H11B | 0.053645 | 1.210421 | 0.477512 | 0.024 |

*U*_eq_ is defined as 1/3 of the trace of the orthogonalized *U_ij_* tensor.

## Table 1 Anisotropic displacement parameters [Å^2^] for TXA_Adipic_0m. The anisotropic displacement factor exponent takes the form: −2π^2^[ *h*^2^*(a*)*^2^*U*_11_ *+ k*^2^*(b*)*^2^*U*_22_ + … + *2hka*b*U*_12_ ]

| **Atom** | ***U*_11_** | ***U*_22_** | ***U*_33_** | ***U*_23_** | ***U*_13_** | ***U*_12_** |
| --- | --- | --- | --- | --- | --- | --- |
| O1 | 0.0209(4) | 0.0481(5) | 0.0242(4) | −0.0017(4) | 0.0051(3) | 0.0098(4) |
| O2 | 0.0224(4) | 0.0298(4) | 0.0172(4) | −0.0016(3) | 0.0075(3) | 0.0032(3) |
| O3 | 0.0260(4) | 0.0175(4) | 0.0212(4) | 0.0015(3) | 0.0109(3) | 0.0026(3) |
| O4 | 0.0314(4) | 0.0204(4) | 0.0195(4) | 0.0010(3) | 0.0106(3) | 0.0037(3) |
| N1 | 0.0216(5) | 0.0184(4) | 0.0160(4) | −0.0010(3) | 0.0069(3) | −0.0009(3) |
| C1 | 0.0196(5) | 0.0183(5) | 0.0190(5) | −0.0010(4) | 0.0058(4) | −0.0004(4) |
| C2 | 0.0172(5) | 0.0197(5) | 0.0170(5) | −0.0003(4) | 0.0055(4) | 0.0010(4) |
| C3 | 0.0264(6) | 0.0236(5) | 0.0194(5) | 0.0034(4) | 0.0082(4) | 0.0086(4) |
| C4 | 0.0317(6) | 0.0209(5) | 0.0196(5) | 0.0046(4) | 0.0123(4) | 0.0065(4) |
| C5 | 0.0182(5) | 0.0181(5) | 0.0167(5) | −0.0008(4) | 0.0053(4) | 0.0004(4) |
| C6 | 0.0222(5) | 0.0196(5) | 0.0178(5) | 0.0021(4) | 0.0061(4) | 0.0047(4) |
| C7 | 0.0257(5) | 0.0207(5) | 0.0185(5) | 0.0033(4) | 0.0092(4) | 0.0041(4) |
| C8 | 0.0212(5) | 0.0210(5) | 0.0155(5) | 0.0001(4) | 0.0056(4) | 0.0030(4) |
| C9 | 0.0160(5) | 0.0186(5) | 0.0162(5) | 0.0000(4) | 0.0027(4) | −0.0006(4) |
| C10 | 0.0221(5) | 0.0191(5) | 0.0207(5) | 0.0031(4) | 0.0067(4) | −0.0001(4) |
| C11 | 0.0241(5) | 0.0166(5) | 0.0189(5) | 0.0022(4) | 0.0045(4) | 0.0010(4) |

## Table 1 Bond lengths and angles for TXA_Adipic_0m

| **Atom–Atom** | **Length [Å]** |
| --- | --- |
| O1–C1 | 1.2125(14) |
| O2–C1 | 1.3171(13) |
| O3–C9 | 1.2759(13) |
| O4–C9 | 1.2499(12) |
| N1–C8 | 1.4899(13) |
| C1–C2 | 1.5163(13) |
| C2–C3 | 1.5232(14) |
| C2–C7 | 1.5374(15) |
| C3–C4 | 1.5307(14) |
| C4–C5 | 1.5290(15) |
| C5–C8 | 1.5268(13) |
| C5–C6 | 1.5321(14) |
| C6–C7 | 1.5336(13) |
| C9–C10 | 1.5171(14) |
| C10–C11 | 1.5322(15) |
| C11–C11^#1^ | 1.527(2) |
|  |  |
| **Atom–Atom–Atom** | **Angle [°]** |
| O1–C1–O2 | 123.98(9) |
| O1–C1–C2 | 123.96(10) |
| O2–C1–C2 | 112.05(9) |
| C1–C2–C3 | 112.09(9) |
| C1–C2–C7 | 110.61(8) |
| C3–C2–C7 | 110.16(9) |
| C2–C3–C4 | 110.81(9) |
| C5–C4–C3 | 111.24(9) |
| C8–C5–C4 | 112.36(8) |
| C8–C5–C6 | 109.57(8) |
| C4–C5–C6 | 110.51(9) |
| C5–C6–C7 | 112.05(9) |
| C6–C7–C2 | 110.24(8) |
| N1–C8–C5 | 111.97(9) |
| O4–C9–O3 | 122.26(9) |
| O4–C9–C10 | 119.13(9) |
| O3–C9–C10 | 118.60(9) |
| C9–C10–C11 | 111.35(8) |
| C11^#1^–C11–C10 | 112.21(11) |

Bonds and angles to hydrogen atoms were omitted.

Symmetry transformations used to generate equivalent atoms:

#1: -X, 2-Y, 1-Z;

## Table 1 Torsion angles for TXA_Adipic_0m

| **Atom–Atom–Atom–Atom** | **Torsion Angle [°]** |
| --- | --- |
| O1–C1–C2–C3 | 13.90(16) |
| O2–C1–C2–C3 | −166.24(9) |
| O1–C1–C2–C7 | −109.46(12) |
| O2–C1–C2–C7 | 70.40(12) |
| C1–C2–C3–C4 | 178.03(9) |
| C7–C2–C3–C4 | −58.36(12) |
| C2–C3–C4–C5 | 57.55(13) |
| C3–C4–C5–C8 | −177.72(9) |
| C3–C4–C5–C6 | −54.99(12) |
| C8–C5–C6–C7 | 179.03(9) |
| C4–C5–C6–C7 | 54.69(12) |
| C5–C6–C7–C2 | −55.94(12) |
| C1–C2–C7–C6 | −178.27(9) |
| C3–C2–C7–C6 | 57.26(12) |
| C4–C5–C8–N1 | −58.32(12) |
| C6–C5–C8–N1 | 178.43(8) |
| O4–C9–C10–C11 | −61.97(13) |
| O3–C9–C10–C11 | 117.45(10) |
| C9–C10–C11–C11^#1^ | −54.89(14) |

Bonds and angles to hydrogen atoms were omitted.

Symmetry transformations used to generate equivalent atoms:

#1: -X, 2-Y, 1-Z;

## Table 1 Hydrogen bonds for TXA_Adipic_0m

| **D–H⋯A [Å]** | **d(D–H) [Å]** | **d(H⋯A) [Å]** | **d(D⋯A) [Å]** | **<(DHA) [°]** |
| --- | --- | --- | --- | --- |
| O2–H2A⋯O3 | 0.946(16) | 1.620(16) | 2.5416(10) | 163.4(15) |
| N1–H1A⋯O3^#1^ | 0.926(16) | 1.856(16) | 2.7794(12) | 175.2(14) |
| N1–H1A⋯O4^#1^ | 0.926(16) | 2.517(16) | 3.0916(13) | 120.5(12) |
| N1–H1B⋯O4^#2^ | 0.911(17) | 1.848(17) | 2.7553(13) | 173.1(15) |
| N1–H1C⋯O1^#3^ | 0.914(16) | 2.047(16) | 2.9181(13) | 159.0(13) |

Symmetry transformations used to generate equivalent atoms:

#1: 0.5+X, 0.5-Y, -0.5+Z; #2: 0.5+X, 1.5-Y, -0.5+Z; #3: 0.5-X, -0.5+Y, 0.5-Z;

## Bibliography

[1]	Bruker, *SAINT*, *V8.40B*, Bruker AXS Inc., Madison, Wisconsin, USA.

[2]	L. Krause, R. Herbst-Irmer, G. M. Sheldrick, D. Stalke, *J. Appl. Cryst.* **2015**, *48*, 3–10, doi:10.1107/S1600576714022985.

[3]	G. M. Sheldrick, *Acta Cryst.* **2015**, *A71*, 3–8, doi:10.1107/S2053273314026370.

[4]	G. M. Sheldrick, *Acta Cryst.* **2015**, *C71*, 3–8, doi:10.1107/S2053229614024218.

[5]	C. R. Groom, I. J. Bruno, M. P. Lightfoot, S. C. Ward, *Acta Cryst.* **2016**, *B72*, 171–179, doi:10.1107/S2052520616003954.

[6]	D. Kratzert, *FinalCif*, *V144*, https://dkratzert.de/finalcif.html.
